# A Full-Spectrum Generative Lead Discovery (FSGLD) pipeline via DRUG-GAN: A multiscale method for drug-like/target-specific compound library generation

**DOI:** 10.1126/sciadv.aei5257

**Published:** 2026-10-28

**Authors:** Beihong Ji, Matthew Brock, Yuhui Wu, Yuemin Bian, Xibing He, Yue Liu, Yixuan Hao, Haocheng Tang, Junmei Wang

**Affiliations:** ^1^ Department of Pharmaceutical Sciences and Computational Chemical Genomics Screening Center, School of Pharmacy, University of Pittsburgh, Pittsburgh, PA 15261, USA.; ^2^ Hangzhou Institute of Medicine, Chinese Academy of Sciences, Hangzhou, 310018 Zhejiang, China.; ^3^ School of Medicine, Shanghai University, 200444 Shanghai, China.

## Abstract

We present the Full-Spectrum Generative Lead Discovery (FSGLD), a deep learning-driven pipeline for efficient drug lead identification. FSGLD integrates generative modeling with molecular docking, molecular dynamics simulations, ligand-residue interaction profile, MM-PBSA, thermodynamic integration (TI), and experimental validation to bridge theoretical design and practical application. The core multiscale DRUG-GAN models enable generative model-guided discovery of drug-like and target-specific compounds across three scenarios: (i) generation of random drug-like compounds, (ii) generation of target-specific compounds, and (iii) generation of target-specific compound series featuring shared chemical structures. In our assessment across three pharmacologically distinct target classes, FSGLD demonstrates encouraging performance in identifying target-specific candidate compounds. In addition, a computational protocol for TI calculations was established to reduce computation time by 80 to 90% while maintaining accuracy. By integrating generative models with in silico and in vitro evaluation techniques, FSGLD reduces the cost of identifying previously uncharacterized yet viable lead compounds, offering remarkable benefits to both academic and industry.

## INTRODUCTION

Traditional drug discovery is a challenging process since it aims to identify target-specific molecules with specific desirable properties from a large chemical space. It cost more than $2.6 billion and takes over 12 years to develop a new drug from initial idea to market approval (*1*, *2*). A fundamental challenge is efficiently navigating the estimated 10^23^ to 10^60^ molecules in chemical space to identify compounds with the desired biological activity, physicochemical properties, and synthetic feasibility (*3*, *4*). For the acceleration of drug discovery and development, traditional computational approaches such as virtual screening (VS) were developed (*5*). However, although these in silico methods can accelerate drug discovery, they are generally limited to screening existing compound libraries, which represent only a tiny fraction of the vast drug-like chemical space (*6*, *7*). Therefore, identification of previously unexplored drug-like chemicals from the huge chemical space is urgently needed.

Artificial intelligence (AI) has come to the view of researchers and gradually being integrated into all kinds of computer-aided drug design approaches, especially in our effort of expanding drug-like chemical space. At the early stage, Simplified Molecular Input Line Entry System (SMILES) string, a textual descriptor of a molecule, was applied to develop deep generative models for drug design. Until today, SMILES is still the commonly adopted representation of a molecule for generative models (*8*–*11*). However, SMILES was not designed for capturing the structural similarity between molecules. For example, two different SMILES strings might represent molecules with the same chemical structures (*12*, *13*). Alternatively, molecular graph, for which molecular validity can be easily assessed, can also be applied to describe molecular structures in generative modeling (*13*–*16*). Nevertheless, graph-based generative models remain computationally demanding, particularly for large molecules, owing to the increasing complexity of graph construction and message passing (*12*). Moreover, some graph generation frameworks, especially graph translation models, are restricted to producing molecules with the same or similar numbers of atoms as the input molecules. More recently, three-dimensional (3D) molecular representations emerge, where the atomic feature space is represented by atoms with spatial coordinates alongside charges and atom types (*17*). However, these methods can be computationally demanding and may not necessarily enhance the quality of generative outcomes.

In this study, we decided to use simple and plain molecular fingerprint (FP) as the molecular descriptor to build the generative models. While the molecular FP representation has an intrinsic limitation, i.e., it is difficult and unambiguously translate the generated FPs into molecular structures, it entails some advantages over the other representations as well. This simple representation is more suitable to quickly generate large amounts of FPs. When FP-based generative modeling is in combination with fast similarity search, one can narrow down the search space and quickly identify the most promising candidates. This combination not only improves the efficiency of molecular generation but also increases the likelihood of identifying synthetically accessible candidates by retrieving molecules from existing chemical databases, where compounds have typically been synthesized or are commercially available. Representative works devoted to developing deep learning models using FP as input features in drug development and discovery are listed as follows. Kadurin *et al.* (*18*) adopted molecular FP as input features to develop a deep adversarial model for identification and generation of compounds using available chemical and biological data. Bian *et al.* (*19*) used four kinds of molecular FPs, Molecular ACCess System (MACCS), Extended-Connectivity Fingerprint 6 (ECFP6), AtomPair, and AtomPair Count to build a generative adversarial network (GAN) to screen out and develop candidate compounds for cannabinoid receptors (CBs).

Many deep generative models have been introduced and successfully applied in de novo drug design, such as recurrent neural network (RNN)–based models, autoencoder (AE)–based networks, GANs, and graph neural network (GNN)–based models. Recently, several continuous generative paradigms—including normalizing flow-based, diffusion-based, and flow-matching–based models—have emerged for molecular generation (*20*, *21*). Building on these advances, many recent 3D molecular generation methods incorporate E(3)-equivariant GNNs to preserve rotational and translational equivariance (*22*). Despite these rapid progress, GANs continue to offer several distinct advantages that make them particularly attractive for molecular design. The concept of GANs was initially proposed by Goodfellow *et al.* in 2014 (*23*), which introduces a competitive framework for two neural network models, the generator and discriminator, being engaged in a dynamic adversarial process. GANs have been successfully applied across a wide range of domains, particularly for generating images, audio, and videos (*24*, *25*). Their first application to de novo molecular design was reported by Guimaraes *et al.* in 2018 (*10*), who extended SeqGAN by incorporating reinforcement learning to develop the Objective-Reinforced Generative Adversarial Network (ORGAN) for molecular generation. In 2018, De Cao and Kipf (*14*) introduced MolGAN model for the generation of small-sized molecules using molecular graphs. Prykhodko *et al.* (*26*) proposed an AE combined with a GAN model for de novo molecular design in 2019. In 2020, Maziarka *et al.* (*27*) developed a CycleGAN-based model that can generate optimized compounds which are structurally similar to the original ones. Also, in the same year, Méndez-Lucio *et al.* (*28*) reported an approach by using conditional GANs to generate molecules based on particular gene expression signatures. In 2022, Abbasi *et al.* (*29*) proposed a feedback GAN framework that incorporates an optimization strategy by integrating encoder-decoder, GAN, and predictor models into a closed feedback loop.

In this study, we built a series of (conditional) deep convolutional GAN [(c)dcGAN] models for generative FP-guided compound discovery. Our main purpose is to find chemicals served as initial hit candidates, which are not only drug-like but also target-specific. Moreover, the compound-series model can also be applied in hit-to-lead optimization via exploring R groups. Those models were collected in our DRUG-GAN platform. We applied this platform in three scenarios: (i) to generate drug-like compounds, (ii) to generate target-specific compounds, and (iii) to generate target-specific compounds containing a predefined maximum common substructure (MCS) for lead optimization. For scenario (ii), CB2 was selected as a representative G protein–coupled receptor (GPCR) target because of its therapeutic relevance, well-characterized ligand landscape, and availability of experimentally annotated ligands. Unlike CB1, which is primarily associated with cannabinoid-induced psychoactive effects, CB2 is predominantly involved in immune and inflammatory regulation, making it an attractive target for evaluating target-specific molecular generation (*30*–*33*).

For all of three scenarios, the deep convolutional neural networks (DCNNs) (*34*–*37*) were applied to construct both the discriminator and generator of a GAN model. It is noted that for the generation of target-specific compounds, a cdcGAN model was used (*38*). Conditional GAN, first proposed by Mirza and Osindero in 2014 (*39*), is an extension of the original GAN concept. This model introduces conditional input to guide the generation of data belonging to a specific class. In our work, we have integrated deep convolutional networks with the framework of conditional GAN to develop conditional dcGAN models for scenarios (ii) and (iii). This advanced model can better guide the generation of target-biased active compounds by including both active and inactive CB2 ligands in the training data. Although cdcGANs have been previously applied in various domains—including image generation, nanophotonic structure design, geological prediction, and fault diagnosis (*40*–*43*)—their application to molecular design remains unexplored. In this work, we investigate the use of cdcGANs for generating target-biased FPs in target-informed ligand discovery. The cdcGAN-generated FPs, including CB2-enriched and scaffold-constrained FP queries, were used to retrieve candidate compounds and compound series from ChEMBL and ZINC databases. These retrieved candidates were further evaluated by a series of hierarchical molecular modeling workflow consisting of multiscale molecular modeling techniques: molecular docking, ligand-residue interaction profiles (LRIPs) using previously developed machine learning–based scoring function (*44*), molecular dynamics (MD) simulation, molecular mechanics-Poisson Boltzmann surface area (MM-PBSA) free energy calculations (*45*–*50*), and thermodynamic integration (TI) alchemical free energy calculations (*51*–*53*).

To the best of our knowledge, Full-Spectrum Generative Lead Discovery (FSGLD) provides a comprehensive pipeline that not only leverages generative models to design drug-like and target-specific compounds but also systematically and efficiently screens and validates those candidate compounds via a hierarchical in silico processes and in vitro experimental validation (Fig. 1). Our pipeline establishes an integrated workflow that bridges the gap between theoretical design and practical application, making it highly efficient and actionable. By combining state-of-the-art generative modeling with robust molecular modeling techniques, we provide a practical and scalable framework that has the potential to reduce the time and cost associated with preclinical drug discovery. The compounds generated by this pipeline are not only theoretical candidates but are also viable for experimental validation, offering tangible benefits for both researchers and industry professionals. Besides FSGLD, several pipelines that combined generative modeling and downstream VS have been established and applied in drug design, which include LambdaZero by Korablyov *et al.* (*54*) and a workflow proposed by Chenthamarakshan *et al.* (*55*) in designing inhibitors of the spike protein and main protease of severe acute respiratory syndrome coronavirus 2 (SARS-CoV-2). These studies highlight the potential of integrating generative models with VS, although their applications have largely focused on individual targets or specific optimization objectives.

**Fig. 1.**
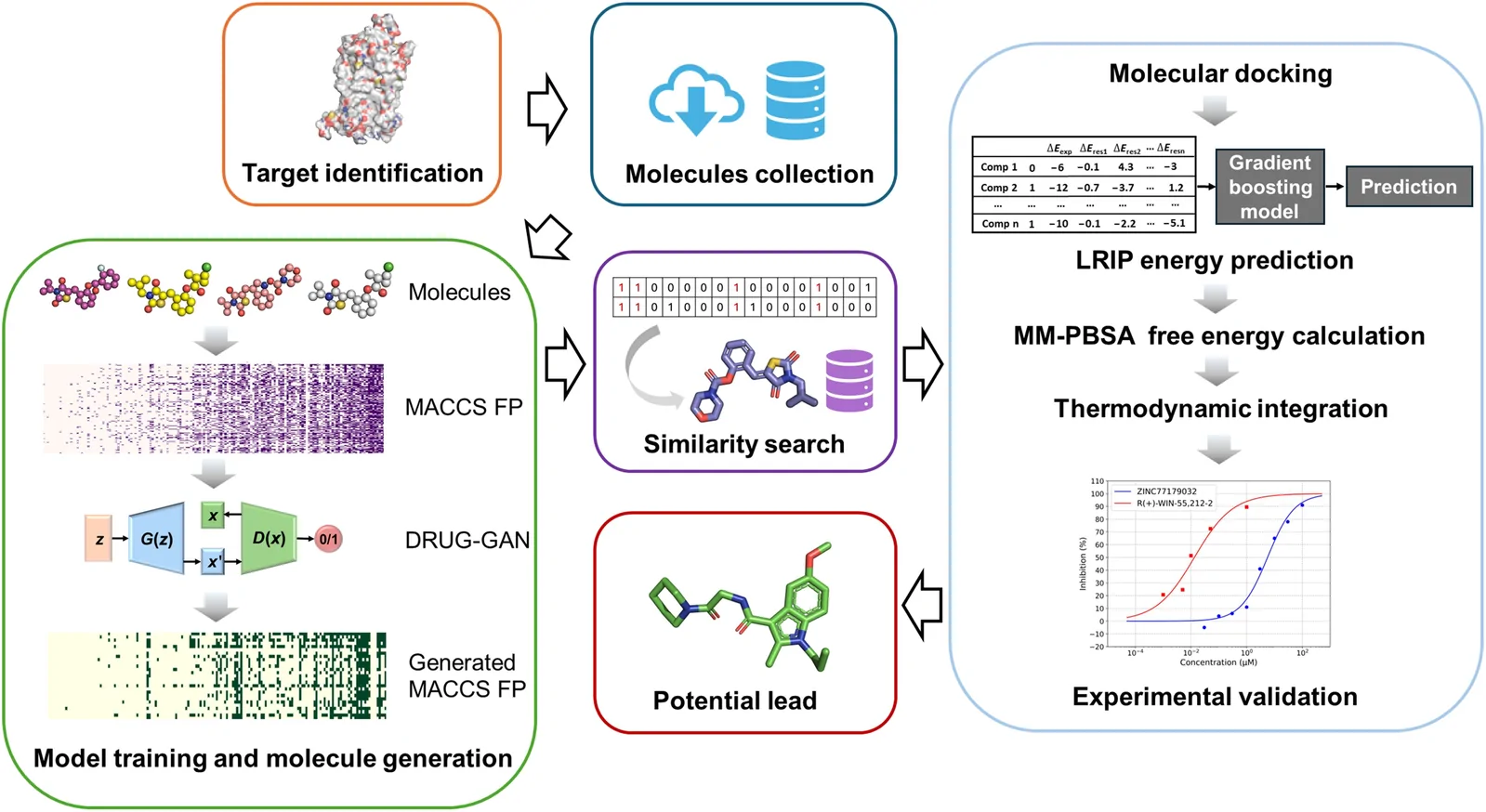
An overview of FSGLD pipeline.

## RESULTS

### Prediction results for supervised discriminators

Before adversarial GAN training, an independent supervised classification task was performed to identify the optimal discriminator architecture. This dataset consisted of 7002 drug-like and 9998 decoy molecules. While the 7002 drug-like molecules were collected from DrugBank (*56*), the 9998 decoy molecules were selected from a prefiltered ZINC (*57*) drug-like screening pool consisting of 19,212,639 compounds. The decoy set was further selected with the restriction that the distributions of molecular weight (MW) and the logarithm of the partition coefficient between water and octanol (log*P*) were essentially similar between the DrugBank drug-like and ZINC decoy datasets. This 17,000 molecule dataset was used exclusively for supervised discriminator evaluation and architecture selection before adversarial GAN training. Given the binary FP-based classification setting, this dataset provided a reasonable scale for initial discriminator comparison, as further supported by the 10-fold cross-validation results.

We trained the discriminator using different DCNN architectures, and the model performance was summarized in Fig. 2 and table S2. In total six DCNN architectures—namely, LeNet 5, AlexNet, ZFNet, VGGNet11, VGGNet13, and VGGNet16—were investigated with the aim to identify the DCNN architecture which can best fit the input features to achieve the best classification accuracy. Figure 2A shows the receiver operating characteristic (ROC) curves on test sets for the six DCNN models. The model performance of a classifier can be measured by the area under the ROC curve (AUC). The AlexNet models achieved the best average AUC value of 0.94, outperforming the DCNN models based on the other architectures (table S2). AlexNet model was first introduced by Krizhevsky and his coworkers (*35*) in the ImageNet competition in 2012. Their AlexNet-based DNN model showed excellent performance and took the first place in the competition. According to the confusion matrixes shown in Fig. 2B, DCNN models trained using the Lenet 5, AlexNet, and ZFNet architectures have better performance than the three VGGNet-based architectures since the former three have much more true positives (TPs) (>550) and true negatives (TNs) (>550), and fewer false positives (FPs) and false negatives (FNs). These results suggested that the number of convolutional layers in a DCNN model is not positively correlated with the model performance.

**Fig. 2.**
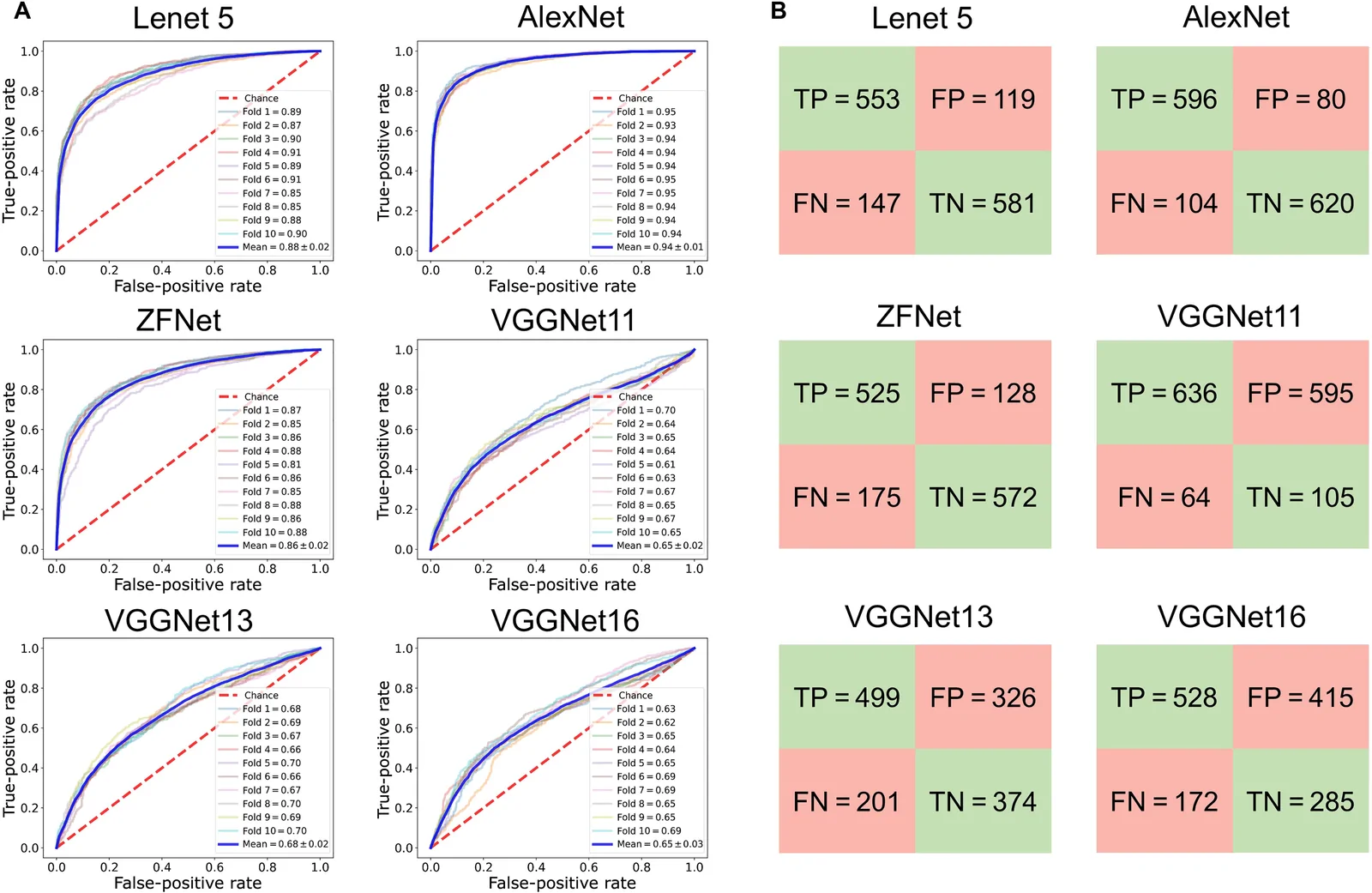
Performance of discriminator models. (**A**) ROC curves of 10-fold cross validation. (**B**) Confusion matrixes list the average values of TPs, TN, FP, and FN.

Besides ROC-AUC, Table S2 also summarized the average values of other key performance metrics, including accuracy, *F*_1_ score, precision, and recall scores, for all DCNN models on the test set. According to this table, AlexNet-based models were ranked no. 1 on all performance metrics except for recall, which were ranked the second place; LeNet 5-based models performed slightly worse with the second-highest AUC, accuracy, *F*_1_ score, and precision scores. It should be noted that these metrics were used to evaluate supervised classification performance for discriminator architecture selection. The quality of the generated FPs was evaluated separately in the following section using generator-level metrics. To provide a non-CNN baseline for comparison, we further implemented a multilayer perceptron (MLP) model trained with the same number of layers and hidden nodes as the AlexNet-based DCNN model under identical training setting. The MLP achieved strong performance (table S2 and fig. S1), confirming that it is a competitive baseline.

### Scenario I: Generation of drug-like compounds

The supervised discriminator used for architecture evaluation was used solely to identify optimal network architecture and was not the final discriminator used in the GAN. The best-performing architecture, AlexNet, was subsequently adopted as the discriminator backbone in DRUG-GAN, where the generator and discriminator were jointly optimized through adversarial training. The generator architecture was constructed by reversing the convolutional architecture of the discriminator. Specifically, the dcGAN was trained using 7002 DrugBank molecular FPs as real samples for the discriminator, while the generator learned to produce synthetic FPs from random noise. After convergence, 10,000 molecular FPs were generated, and their quality was evaluated by comparing their distributions with those of the DrugBank FPs in the training set. In addition, 10,000 randomly sampled molecules from the ZINC database were used as a baseline for comparing molecular property distributions with those of the DRUG-GAN–retrieved compounds.

One intrinsic limitation of FP-based generative modeling is the difficulty of directly converting generated molecular FPs into molecular structures. To address this limitation, we used similarity searching against existing compound databases to identify structurally related molecules corresponding to the generated FPs. Among the 10,000 generated FPs, 493,201, 30,851, and 928 compounds in the ZINC database exhibited maximum structural similarities with Tanimoto scores (TSs) ≥ 0.70, ≥ 0.75, and ≥ 0.80, respectively. The progressive reduction in retrieved compounds reflects the increasingly stringent structural similarity requirements imposed by higher TS thresholds. A TS cutoff of 0.70 captures a broad structural neighborhood within the ZINC chemical space, whereas a cutoff of 0.80 identifies only compounds with substantially greater FP overlap with the generated FP queries.

We conducted a comprehensive comparison of DRUG-GAN with three published generative models [ORGAN, OR(W)GAN, and ORGANIC] in terms of uniqueness, novelty, diversity, similarity, and validity in both FP and SMILES spaces. Notably, these published models directly generate SMILES strings and therefore bypass this limitation of DRUG-GAN, which produces molecular FPs as output. For a fair comparison in FP space, the SMILES generated by the three published models were converted into MACCS FPs, whereas DRUG-GAN produced its FPs directly.

In MACCS FP space, DRUG-GAN outperformed the benchmark models across several key metrics (Table 1). In particular, DRUG-GAN achieved perfect uniqueness and novelty scores (both 1.00), indicating that all generated FPs were distinct and absent from the training data. Its diversity score (0.73) was close to those of ORGAN (0.77) and ORGANIC (0.76) and higher than that of OR(W)GAN (0.63). Furthermore, DRUG-GAN achieved the lowest average similarity to the 7002 reference drugs (0.25), indicating its ability to generate structurally distinct FPs while preserving drug-like characteristics.

**Table 1.** Evaluation of DRUG-GAN and published generative models using 10,000 generated samples in both FP and SMILES spaces.

MACCS FP space					
Model	Uniqueness	Novelty	Diversity	Similarity	
ORGAN	0.66	0.83	0.77	0.20	
OR(W)GAN	0.39	0.84	0.63	0.19	
ORGANIC	0.76	0.82	0.76	0.22	
DRUG-GAN	1.00	1.00	0.73	0.25	
SMILES space					
Model	**Uniqueness**	**Novelty**	**Diversity**	**Similarity**	**Validity**
ORGAN	0.70	0.90	0.85	0.52	0.75
OR(W)GAN	0.48	0.95	0.79	0.44	0.88
ORGANIC	0.83	0.92	0.87	0.51	0.85
DRUG-GAN	0.77	1.00	0.89	0.35	1.00

Consistent with the FP-space evaluation, DRUG-GAN also showed strong performance in the SMILES space (Table 1). DRUG-GAN retained a novelty score of 1.00, demonstrating that none of the decoded molecules appeared in the training set. As expected, the uniqueness decreased from 1.00 in FP space to 0.77 in SMILES space because multiple FPs can decode to the same molecular structure. Nevertheless, the uniqueness remained higher than those of ORGAN and OR(W)GAN and was only slightly lower than that of ORGANIC. DRUG-GAN also maintained high diversity (0.89) while exhibiting relatively low similarity to the reference drugs (0.35), consistent with the goal of expanding chemical space without sacrificing chemical plausibility.

Notably, the published SMILES-based generative models did not produce exclusively valid molecules. Of the 10,000 generated SMILES, ORGAN, OR(W)GAN, and ORGANIC yielded 8235, 5530, and 9146 valid, unique molecules, respectively. In contrast, DRUG-GAN achieved 100% validity because each generated FP was converted into a chemically valid compound through database retrieval. This validity metric, however, is intrinsically linked to the database-retrieval strategy and is therefore not directly comparable to the validity of models that generate molecular structures de novo. For DRUG-GAN, validity primarily reflects the availability of chemically valid compounds corresponding to the generated FPs, whereas uniqueness, novelty, diversity, and similarity provide more direct measures of the chemical-space characteristics of the retrieved molecules.

We next evaluated the drug-likeness of the generated molecules by calculating key molecular properties for each model, with a randomly sampled ZINC dataset included as a reference. As shown in Table 2 and Fig. 3, most generated molecules satisfied Lipinski’s rule of five. However, DRUG-GAN-retrieved molecules exhibited narrower and more favorable distributions of drug-like properties, particularly for molecules retrieved using the 70% similarity threshold, whereas the random ZINC sample showed broader and less constrained property distributions. To further contextualize these results, we analyzed 2252 Food and Drug Administration (FDA)–approved drugs with MWs ranging from 100 to 1000 Da. The mean MW, log*P*, and topological polar surface area (TPSA) values of these approved drugs were 358.0, 2.8, and 89.3, respectively. DRUG-GAN–retrieved molecules exhibited comparable drug-like characteristics, with mean values of 382.9 Da for MW, 2.0 for log*P*, and 76.0 Å^2^ for TPSA. These values indicate slightly larger but less polar molecules relative to the FDA-approved drug set, suggesting potentially favorable physicochemical properties. The property distributions of DRUG-GAN–retrieved molecules were also comparable to those of the 7002 drug-like molecules used to train the DRUG-GAN discriminator (mean MW, log*P*, and TPSA of 356.7 Da, 1.6, and 102.1 Å^2^, respectively), indicating that the generated FP queries preserved key characteristics of the training chemical space.

**Table 2.** Comparison of drug-like properties of generated compounds by DRUG-GAN, published generative models, and randomly sampled ZINC compounds. For each method or reference set, molecular properties were calculated from 10,000 compounds and are presented as mean values. Values in parentheses represent SDs.

Model	MW	Log*P*	TPSA	QED
DRUG-GAN (TS ≥70%)	382.92(63.70)	1.984(1.68)	76.01(22.14)	0.66(0.16)
DRUG-GAN (TS ≥75%)	392.98(65.62)	1.59(1.80)	88.32(25.53)	0.61(0.16)
DRUG-GAN (TS ≥80%)	396.15(66.87)	0.37(2.22)	113.88(26.95)	0.50(0.14)
ORGAN	179.24(66.57)	2.26(1.58)	33.57(24.92)	0.60(0.13)
OR(W)GAN	267.85(124.18)	3.26(1.94)	38.35(32.86)	0.62(0.17)
ORGANIC	177.33(66.38)	1.30(1.94)	55.68(34.22)	0.52(0.14)
ZINC	299.69(98.09)	1.95(3.02)	70.40(49.84)	0.59(0.25)

**Fig. 3.**
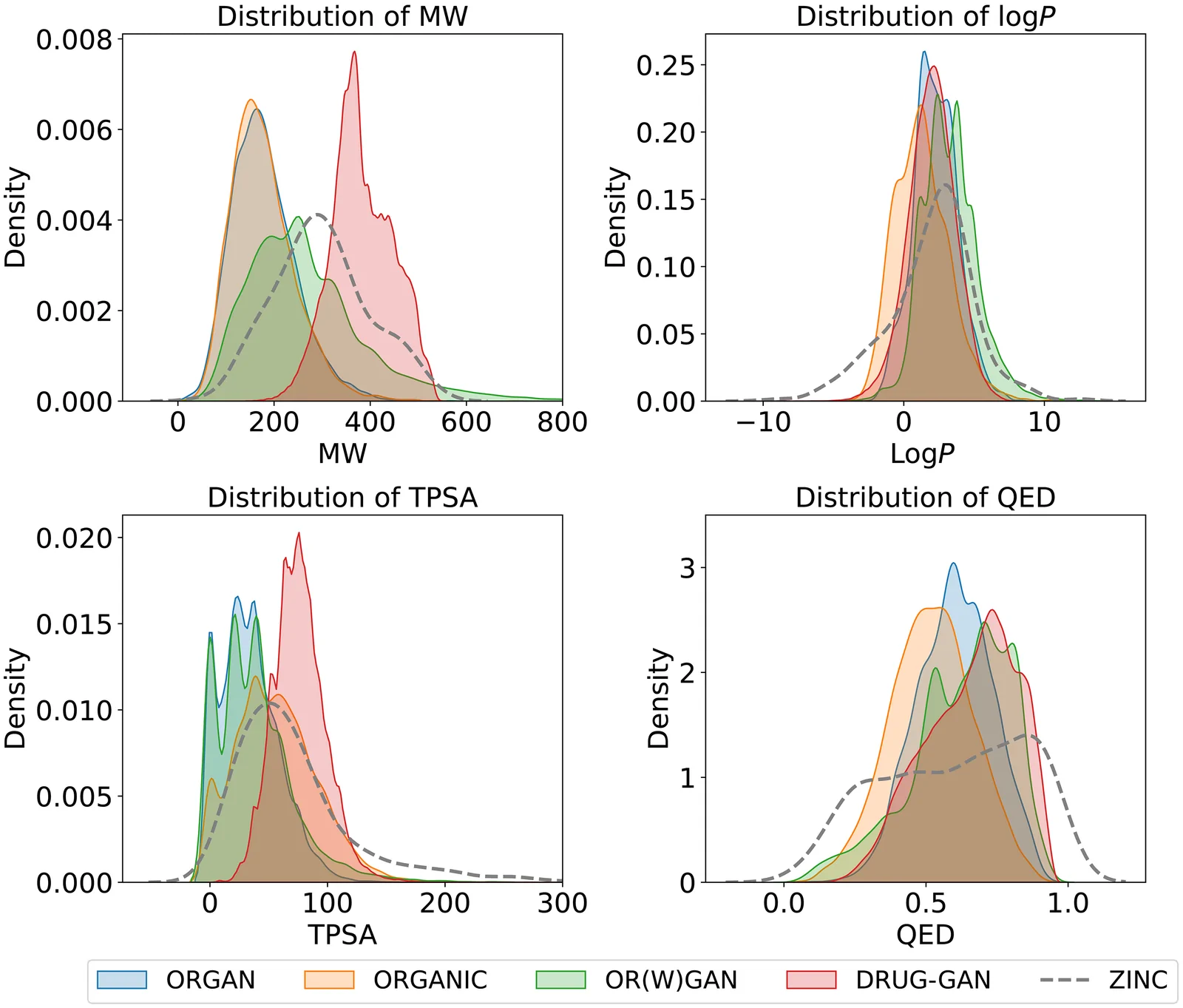
Comparison of molecular property distributions among DRUG-GAN–retrieved compounds (TS ≥ 70%), compounds generated by three published generative models and randomly sampled ZINC compounds.

In contrast, molecules generated by the three benchmark models exhibited substantially lower MW, ranging from 177 to 267 Da. The difference in property distributions likely reflects the distinct generation strategies used by DRUG-GAN and the benchmark models. DRUG-GAN learns and samples in FP space derived from drug-like molecules and subsequently identifies structurally valid compounds through similarity-based retrieval, whereas benchmark models directly generate molecular structures in SMILES space. The FP-based generation and similarity-guided retrieval strategy used by DRUG-GAN facilitates the identification of ZINC compounds with characteristic drug-like features, including scaffold complexity and functional group composition. These features may contribute to the moderately higher MW and TPSA values observed for the retrieved compounds. Collectively, these results demonstrate that DRUG-GAN generates effective FP queries capable of identifying novel, diverse, drug-like, and chemically valid compounds from chemical databases.

Selected top candidates ranked by their similarity to ZINC compounds and quantitative estimate of drug-likeness (QED) values are summarized in tables S3 and S4 and illustrated in Fig. 4. These candidates exhibited favorable physicochemical properties, including balanced MW, lipophilicity, and polar surface area, supporting their drug-like profiles. In addition, the selected compounds displayed substantial structural diversity, indicating that DRUG-GAN can effectively explore a broad chemical space. Together, these results demonstrate that the integration of FP-based generation with similarity-guided retrieval provides a viable strategy for constructing high-quality VS libraries for drug discovery.

**Fig. 4.**
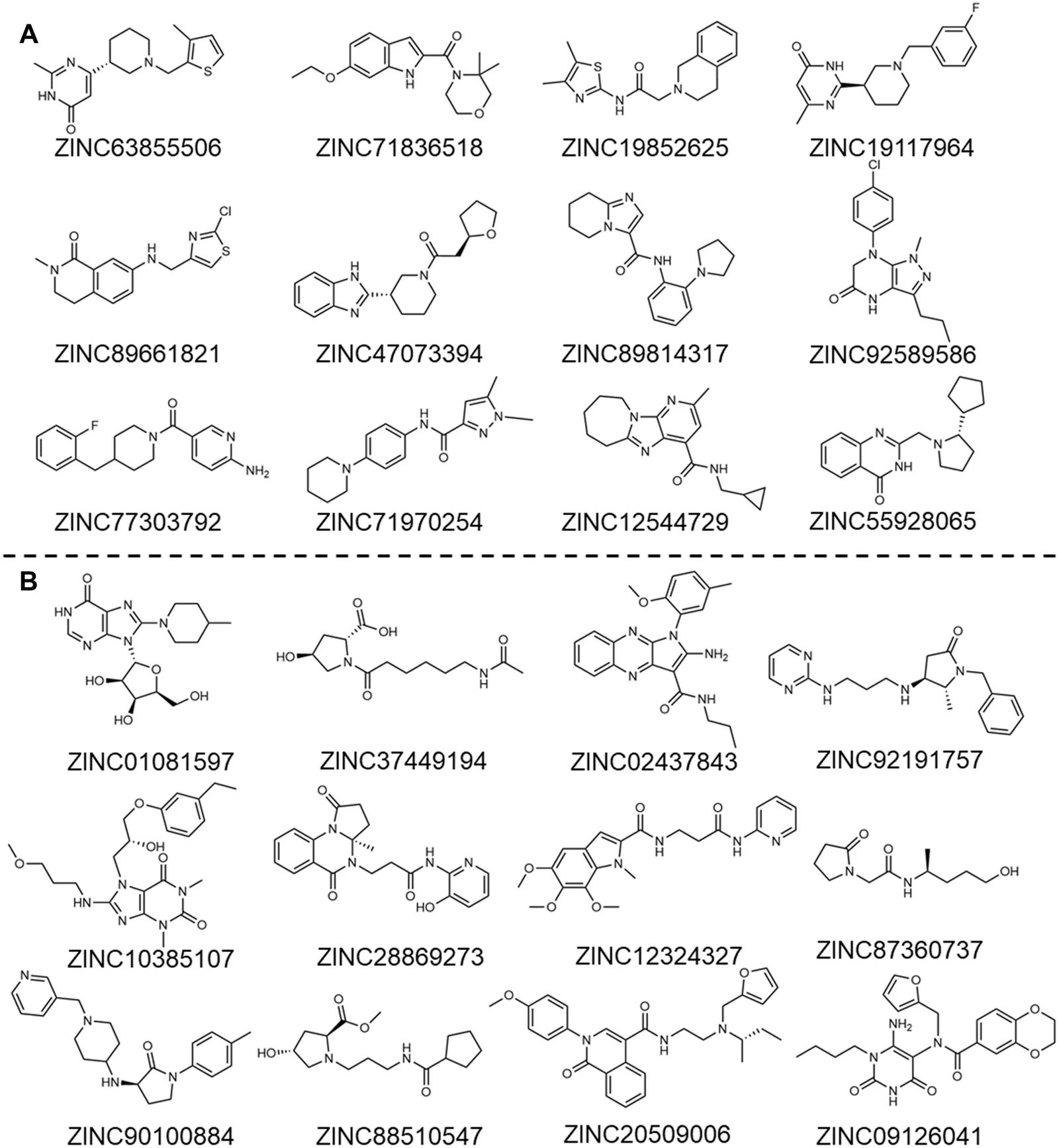
Drug-like candidates retrieved from the ZINC database. Representative drug-like candidates retrieved from the ZINC database based on QED (**A**) and high Tanimoto similarity to generated FPs (**B**).

### Scenario II: Target-specific compound generation

In addition to generating drug-like candidates, DRUG-GAN also has the capability to generate target-specific compounds. In the following, we took cannabinoid receptor 2 (CB2) as an example to demonstrate this capability. Unlike scenario I, which used a standard dcGAN model, scenarios II and III used a cdcGAN model trained on two classes of compounds. Specifically, the training dataset consisted of 1896 active and 2996 inactive CB2 compounds. The numbers of compounds retrieved from ChEMBL and ZINC databases under different similarity thresholds are summarized in Table 3. Among the 186 ChEMBL compounds retrieved with a Tanimoto similarity (TS) ≥ 85% to the generated FPs, 169 had MWs between 100 and 500 Da. Notably, 104 of these 169 compounds were reported as active CB2 ligands in bioassays (*K*_i_ < 10 μM). The mean *K*_i_ value of these active compounds was 267.22 nM, with values ranging from 0.199 to 5000 nM. The distribution of log*K*_i_ values is shown in Fig. 5A. The enrichment of retrieved compounds with low *K*_i_ values indicates that DRUG-GAN–generated FP queries can effectively recover known CB2-active chemotypes from a target-relevant bioactivity database. However, because the 1896 CB2-active ligands used to train the cdcGAN model were also collected from ChEMBL, some retrieved ChEMBL compounds may overlap with the training set or represent close analogs of known training ligands. Therefore, the ChEMBL-based evaluation primarily serves as a validation of target-specific enrichment and chemotype recovery rather than a measure of molecular novelty.

**Table 3.** The number of searched compounds using different similarity criteria from ChEMBL and ZINC databases.

Database	TS ≥ 0.70	TS ≥ 0.75	TS ≥ 0.80	TS ≥ 0.85
ChEMBL	212,093	38,771	2,517	186
ChEMBL (100 ≤ MW ≤ 500)	151,211	28,669	2,053	169
ZINC	1,583,230	270,875	13,652	161

**Fig. 5.**
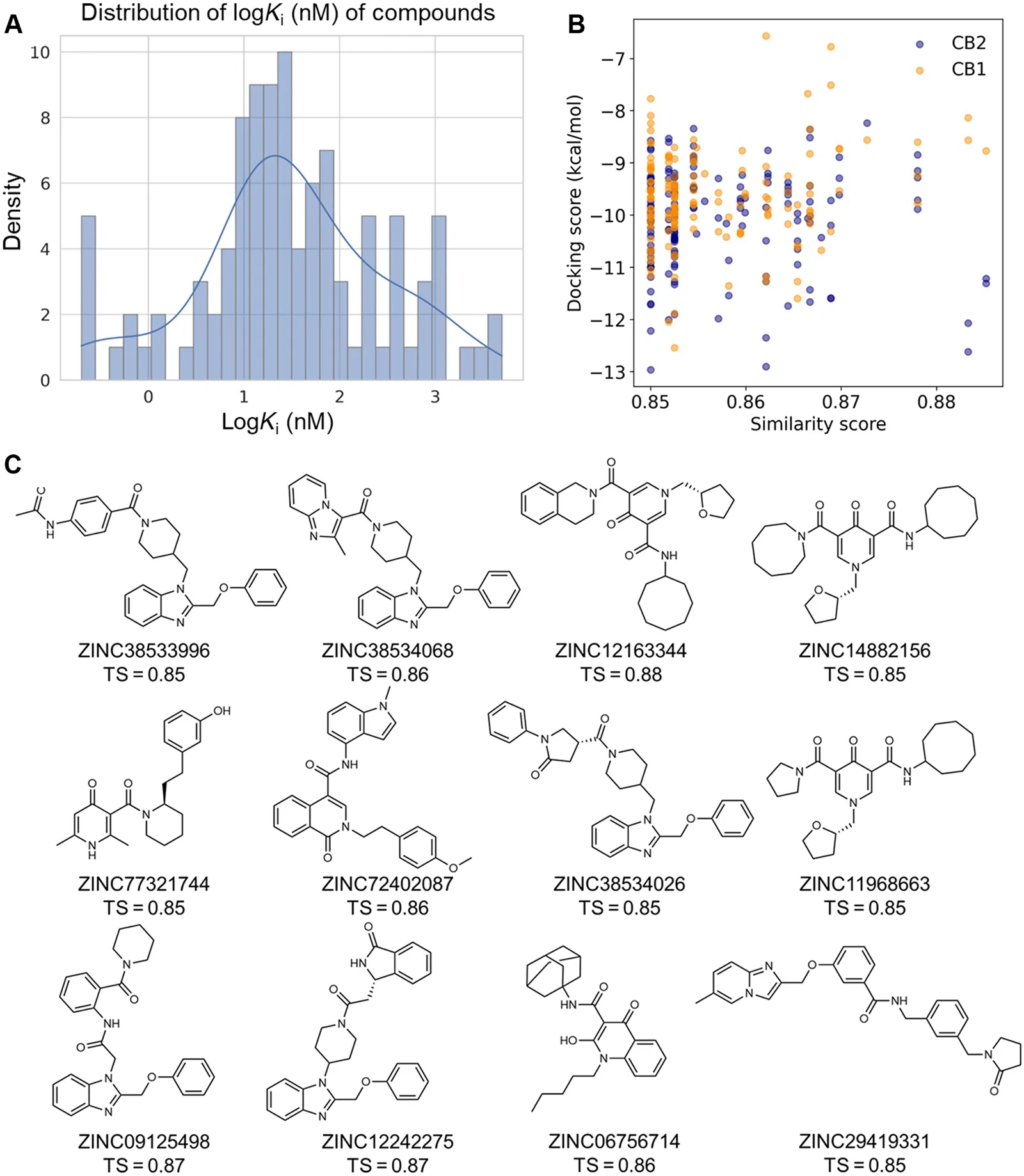
Performance of DRUG-GAN in scenario II. (**A**) Distribution of log*K*_i_ values for CB2-active compounds retrieved from the ChEMBL database. (**B**) Predicted docking scores of ZINC compounds retrieved at the specified TS threshold against CB1 and CB2 receptors. (**C**) Chemical structures of representative ZINC candidates retrieved with TS ≥ 0.85 and prioritized by predicted CB2-binding affinity.

In addition, 161 compounds were retrieved from the ZINC database with TS ≥ 85% to the DRUG-GAN–generated FP queries. To further assess their structural relationship to the CB2-active training ligands, we calculated the maximum FP2-based TS between each retrieved compound and all CB2-active ligands in the training set. The resulting maximum FP2 similarities had a mean value of 0.689 and ranged from 0.374 to 1.000. Among the retrieved compounds, 24 exhibited maximum FP2 similarities greater than 0.85, including ZINC72106265, which was identical in FP2 representation (FP2 similarity = 1.000) to CHEMBL1814458, a reported selective CB2 ligand (*K*_i_ = 0.6 nM) (*58*). In contrast, 51 compounds had maximum FP2 similarities below 0.60, indicating substantial structural divergence from the CB2-active training ligands. Together, these findings suggest that the retrieved library captures both known CB2-active chemotypes and structurally distinct candidates. This conclusion is further supported by quantitative evaluation of the generated FP queries and the retrieved ZINC compounds using the same metrics as those described for scenario I, including uniqueness, novelty, diversity, similarity, and validity in both FP and SMILES spaces (Table 4).

**Table 4.** Evaluation of DRUG-GAN in scenarios II and III. For each model, 10,000 MACCS FPs were generated and evaluated in both FP and SMILES spaces. The SMILES space evaluation was performed using the compounds retrieved from the ZINC database.

MACCS FPs					
Scenario	Uniqueness	Novelty	Diversity	Similarity	
Scenario II	1.00	1.00	0.57	0.40	
Scenario III-MCS i	0.02	1.00	0.06	0.51	
Scenario III-MCS ii	0.02	1.00	0.06	0.50	
SMILES of retrieved samples*					
Model	**Uniqueness**	**Novelty**	**Diversity**	**Similarity**	**Validity**
Scenario II	1.00	1.00	0.29	0.29	1.00
Scenario III-MCS i	1.00	1.00	0.19	0.33	1.00
Scenario III-MCS ii	1.00	1.00	0.12	0.31	1.00

* The SMILES-space evaluation was based on the unique ZINC compounds retrieved for each scenario: 161 compounds in scenario II (TS ≥ 0.85), 32 compounds containing MCS i features in scenario III (TS ≥ 0.75), and 9 compounds containing MCS ii features in scenario III (TS ≥ 0.80). Similarity for the SMILES-space was calculated using FP2 FPs.

The retrieved compounds were subsequently docked against both CB1 and CB2 receptors, and the predicted binding affinities are summarized in Fig. 5B. Overall, the DRUG-GAN–generated FP queries retrieved compounds with favorable predicted binding to CB2. The mean CB2 docking score was −10.10 kcal/mol, compared with −8.29 kcal/mol for the decoy set used to train the discriminator in scenario I. The pairwise comparison of the docking scores for each retrieved compound further suggested a preference for CB2, with a substantial fraction of compounds exhibiting more favorable predicted binding to CB2 than to CB1 (Fig. 5B). Representative top-ranked ZINC candidates selected based on both FP similarity and predicted binding affinity are presented in table S5 and Fig. 5C. For these candidates, the differences between the paired CB1 and CB2 docking scores ranged from 0.67 to 4.49 kcal/mol, with a mean difference of 2.41 kcal/mol. Notably, all selected candidates exhibited more favorable predicted binding to CB2 than to CB1, suggesting a possible CB2 preference during the preliminary docking-based prioritization step.

### Scenario III: Generation of target-specific compound series

To generate molecular FPs constrained by predefined MCS features, we analyzed known CB2 ligands collected from ChEMBL and identified common structural motifs enriched among highly active CB2 ligands (Table 5 and fig. S2). Three representative MCS features were selected: MCS i, corresponding to a quinoline-dione core; MCS ii, corresponding to an indole-carboxamide core; and MCS iii, corresponding to an indole-sulfonamide scaffold. A mask layer was subsequently introduced into the generator to preserve the MACCS FP bits associated with each predefined MCS. Specifically, the MACCS bits corresponding to the selected MCS features were fixed to 1 during FP generation, as described in Materials and Methods section. This constraint converted the unconstrained target-biased FP generation strategy in scenario II into a scaffold-constrained target-biased FP generation strategy in scenario III, ensuring that the generated FPs retained the desired MCS-associated features.

**Table 5.** Summary of MACCS FP-based similarity retrieval and FP2-based MCS validation in scenario III.

MCS feature	Database and subset	TS ≥ 0.70	TS ≥ 0.75	TS ≥ 0.80	TS ≥ 0.85
MCS i 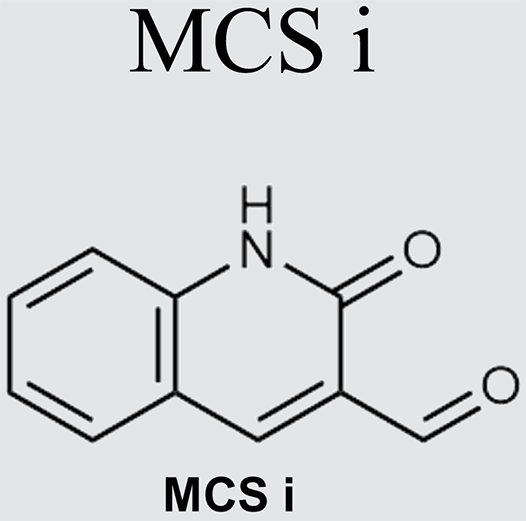	ChEMBL	4,348	1,012	17	0
	ChEMBL (MW: 100–500)	2,240	320	13	0
	ZINC	15,219	2,460	75	0
MCS ii 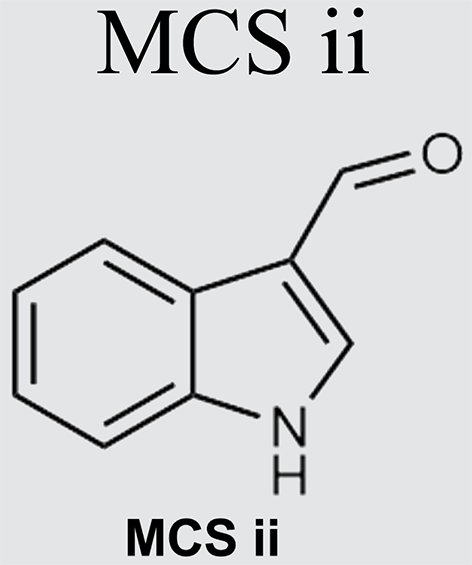	ChEMBL	14,355	2,626	177	2
	ChEMBL (MW: 100–500)	6,382	1,093	77	2
	ZINC	89,105	15,912	911	13
MCS iii 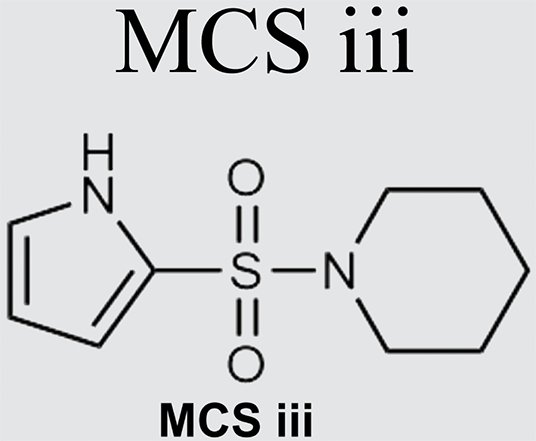	ChEMBL	602	284	58	18
	ChEMBL (MW: 100–500)	323	165	29	0
	ZINC	3,917	1,330	170	5

Using the same similarity-search strategy as in scenario II, compounds were retrieved from the ChEMBL and ZINC databases under four TS thresholds (TS ≥ 0.70, 0.75, 0.80, and 0.85). Because MACCS FPs encode molecules using only 168 predefined structural keys and may not fully capture detailed substructural information, an additional 1024 key FP2 FP-based substructure search was performed to confirm that the retrieved compounds contained the intended MCS features.

For MCS i, 49 ChEMBL compounds were confirmed to contain the predefined substructure and exhibit TS ≥ 0.75 to the generated FP queries. Among these compounds, seven were reported to have high CB2 activity (*K*_i_ < 10 μM). Their measured binding affinities and chemical structures are shown in Fig. 6A. In the ZINC database, 32 compounds retrieved with TS ≥ 0.75 were confirmed to contain the MCS i substructure. Representative candidates and their corresponding TS values are shown in Fig. 6B. Most of these candidates exhibited more favorable predicted docking scores for CB2 than for CB1, suggesting a potential CB2 preference during docking-based prioritization (table S6).

**Fig. 6.**
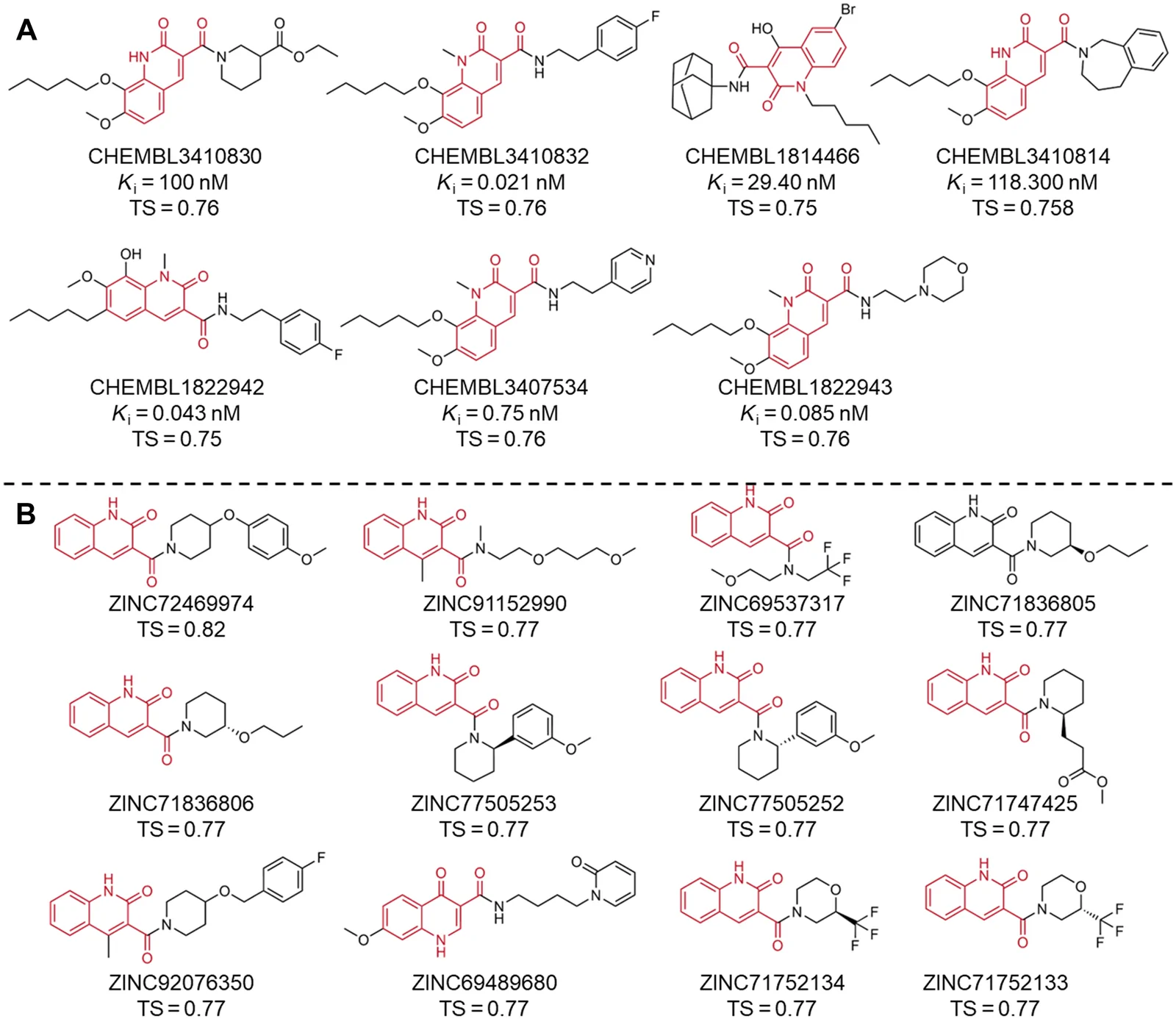
Top-ranked compounds containing MCS i. Top-ranked compounds containing MCS i retrieved from the ChEMBL (**A**) and ZINC (**B**) databases for the CB2 receptor. The red-highlighted region denotes MCS i, a quinoline-dione core.

For MCS ii, 24 ChEMBL compounds were confirmed to contain the predefined substructure and exhibit TS ≥ 0.80 to the generated FP queries. Among them, 8 compounds showed strong CB2 binding affinity (Fig. 7A), further supporting the ability of the scaffold-constrained DRUG-GAN strategy to recover CB2-associated chemotypes. In the ZINC database, nine compounds containing the MCS ii substructure were identified with TS ≥ 0.80 (Fig. 7B). Similar to the MCS i candidates, most of these compounds exhibited more favorable predicted docking scores for CB2 than for CB1, indicating their potential for CB2-focused prioritization (table S7).

**Fig. 7.**
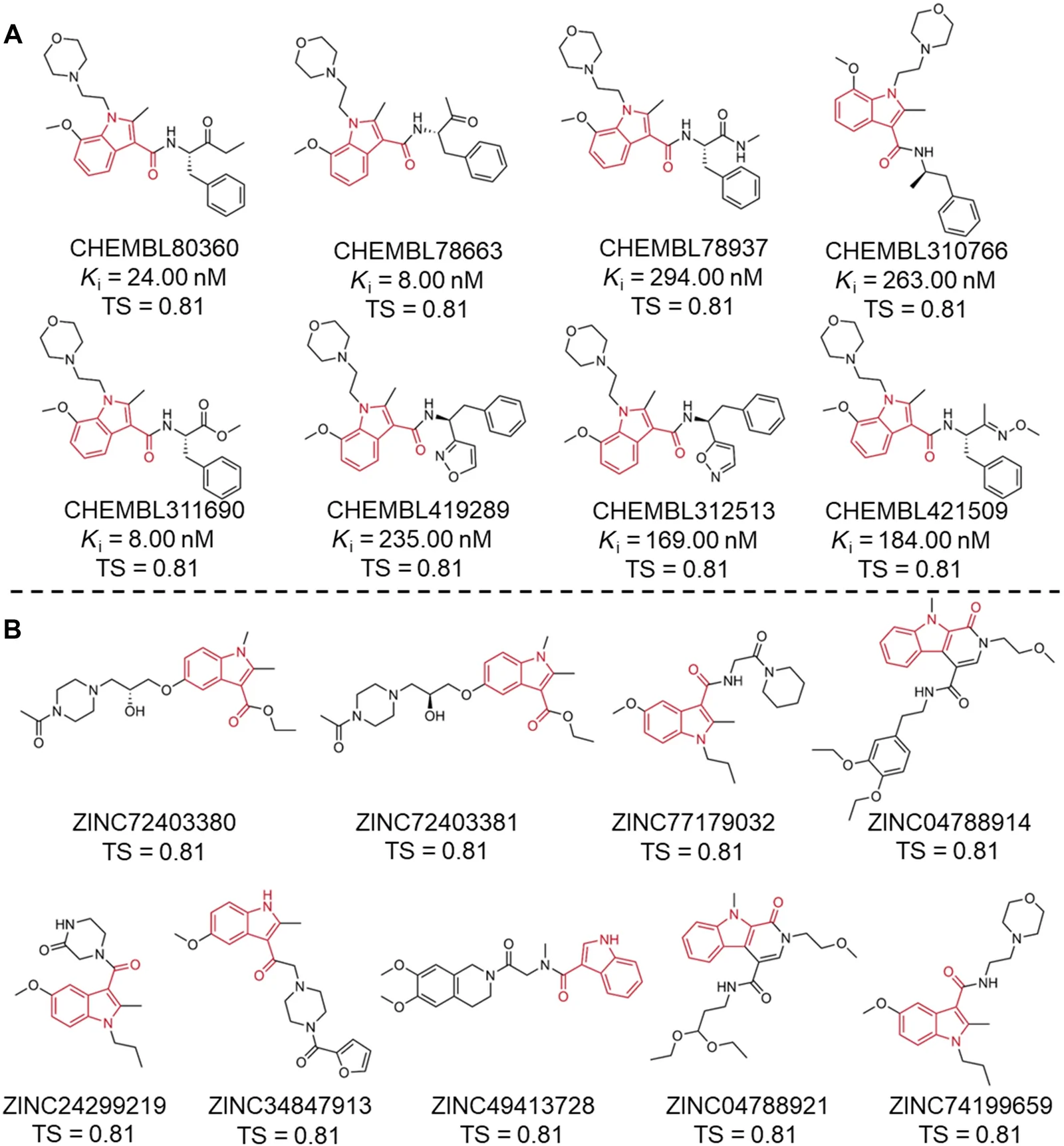
Top-ranked compounds containing MCS ii. Top-ranked compounds containing MCS ii retrieved from the ChEMBL (**A**) and ZINC (**B**) databases for the CB2 receptor. The red-highlighted region denotes MCS ii, an indole-carboxamide core.

For MCS iii, 165 ChEMBL compounds were initially retrieved with TS ≥ 0.75; however, only two compounds were confirmed to contain the exact MCS iii substructure based on FP2 FP analysis (Fig. 8). Furthermore, no ZINC compounds retrieved with TS ≥ 0.75 were confirmed to contain the exact MCS iii substructure. These results highlight the importance of post-retrieval substructure validation, particularly for scaffold-constrained generation based on MACCS FP features.

**Fig. 8.**
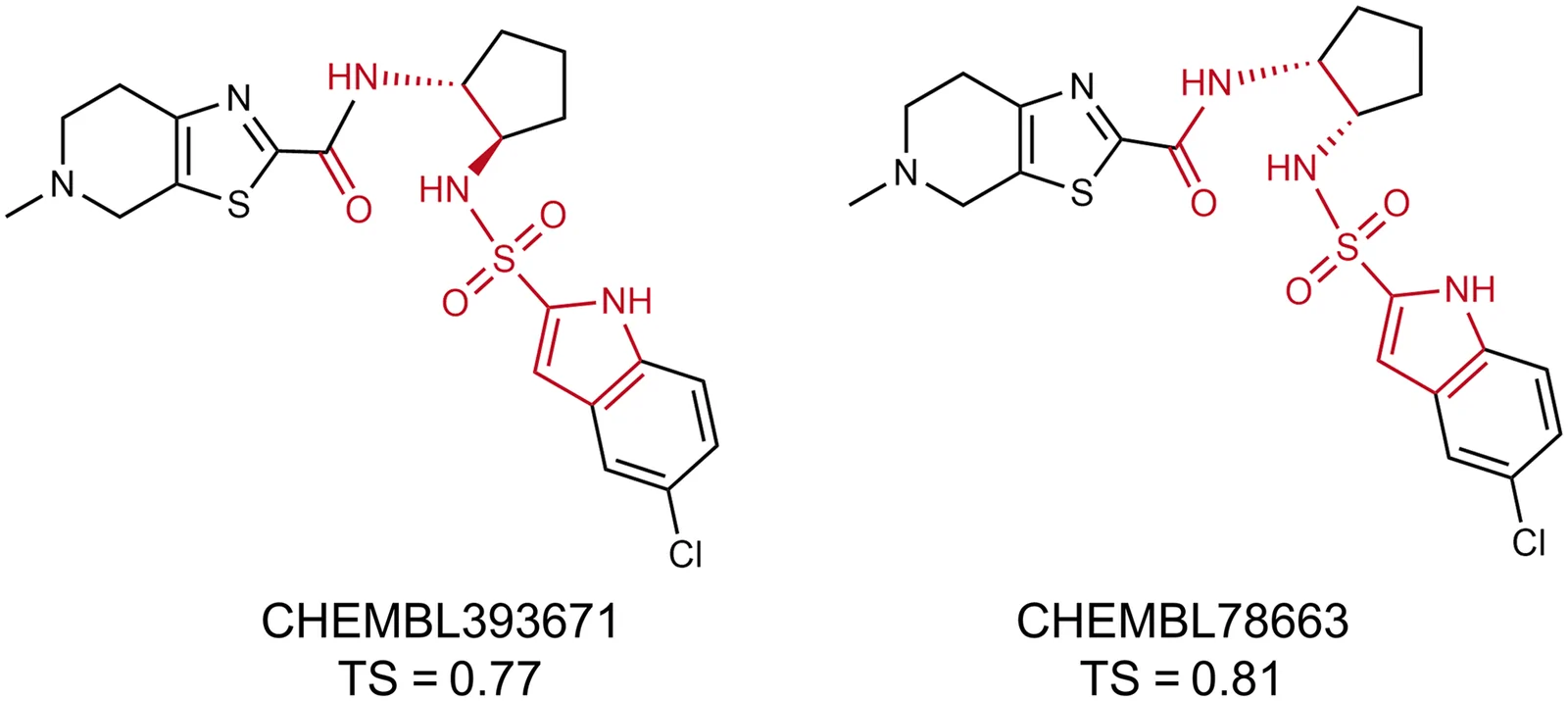
Top-ranked compounds containing MCS iii retrieved from the ChEMBL database for the CB2 receptor. The red-highlighted region denotes MCS iii, an indole-sulfonamide scaffold.

In addition, to evaluate whether the retrieved ZINC candidates represented chemical structures beyond the CB2-active training ligands, we calculated the maximum FP2-based TS of each candidate to the CB2-active compounds used for model training. A similarity threshold of 0.85 was used to identify close structural analogs of the training ligands. For MCS i, the 32 retrieved ZINC candidates exhibited maximum FP2 similarities ranging from 0.629 to 0.820, with mean and median values of 0.743 and 0.750, respectively. For MCS ii, the nine retrieved ZINC compounds showed maximum FP2 similarities ranging from 0.521 to 0.774, with a mean value of 0.635. None of the candidates in either MCS subset showed FP2 similarity greater than 0.85 to any CB2-active training ligand, indicating that the retrieved compounds were not close structural analogs of the known actives. These results demonstrate that the scaffold-constrained DRUG-GAN strategy can recover CB2-relevant chemical features while expanding beyond the chemical space represented by the training ligands. Because no ZINC compounds containing the exact MCS iii scaffold satisfied the retrieval criteria, novelty analysis was not applicable for this subset. Similar to scenario II, the generated FPs and retrieved ZINC compounds were further evaluated using uniqueness, novelty, diversity, similarity, and validity metrics in both FP and SMILES spaces. The detailed results are summarized in Table 4.

### Further computational evaluation and experimental validation of the DRUG-GAN generated and retrieved compounds

In this section, we applied the computational pipeline implemented in FSGLD together with experimental bioassays to evaluate the quality of DRUG-GAN–retrieved compounds in scenario III. The generated compounds underwent a hierarchical validation workflow consisting of molecular docking, LRIP energy prediction, end-point MM-PBSA free energy calculation, alchemical TI free energy calculation, and experimental radioligand binding assays (detailed in Materials and Methods). Note that LRIP (*44*) is a CB2 target-specific scoring function, which was trained using the LRIP to achieve efficient yet accurate binding free energy calculations. Only compounds that passed each computational filter advanced to the subsequent stage. To validate the effectiveness of this hierarchical screening framework, we first applied FSGLD to nine ChEMBL compounds (Fig. 9A) with experimentally measured CB2 binding affinities and compared the computational predictions with the experimental data.

**Fig. 9.**
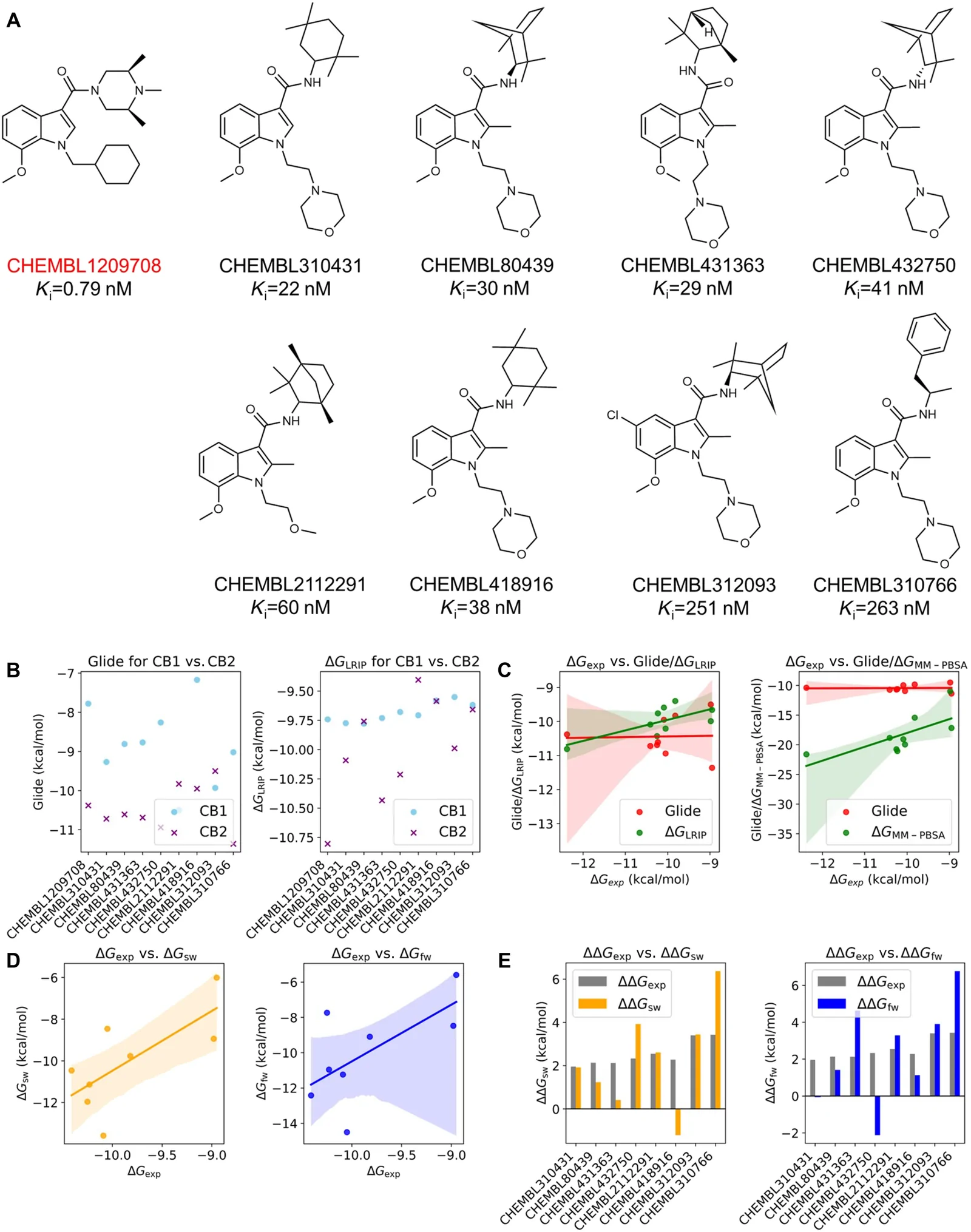
Validation of the computational methods used in the FSGLD pipeline. (**A**) Nine ChEMBL compounds containing the MCS ii substructure used as the validation set. (**B**) Glide docking scores and LRIP-predicted binding free energies for the validation compounds against the CB1 and CB2 receptors. (**C**) Correlations between the experimental binding free energies (∆*G*_exp_) and the predicted values obtained from glide docking scores (red), LRIP (∆*G*_LRIP_, green), and MM-PBSA calculations (∆*G*_MM−PBSA_, purple). (**D**) Correlations between ∆*G*_exp_ and the relative binding free energies predicted by TI using the sw (∆*G*_sw_, yellow) and fw (∆*G*_fw_, blue) protocols. (**E**) Comparison of the relative binding free energies calculated using the two TI protocols.

As illustrated in table S8 and Fig. 9B, the results from both docking and LRIP energy prediction methods indicated that the ChEMBL compounds exhibited a stronger binding affinity for CB2 compared to CB1. This result is expected given those compounds are CB2 ligands. Figure 9 (C and D) highlighted the strong correlations between experimental versus predicted binding energies across several advanced computational approaches, including LRIP, MM-PBSA, and TI. All of them outperformed the Glide docking method, which has a Pearson correlation coefficient (*R*) of 0.03. On the contrary, the *R* values are both 0.69 for LRIP and MM-PBSA methods. As to the most rigorous method, TI achieved *R* values of 0.71 if using the single-window (sw) schedule protocol and 0.64 if using the full-window (fw) schedule protocol. Based on these results, we concluded that LRIP is an accurate and efficient method to enrich top hits from docking screening, and performance of newly proposed sw-TI method achieved a better performance than regular TI.

In the following, we provided more detailed comparisons between the two TI protocols with CHEMBL1209708 as the reference state molecule (Fig. 9E). For sw-TI, only CHEMBL418916 displayed a negative ∆∆*G*_sw_ value, contrary to the experimentally observed positive ∆∆*G*_exp_. In contrast, fw-TI produced incorrect predictions for two compounds, CHEMBL310431 and CHEMBL2112291, where the predicted ∆∆*G*_fw_ values were opposite to the experimental ∆∆*G*_exp_ values. Thus, sw-TI achieved more accuracy in predicting the direction of calculated free energy changes than fw-TI. The better performance of sw-TI measured by both prediction accuracy and correlation to experimental data, in addition to the dramatically reduced computational cost (the cost of sw-TI is only 1/*n* of fw-TI, where *n* is the number of windows in fw-TI), we concluded that in certain cases, sw-TI can serve as an alternative filter of regular TI in the drug screening pipeline.

Through this rigorous screening process, ZINC77179032 emerged as the most promising candidate, exhibiting the strongest predicted binding affinities with both MM-PBSA and TI methods. All the nine top-ranked compounds, illustrated in Fig. 7B, were acquired for binding assay to complete the prediction-verification loop. We first examined the structural similarity of the nine ZINC compounds against ChEMBL database and the CB2 actives. The average TSs are 0.90 ± 0.08 and 0.64 ± 0.10 for the two databases correspondingly. The results suggested that our FSGLD pipeline can generate not only bioactive-like molecules but also chemicals that are structurally dissimilar to the known CB2 actives.

After validating the computational methods in the FSGLD pipeline using the ChEMBL validation set, we applied the complete workflow to ZINC compounds containing the MCS ii substructure. The results of the hierarchical screening—including glide docking, LRIP scoring, MM-PBSA binding free energy calculations, and sw-TI—are summarized in table S9, while the rankings of the nine top candidates are presented in the radar plot in Fig. 10A. Among these candidates, ZINC77179032 emerged as the most promising compound, exhibiting the strongest predicted binding affinities based on both MM-PBSA and TI calculations. The nine highest-ranked compounds, with their structures shown in Fig. 7B, were subsequently purchased for radioligand binding assays to complete the computational-experimental validation loop. We further assessed their structural novelty by comparing them against the ChEMBL database and the set of known CB2-active ligands. The average TS scores were 0.90 ± 0.08 relative to the ChEMBL compounds and 0.64 ± 0.10 relative to known CB2 actives, indicating that the selected molecules closely resemble bioactive compounds while remaining substantially distinct from previously reported CB2 ligands. These results demonstrate that the FSGLD pipeline is capable of identifying compounds that preserve drug-like characteristics yet expand into structurally distinct regions of the CB2 ligand chemical space.

**Fig. 10.**
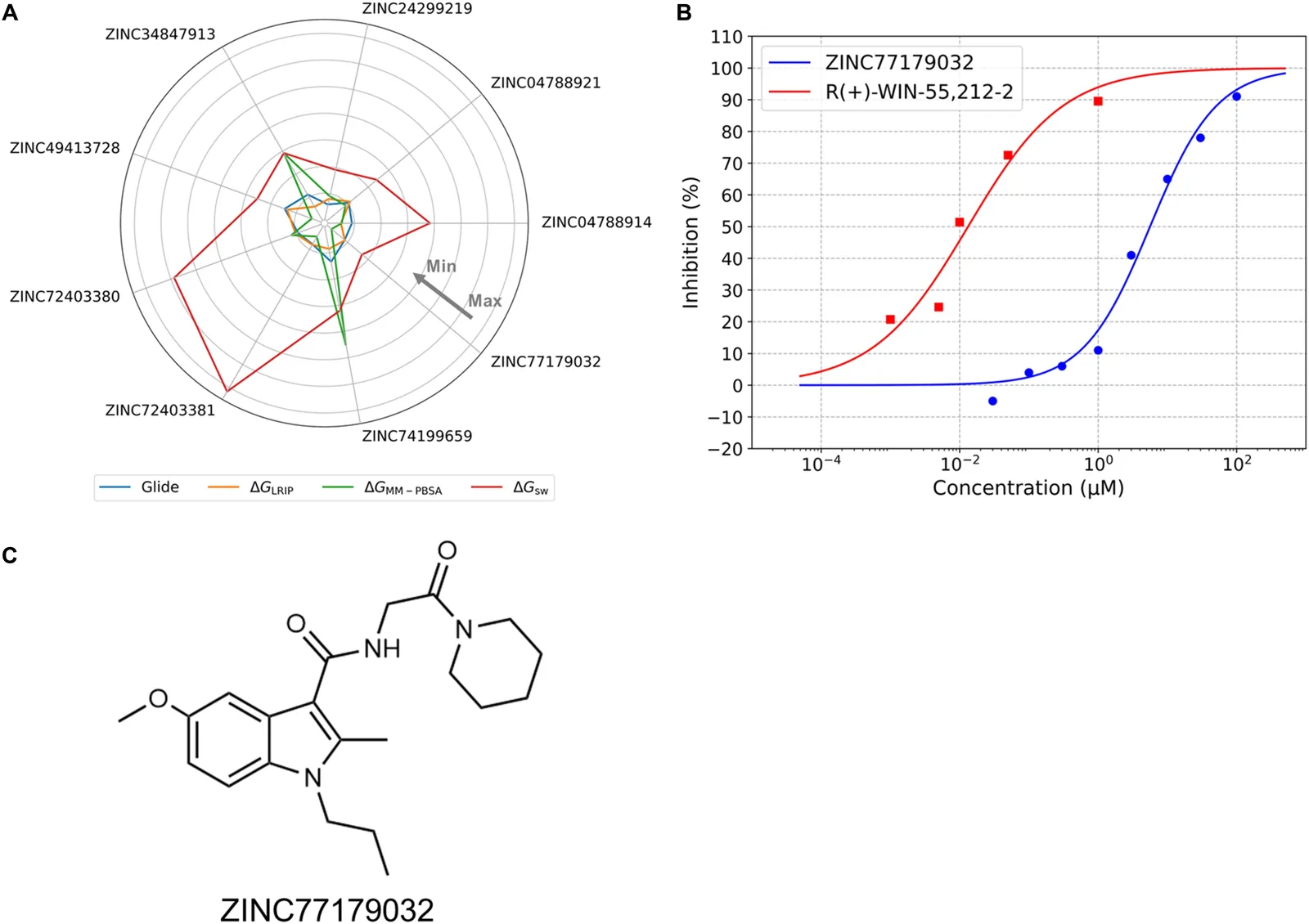
Experimental validation of the FSGLD pipeline using ZINC compounds. (**A**) Radar plot comparing the predicted binding affinities of the top-ranked ZINC compounds obtained from glide docking, LRIP scoring (∆*G*_LRIP_), MM-PBSA (∆*G*_MM−PBSA_), and sw TI (∆*G*_sw_). (**B**) Concentration response curves of ZINC77179032 and the reference ligand R(+)-WIN-55,212-2 in CB2 radioligand binding assays. (**C**) Chemical structure of the experimentally validated CB2 hit ZINC77179032.

Radioligand binding assays revealed that, among the nine tested compounds, only ZINC77179032 exhibited measurable binding to the CB2 receptor, with a *K*_i_ of 3.77 μM and a median inhibitory concentration (IC_50_) of 5.62 μM (Fig. 10B). The remaining eight compounds showed no detectable CB2 binding activity under the assay conditions, precluding reliable determination of their binding affinities. These results identify ZINC77179032 as an experimentally validated initial hit for subsequent lead optimization. To further assess its target selectivity, we evaluated ZINC77179032 against the CB1 receptor using radioligand binding assays. No inhibition of CB1 binding was observed over the tested concentration range of 0.03 to 100 μM, demonstrating a high degree of CB2 selectivity. As an internal assay control, the reference ligand R(+)-WIN-55,212-2 yielded a *K*_i_ of 6.05 nM, in good agreement with historical Eurofins control data (IC_50_ = 5.80 nM, *K*_i_ = 3.90 nM) and previously reported literature values (*59*), confirming the reliability of the assay conditions. Collectively, these results demonstrate that the FSGLD pipeline can successfully identify novel, target-selective initial hits from a vast chemical space and provide experimentally validated starting points for further lead optimization.

### Extension to additional targets for FSGLD validation

To further demonstrate the feasibility of FSGLD in real-world applications, we extended the pipeline to three additional targets: the main proteases of SARS-CoV-2 (SARS-CoV-2 Mpro) and MERS-CoV (MERS-CoV Mpro), and the human epidermal growth factor receptor 4 (HER4). The two main proteases served as additional representative case studies for scenario II. As shown in fig. S3, FSGLD successfully designed and identified highly promising compounds that selectively target either SARS-CoV-2 Mpro or MERS-CoV Mpro. HER4, a receptor tyrosine kinase, was used as an additional case study for Scenario III. For each of the three MCSs shown in fig. S4, we trained a cdcGAN model to generate MACCS FPs, which were then used as queries to retrieve compounds from ChEMBL and ZINC (fig. S5). Among the retrieved ChEMBL compounds, 13 are experimentally validated HER4 inhibitors, including the approved drug dacomitinib, demonstrating the ability of FSGLD to recover known active ligands. Furthermore, the pipeline identified multiple high-ranking ZINC compounds that satisfied all computational screening criteria, providing candidate HER4 inhibitors for future experimental validation (table S18).

To further assess whether FSGLD-derived candidates retain biochemical activity, we experimentally evaluated two top-ranked ZINC candidates against HER4 using a homogeneous time-resolved fluorescence kinase inhibition assay (*60*). Among the tested compounds, ZINC33279960 exhibited dose-dependent inhibition of HER4 kinase activity with an IC_50_ of 8.615 μM as shown in fig. S6. Afatinib, as a clinically approved irreversible inhibitor for HER4, was included as a reference compound which exhibited an IC_50_ of 1.01 nM, consistent with its reported potency (*61*). Although ZINC33279960 is less potent than afatinib, its low-micromolar activity is consistent with that of an initial hit identified through computational screening and supports its potential as a starting point for lead optimization. ZINC33279960 is structurally distinct from previously reported HER4 inhibitors, highlighting the ability of FSGLD to find previously unidentified biologically active scaffolds.

In summary, FSGLD once again produced target-enriched candidates that successfully advanced through hierarchical in silico filters, demonstrating its broad feasibility beyond CB2. Detailed methods and results are provided in the Supplementary Materials.

## DISCUSSION

### FSGLD: An integrated framework for generative lead discovery

In this work, we developed FSGLD, an integrated computational framework for efficient lead compound discovery that combines generative modeling, chemical database retrieval, hierarchical computational screening, and experimental validation. Unlike conventional generative pipelines that directly optimize molecular structures, FSGLD generates molecular FPs and retrieves chemically feasible compounds from large chemical libraries, thereby facilitating efficient exploration of drug-like chemical space while avoiding the challenges associated with directly generating molecular structures. To prioritize promising candidates, the retrieved compounds are evaluated using a hierarchical workflow comprising molecular docking, target-specific LRIP scoring, MD simulations, MM-PBSA binding free energy calculations, and TI, with experimental validation performed for selected compounds. By integrating these complementary components into a unified workflow, FSGLD provides a practical framework for prioritizing lead candidates for subsequent experimental evaluation.

### FP-based generative modeling enables efficient chemical space exploration

The core innovation of FSGLD lies in DRUG-GAN, which generates molecular FPs rather than molecular structures. In this work, DRUG-GAN was implemented in three discovery scenarios, including unbiased generation of drug-like compounds, target-specific lead discovery, and generation of target-focused compound series containing predefined MCS features. Compared with conventional generative models based on SMILES strings or molecular graphs, FP generation provides a compact feature representation that is particularly well suited for learning from relatively small datasets. Our results demonstrate that this strategy effectively captures target-relevant chemical features while enabling efficient exploration of large chemical libraries.

An important distinction between FSGLD and conventional molecular generative models is that the generated FPs serve as search queries rather than final molecular structures. Because MACCS FPs are not uniquely mapped to individual molecules and cannot be directly converted into chemically valid structures, each generated FP is matched against large chemical databases to retrieve structurally similar compounds. This retrieval-based strategy ensures that all downstream candidates are chemically valid and commercially accessible while avoiding common challenges encountered by direct molecular generation, such as invalid structures and synthetic inaccessibility. Consequently, FSGLD should be viewed as a framework for generative query expansion and candidate prioritization rather than direct molecular generation.

We also investigated the influence of discriminator architecture on classification performance. Among the evaluated DCNN architectures, AlexNet consistently outperformed the VGG variants in distinguishing drug-like FPs from decoy FPs. This observation suggests that discriminator performance depends more on architectural design than network depth alone, underscoring the importance of selecting an appropriate neural network architecture for molecular FPs learning.

As an additional baseline, we also evaluated a MLP trained on molecular FPs using the same number of hidden layers and neurons as the AlexNet-based discriminator under identical training settings. Although AlexNet-based CNN achieved only a modest improvement in predictive performance, it required substantially fewer trainable parameters than the MLP. Given that MACCS FPs do not have an inherent spatial organization, the performance gain is unlikely to arise from exploiting local spatial features. Instead, the convolutional architecture primarily functions as a parameter-efficient function approximator, where parameter sharing provides implicit regularization that improves training stability during adversarial learning. Consequently, the observed advantage of the CNN is more plausibly attributed to its superior parameter efficiency and regularization characteristics than to its ability to extract spatially localized features, while the comparable performance of the MLP further confirms that fully connected architectures are also well suited for learning from molecular FP representations.

### From chemical space exploration to target-guided lead discovery

The three DRUG-GAN scenarios demonstrate the versatility of the proposed framework across progressively more challenging lead discovery tasks. Scenario I established that the FP-based generative strategy can efficiently produce diverse and drug-like candidates by coupling molecular FP generation with similarity-based retrieval from large chemical libraries. Compared with representative SMILES-based generative models, DRUG-GAN generated and retrieved compounds with favorable physicochemical property distributions while maintaining high novelty and structural diversity, highlighting the effectiveness of FP representations for exploring drug-like chemical space. Notably, the 70% similarity threshold provided an effective balance between structural diversity and drug-likeness, suggesting that moderate similarity-based retrieval preserves the characteristics of generated FPs while allowing sufficient structural variation.

Scenario II extended the framework from unbiased molecular generation to target-directed lead discovery. By conditioning the generative model on target-specific information, FSGLD successfully recovered known active ligands from ChEMBL while simultaneously identifying structurally distinct candidates from the ZINC database. These findings demonstrate that the learned FP distributions capture target-relevant chemical features and can efficiently guide the exploration of large chemical libraries toward compounds with the desired biological activity.

Scenario III further increased the level of design control by incorporating predefined MCSs, enabling the generation of target-focused compound series suitable for hit expansion and lead optimization. The integration of hierarchical computational screening with prospective experimental validation led to the identification of active compounds, including ZINC77179032 for CB2 and ZINC33279960 for HER4. Although these initial hits exhibit only micromolar potency, they represent experimentally validated starting points for subsequent medicinal chemistry optimization. Collectively, the results across the three scenarios illustrate how FSGLD can support multiple stages of early drug discovery, ranging from unbiased library generation and target-specific hit identification to scaffold-constrained lead expansion.

### Hierarchical screening bridges AI-based generation and experimental validation

A major challenge in molecular generative modeling is translating computationally explored chemical space into experimentally validated starting points for drug discovery. Although recent generative approaches have demonstrated the ability to produce structurally diverse and drug-like molecules, success is often assessed primarily through computational metrics or retrospective analyses. In contrast, FSGLD integrates generative modeling with a hierarchical screening strategy and prospective experimental validation, providing a practical framework that connects chemical space exploration with experimentally testable lead candidates.

An essential component of FSGLD is the hierarchical screening strategy, which progressively prioritizes generated compounds using computational approaches with complementary levels of accuracy and computational cost. Rather than relying on a single scoring function, FSGLD combines molecular docking, target-specific LRIP scoring, MM-PBSA binding free energy calculations, and TI to enrich promising candidates for experimental evaluation. Our results demonstrate that free energy–based approaches provide improved affinity ranking compared with docking alone, while LRIP offers an effective intermediate solution by achieving predictive performance comparable to MM-PBSA at a computational cost closer to conventional docking. This balance between efficiency and accuracy is critical for large-scale VS, where rigorous free-energy calculations remain computationally demanding. Furthermore, our evaluation of TI protocols suggests that simplified single-window TI can achieve comparable performance to conventional multiwindow approaches under appropriate conditions, providing a potential strategy for accelerating free energy–based screening while maintaining predictive capability.

Beyond physics-based scoring, FSGLD also enables flexible integration of machine learning–based prioritization to further improve screening efficiency. To evaluate whether the discriminator itself could serve as an additional filtering module, we retrained the discriminator in a supervised manner using CB2-specific active and inactive compounds in scenario II and incorporated it as the initial filtering step of the FSGLD workflow. Among 10,000 generated molecular FPs, 4298 were predicted as active-like candidates by the discriminator and were subsequently subjected to the remaining hierarchical filters. This early-stage reduction in screening burden did not substantially compromise hit recovery: 149 of the 169 ChEMBL hits and 155 of the 161 ZINC hits with high structural similarity (TS ≥ 0.85) were retained after discriminator filtering. These results demonstrate that learned FP representations can be leveraged not only for molecular generation but also for efficient candidate prioritization, enabling substantial reduction of computational cost while preserving chemical diversity and high-value candidates. Such an adaptive integration of generative and discriminative models may further improve the scalability of AI-driven lead discovery workflows.

FSGLD was prospectively validated through experimental evaluation of computationally prioritized candidates against CB2 and HER4, representing two distinct pharmacological target families. In the CB2 campaign, one active compound (ZINC77179032; *K*_i_ = 3.77 μM, IC_50_ = 5.62 μM) was identified after testing nine candidates, while in the HER4 campaign, ZINC33279960 exhibited kinase inhibition activity (IC_50_ = 8.615 μM) after testing two candidates. These experimentally validated micromolar hits represent suitable starting points for subsequent medicinal chemistry optimization, consistent with the potency range commonly observed for early-stage VS campaigns. Collectively, these results demonstrate that FSGLD extends beyond molecular generation by integrating AI-driven chemical exploration, hierarchical computational prioritization, and experimental validation to identify previously unrecognized and biologically active starting points for drug discovery.

### FSGLD as a complementary strategy to conventional VS

It is important to recognize that experimentally validated micromolar-affinity hits represent common and meaningful outcomes in prospective VS campaigns. A survey of 419 experimentally validated structure-based VS studies reported that only approximately one-quarter of identified hits exhibited submicromolar potency, with the majority of initial active compounds falling within the micromolar range (*62*). Consistent with these observations, conventional docking-based screening campaigns often identify low-micromolar compounds even after evaluating hundreds to thousands of candidates, with relatively low experimental hit rates (*63*). Therefore, the identification of micromolar binders from a small number of FSGLD-prioritized candidates represents a promising outcome for early-stage hit discovery rather than a limitation of the approach.

Compared with traditional screening approaches, generative AI and docking-based VS address different but complementary aspects of chemical discovery. Docking-based screening typically evaluates predefined compound libraries using structure-based scoring functions, providing an effective approach for prioritizing available molecules but remaining constrained by library composition, chemical space coverage, and scoring accuracy. In contrast, FSGLD expands the effective search strategy within database-accessible chemical space by learning target-relevant molecular representations and generating informative FP queries that guide retrieval from large chemical databases. Although the FP-based generation strategy relies on similarity-based retrieval rather than direct molecular construction, it enables exploration of chemical neighborhoods defined by learned molecular features. Consequently, FSGLD can prioritize chemically distinct candidates that may not emerge from conventional library-based screening strategies. Therefore, FSGLD should not be viewed as a replacement for docking-based screening, but rather as a complementary data-driven strategy that expands candidate exploration and facilitates the integration of generative modeling with structure-based evaluation

### Scope, limitations, and future perspectives of FSGLD

In this work, we evaluated FSGLD on two pharmacologically distinct targets, CB2 and HER4, representing GPCRs and protein kinases, respectively. The successful identification of experimentally validated hits across these two target classes provides initial evidence supporting the broader applicability of the framework. Nevertheless, the target-specific cdcGAN strategy relies on available ligand annotations and may be less effective for targets with limited or unreliable activity data. While larger ligand datasets may further improve model performance, extensive training data are not an absolute requirement, as the compact and fixed-length MACCS FP representation enables efficient model training and inference with small- to medium-sized ligand datasets.

Recent advances in structure-conditioned generative models, including diffusion- and flow-matching–based approaches (*64*–*68*), have demonstrated remarkable capabilities for molecular design by incorporating protein structures, ligand poses, or target-specific information. In contrast, cdcGAN does not require high-resolution protein structures or ligand-bound complexes, providing an alternative strategy for targets where structural information is unavailable, uncertain, or difficult to model accurately. Consequently, FP-based cdcGAN and structure-conditioned generative models should be viewed as complementary rather than competing strategies for AI-driven drug discovery. Within this context, the cdcGAN component of FSGLD provides a practical approach for accessing unexplored target-specific chemical space under data-limited conditions.

The performance of the downstream physics-based screening component of FSGLD depends critically on the quality of the protein-ligand complex structures used for binding affinity evaluation. Recently, we demonstrated that modern cofolding methods, including AlphaFold 3 (*69*) and its variations (*70*, *71*), can generate reliable protein-ligand complex structures that provide a robust structural foundation for subsequent physics-based binding affinity prediction (*72*). As protein structure prediction and cofolding methodologies continue to advance, we anticipate corresponding improvements in the accuracy, robustness, and applicability of the FSGLD framework. Future studies will further evaluate DRUG-GAN and FSGLD across a broader spectrum of target families, including ion channels and nuclear receptors, while incorporating advanced few-shot and small-sample learning strategies to further enhance performance in data-limited settings.

In conclusion, FSGLD establishes an integrated framework that combines AI-driven compound generation, hierarchical computational evaluation, and experimental validation for early-stage lead discovery. In this study, FSGLD was evaluated across three representative target classes spanning distinct target families, with prospective experimental validation conducted for CB2 and HER4. By learning molecular FP distributions and leveraging database-based retrieval, FSGLD enables the exploration of target-relevant chemical space while maintaining chemical validity and practical accessibility. Although further evaluation across a broader range of targets will be important to establish its general applicability, the present results demonstrate the potential of FSGLD as a complementary strategy to conventional VS approaches, bridging generative modeling with experimentally validated compound discovery.

## MATERIALS AND METHODS

### Dataset preparation

For scenario I, the drug-like dataset was compiled by collecting 7002 drugs from DrugBank (https://go.drugbank.com/) based on the Lipinski’s rule of five (*73*) and diversity selection. For the decoy set, MW and log*P* were calculated for 19,212,639 molecules in the ZINC drug-like subset (https://zinc.docking.org/) using Open Babel (http://openbabel.org) (*74*). An in-house program was then used to identify 284,552 ZINC compounds with MW and log*P* values most similar to those of the DrugBank compounds. These compounds were subsequently subjected to FP-based clustering using SYBYL-X 2.1.1 to maximize structural diversity. Last, 9998 molecules were selected to form the decoy set. To train and validate the discriminator of dcGAN, all selected drug-like (7002) and decoys molecules (9998) were partitioned into training set and test set using 10-fold cross-validation method (*75*).

For the target-biased compound generation in scenarios II and III, we collected 1896 active (*K*_i_/IC_50_ < 100 nM) and 2996 inactive (*K*_i_/IC_50_ ≥ 100 nM) CB2 compounds from ChEMBL database to train the cdcGAN model. In addition, we also manually checked the chemical structures of collected CB2 active compounds and identified three most common MCSs as shown in Table 5 and fig. S2. During the training, all molecules are represented by MACCS (168 bits) by using Open Babel program version 2.3.1. For further validation, x-ray structure of CB1 (entry code: 5XRA) and constructed homology model of CB2 were obtained and prepared as described elsewhere (*76*).

### Model architecture

DCNN is a deep neural network that is mainly designed for processing image and speech data. In DCNN, to construct the model, convolutional layers were implemented sequentially and each neuron in a convolutional layer receives input from the output of the previous layer. The model was trained to adjust hyperparameters including padding, strides, filter size, learning rate, etc. The dcGAN discriminator used a DCNN consisting of an input dense layer followed by multiple convolutional and fully connected layers. In detail, four major types of DCNN architectures were explored in our work, which are LeNet-5 (*77*), AlexNet, ZFNet (*36*), and VGGNet (*37*). To be noted, VGGNet-based architectures have three types with different number of weight layers. VGGNet11-based and VGGNet13-based models are modified versions of VGGNet16 by dropping off the pound sign and the asterisk sign levels for VGGNet 11 and VGGNet13, respectively. The details of those architectures are described in Fig. 11A. The extracted feature maps were flattened before being passed to the fully connected layers. Hyperparameters—including pooling size, kernel size, padding strategy (*causal*, *same*, or *valid*), and activation function (*tanh*, *softmax*, or *ReLU*)—were optimized during model training. As for the model compilation of the discriminator, the loss function was set to *categorical crossentropy*, kernel regularizer to *L2 penalty*, and the optimizer to *Adam*. In addition, an MLP baseline was implemented by substituting AlexNet’s convolutional blocks with dense layers of the same configuration, under the identical training settings, to provide a non-CNN baseline for comparison.

**Fig. 11.**
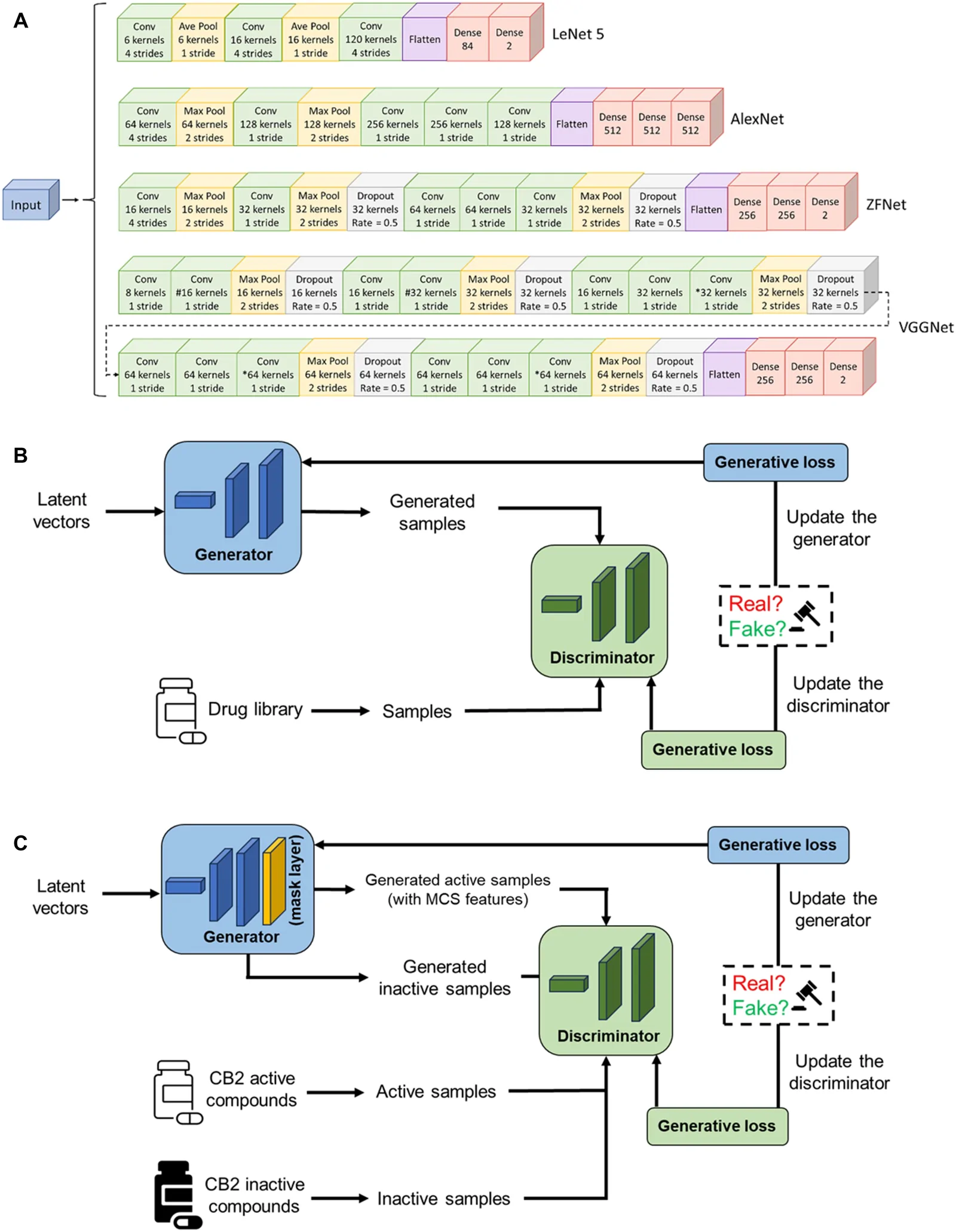
Schematic overview of the DRUG-GAN framework. (**A**) Architecture of the DCNN. (**B**) GAN architecture for generating drug-like molecular FPs. (**C**) GAN architectures for generating target-specific molecular FPs with or without preservation of MCS features.

Then, the generator was created by reversing the convolution process of the discriminator. GANs are generative models that allow for learning data distribution without the need to explicitly define a likelihood function. GANs consist of two competing neural networks: a generative model (*G*) that maps random variables (*z*) drawn from a prior distribution to the data distribution and a discriminative model (*D*) that distinguishes between real data (*P*_data_) and data generated by *G*. The two components of a GAN model, the generator and discriminator, were trained simultaneously to achieve an objective described by the following formula

$$\underset{G}{\text{min}}\underset{D}{\text{max}}E_{x∼P_{\text{data}}\left(x\right)}\left[\text{log}D\left(x\right)\right]+E_{z∼P_{z}\left(z\right)}\left(\text{log}\left\{1-D\left[G\left(z\right)\right]\right\}\right)$$

The two networks engage in a minimax game, where *G* aims to produce realistic samples to fool *D*, while *D* learns to correctly classify real data versus generated ones. This adversarial process allows *G* to improve its generation of realistic data over time.

Figure 11 (B and C) schematically illustrates the dcGAN and cdcGAN architectures developed for molecular generation with different design objectives. In both architectures, the discriminator was implemented as a DCNN and trained on molecular FPs to distinguish real molecules from those generated by the generator. The dcGAN was trained using a single class of real drug-like FPs, whereas the cdcGAN was conditioned on two biologically defined classes comprising active and inactive CB2 ligands. The generator comprised four convolutional layers with batch normalization and *leaky ReLU* activation. Both the generator and discriminator were optimized using the *Adam* optimizer with the *categorical cross-entropy* loss function. The learning rates were set to 10^−6^ for the discriminator and 10^−3^ for the generator.

In scenario III, the objective is to design CB2 molecules containing MCS structures. To achieve this goal, we introduced a mask layer, *lambda layer*, into the architecture of the generator network. This mask layer is designed to ensure that the generated CB2 FPs retain the feature bits of the MCS, so that those MCS structures will likely appear in the designed molecules. To construct this mask, we first delineated the MCS features from a predefined dataset (MCS i, MCS ii, and MCS iii), creating a binary variable (*mask*) where MCS feature bits were set to 1 and an inverse variable (*unmask*) where non-MCS feature bits were set to 1. This approach can be mathematically represented as

$$\text{mask}\left[i\right]=\left\{ \begin{array}{c} 1\;\text{if}\;i\in \text{MCS}\;\text{features}\\ 0\;\text{if}\;i\notin \text{MCS}\;\text{features} \end{array}\right.$$

unmask[i]=1−mask[i]

In the architecture of the generator, the *lambda layer* was appended to the last convolutional layer, and the output of the *lambda layer* is calculated using the following formula

lambda layer(x)=x×unmask+mask

where *x* is the raw output of the last convolutional layer. This operation ensures that the MCS feature bits are preserved in the generated FP while allowing flexibility in other bits. The output from this *lambda layer* thus becomes the final output of the generator, ensuring that each generated FP adheres to the MCS constraints. The incorporation of this mask layer represents a substantial enhancement to the basic cdcGAN architecture, allowing for the generation of chemically relevant compounds with desired structural features, essential in drug lead optimization processes.

### Training and sampling

Before adversarial training, we conducted an independent supervised classification study to identify an appropriate discriminator backbone for DCNN architecture. The discriminator was trained as a supervised binary classifier using DrugBank molecular FPs as positive samples and ZINC decoy FPs as negative samples. All given FPs were separated into 10-folds for training and testing using the StratifiedKFold, a Scikit-learn (*78*) module built in Python (*79*). Each of the 10 subsets was iteratively taken as the test set to evaluate the model generated using the rest of the nine subsets. All DCNN architectures shown in Fig. 11A were systematically evaluated to identify the best-performing discriminator architecture for the DRUG-GAN framework. Each architecture was trained for 100 epochs.

After selecting the discriminator architecture, the dcGAN model was trained adversarially to generate drug-like molecular FPs (scenario I). Unlike the supervised discriminator selection step, adversarial training used only DrugBank FPs as the real data distribution. ZINC decoy FPs were used exclusively for the supervised evaluation and selection of the discriminator architecture. During each epoch of dcGAN training, the discriminator and generator were updated sequentially in two steps. In the first step, only the discriminator was set as trainable and optimized to minimize the discriminative loss. Meanwhile, the generator received randomly sampled noise vectors to generate molecular FPs, which were classified by the discriminator together with real DrugBank FPs. In this step, real DrugBank FPs were assigned real labels, whereas generated FPs were assigned fake labels. In the second step, the discriminator weights were frozen, and the generator was trained to minimize the generation loss.Noise vectors were resampled for FP generation, which were passed through the frozen discriminator and assigned real target labels to update the generator parameters. The model was trained for 800 epochs with a mini-batch size of 32 to allow the adversarial training to reach convergence. Model construction and training for dcGAN was conducted using Keras module (*80*) and Tensorflow (*81*) as the backend built in Python.

For cdcGAN model training (scenarios II and III), the same training procedure and hyperparameters used for the dcGAN models were adopted to maintain a consistent training framework across all DRUG-GAN scenarios. Unlike the dcGAN model, the cdcGAN incorporated class labels corresponding to active and inactive CB2 ligands, allowing both the generator and discriminator to learn class-conditional FP distributions. During target-specific FP generation, the active CB2 class label was provided to the generator to guide the generation of FPs toward the active-ligand distribution. The cdcGAN models were constructed and trained using the Keras module (*80*) with Tensorflow (*81*) as the backend built in Python.

### Evaluation methods for generated molecules

#### Model performance

The performance of the DCNN-based discriminator was evaluated using a series of classification metrics, including the AUC, accuracy, *F*_1_ score, precision, recall, and the confusion matrix. The AUC, accuracy, *F*_1_ score, precision, and recall all range from 0 to 1, with 0 indicating the worst and 1 the best performance. For the ROC curve, an AUC of 0.5 corresponds to random discrimination, whereas an AUC of 1.0 indicates perfect discrimination. The confusion matrix summarizes the classification outcomes in terms of TPs, TNs, FPs, and FNs, with DrugBank-derived drug-like compounds defined as the positive class and ZINC decoy compounds as the negative class. Model performance was assessed using 10-fold cross-validation. In each fold, the model was trained using nine subsets and evaluated using the remaining held-out subset. The evaluation metrics were calculated separately for each fold and then averaged across the 10 folds for comparison among the different DCNN architectures.

#### Scenario I: Drug-like chemical space exploration

To assess the performance of dcGAN model, we performed a quantitative analysis from two aspects for the 10,000 molecular FPs generated from the generator after the training process.

First, the generated MACCS FPs were evaluated using four standard metrics: (i) uniqueness: the ratio of the number of unrepeated molecules to the number of total molecules; (ii) novelty: the ratio of the number of molecules that are not included in the training set to the number of unique molecules; (iii) diversity: $\left(1.0-\frac{1}{N}\sum_{i,j>i}{\text{TS}}_{\mathrm{ij}}\right)$ for all *N* molecule pairs (*i*, *j* > *i*) in the test set, where TS_*ij*_ is the TS (*82*) of molecules *i* and *j*; and (iv) similarity: the average of TS_*ij*_ of all molecule pairs, with molecule *i* being a generated molecule, and molecule *j* in the training set.

In addition, the performance of our dcGAN model was compared with three published models, ORGAN, ORGANIC, and OR(W)GAN for which the Wasserstein-1 W distance was implemented for the discriminator (*83*). We used 10 and 250 epochs to train the discriminator and the generation and 100 epochs to train the GAN model. After training, 10,000 SMILES were sampled from three published models, respectively. For a comparison in the same feature space, these SMILES were converted into MACCS FPs, and the four metrics were computed to enable a consistent comparison across all models.

Second, to ensure chemical validity and enable a fair comparison with SMILES-based approaches, each MACCS FP generated by DRUG-GAN was subjected to a TS search against the ZINC database to identify the most similar valid compound represented by a canonical SMILES string. The detailed implementation and computational cost of this FP-based similarity retrieval procedure are described in the Supplementary Materials. The resulting decoded SMILES set was then directly compared with the SMILES generated by ORGAN, ORGANIC, and OR(W)GAN. Within the SMILES representation space, the four metrics used above—including uniqueness, novelty, diversity, and similarity—were recalculated, together with an additional validity metric representing the proportion of chemically valid structures.

For the DRUG-GAN model, we further evaluated the structural similarity between generated FPs and their corresponding retrieved compounds from molecular databases. Specifically, compounds exhibiting TSs of ≥70%, ≥75%, and ≥80% to the dcGAN-generated FPs were identified by searching the ZINC database. The drug-likeness of the retrieved compounds was then compared with that of the valid SMILES generated by three published models to further assess the performance of dcGAN. Drug-likeness was evaluated based on MW, log*P*, TPSA, and QED properties (*84*).

#### Scenario II: Target-specific chemical space exploration

To evaluate the performance of the cdcGAN model, 10,000 molecular FPs were generated and sampled. Compounds from the ChEMBL and ZINC databases exhibiting TS values of ≥70%, ≥75%, ≥80%, and ≥85% to the generated CB2-targeted FPs were subsequently retrieved. The top-ranked ZINC candidates were further prioritized through molecular docking against both the CB1 and CB2 receptors.

For the target-specific generation task, the generated FPs and retrieved ZINC candidates were evaluated using the same metrics applied to the drug-like generation task. Specifically, the 10,000 generated FPs were assessed in FP space based on uniqueness, novelty, diversity, and similarity. The retrieved ZINC candidates were subsequently evaluated in SMILES space using these metrics together with an additional validity metric. All retrieved ZINC candidates included in the SMILES-space evaluation exhibited MACCS-based TS values ≥ 0.85 relative to their corresponding FP queries. Note that the similarity metric reported for the SMILES-space evaluation was calculated separately using FP2 FPs.

To further characterize the structural relationship between the retrieved candidates and the training set, FP2-based TS analysis was performed using FP2 FPs generated by Open Babel. For each retrieved ZINC candidate, the maximum TS to any of the 1896 CB2-active training ligands was calculated and recorded. The ChEMBL-retrieved compounds were used for recovery validation and were not included in the SMILES-space metric evaluation.

#### Scenario III: MCS-constrained target-specific chemical space exploration

For each MCS substructure, 10,000 MCS-conditioned molecular FPs were generated and sampled. Compounds from the ChEMBL and ZINC databases exhibiting TS values of ≥70%, ≥75%, ≥80%, and ≥85% to the sampled CB2-targeted FPs were subsequently retrieved.

Similar to scenario II, the 10,000 generated MACCS FPs in scenario III were evaluated in both MACCS FP space and decoded SMILES space using the same metrics. Different similarity thresholds were applied for compound retrieval according to the predefined MCS constraints. Specifically, for the CB2 case study, TS thresholds of ≥0.75 and ≥0.80 were applied for compounds containing MCS i and MCS ii features, respectively.

Because MACCS FPs contain only 168 predefined structural keys and may not fully capture detailed substructure information, the retrieved compound-series candidates were further converted into 1024-bit FP2 FPs for secondary MCS confirmation. The presence of predefined MCS-associated FP2 features was subsequently assessed based on FP2 representations. The top-ranked ZINC candidates containing different MCS features were then subjected to molecular docking against both CB1 and CB2 receptors. For the confirmed ZINC candidates, the FP2-based TS to each of the 1896 CB2-active training ligands was calculated using FP2 FPs. For each candidate, the maximum similarity to any training ligand was recorded.

Last, the identified hits were evaluated using the hierarchical filtering workflow implemented in FSGLD. Detailed computational procedures—including molecular docking, LRIP energy prediction, MM-PBSA and TI calculations, as well as experimental radioligand binding assays—are described in the Supplementary Materials.

## Supplementary Materials

### This PDF file includes:

Supplementary Text Tables S1 to S18 Figs. S1 to S6 References
