# Supplementary material for: A Full-Spectrum Generative Lead Discovery (FSGLD) pipeline via DRUG-GAN: A multiscale method for drug-like/target-specific compound library generation
Source: Sci Adv. 2026 Oct 28;12(44):eaei5257. doi: 10.1126/sciadv.aei5257 (PMC13715712; doi:10.1126/sciadv.aei5257)
Supplement: Supplementary file 1 [file sciadv.aei5257_sm.pdf]

Science Advances
AAAS

Supplementary Materials for

A Full-Spectrum Generative Lead Discovery (FSGLD) pipeline via DRUG-GAN: A multiscale method for drug-like/target-specific compound library generation

Beihong Ji *et al.*

Corresponding author: Junmei Wang, junmei.wang@pitt.edu

Sci. Adv. **12**, eaei5257 (2026)
DOI: 10.1126/sciadv.aei5257

This PDF file includes:

Supplementary Text
Tables S1 to S18
Figs. S1 to S6
References

Supplementary Text

A brief survey of recent works on generative chemistry published in two Nature journals, *Nature Communications* and *Nature Machine Intelligence*

For recent publications on generative chemistry, we observed that many studies report relatively limited experimental validation and often focus primarily on computational evaluation. In a number of cases, newly proposed generative or predictive models for drug discovery are validated mainly through virtual screening pipelines, including molecular docking, similarity analysis, and physicochemical property evaluation, without performing wet-lab experimental assays(*85-87*).

In studies where experimental validation is included, it is commonly performed on a single representative target, or occasionally two targets, and the identified compounds often exhibit micromolar-level activities. Such activity levels are generally considered reasonable outcomes for early-stage computational hit discovery(*88, 89*).

A similar pattern can also be observed in recent works published in Nature Machine Intelligence, where deep generative models for molecular design are frequently evaluated primarily through computational benchmarks or limited biochemical assays, sometimes involving only one or two drug targets with reported activities in the micromolar range(*64-67, 90*).

The table below summarizes the number of drug targets subjected to experimental validation and the activities of the best hits for five Nature Communications and five Nature Machine Intelligence publications. Notably, only one study (No. 8) validated their models using two drug targets, as we have done in the present work.

Table S1. The number of drug targets subjected to experimental validation and the activities of the best hits for several publications.

No. (*Ref*)	Journal Name	# of drug targets for experimental validation	Best hits activity or hit activity range
1(*85*)	*Nat. Comm.*	0	
2(*86*)	*Nat. Comm.*	0	
3(*87*)	*Nat. Comm.*	0	
4(*88*)	*Nat. Comm.*	1	32.2 nanomolar and 8.06 micromolar
5(*89*)	*Nat. Comm.*	1	1.88 to 20.3 micromolar
6(*64*)	*Nat. Mach. Intell.*	0	
7(*66*)	*Nat. Mach. Intell.*	0	

8(65)	*Nat. Mach. Intell.*	2	32.6 and 72.4 micromolar
9(67)	*Nat. Mach. Intell.*	0	
10(*90*)	*Nat. Mach. Intell.*	1	1.89 micromolar

Extension to Additional Targets for FSGLD Validation

Scenario II- generation of target-biased compounds

Similar to CB2 system, to validate the FSGLD framework in Scenario II. Mpro is essential for viral replication, as it cleaves polyproteins into functional nonstructural proteins that assemble the replication–transcription complex(*91, 92*). Because of its critical role and the lack of closely related human homologs, Mpro has become a highly attractive target for broad-spectrum antiviral drug development(*93*). We used the dataset provided by the ASAP Discovery Antiviral Ligand Pose and Potency Prediction Challenges 2025, hosted on the Polaris platform(*94*). The dataset comprises 2303 protein-ligand complexes, including 1105 for SARS-CoV-2 Mpro and 1198 for MERS-COV Mpro. In the rest of the text, we apply SARS to represent SARS-CoV-2 Mpro and MERS to represent MERS-CoV Mpro.

SARS-CoV-2 Mpro (SARS). The active compounds (652) and inactive compounds (453) in the training set were used as the real data to train the model to generate actives. The numbers of compound hits from ChEMBL and ZINC databases using different similarity criteria are summarized in **Table S13.** Among the compounds retrieved from ChEMBL with at least 70% structural similarity to the generated FPs, 1,316 have molecular weights between 100 and 500. Additionally, 1,050 molecules were retrieved from the ZINC database with a Tanimoto similarity of 75% or higher to the DRUG-GAN-generated FPs. All compounds from ChEMBL and ZINC were subjected to molecular docking to estimate their binding affinities to both SARS and MERS receptors. To enhance the performance of VS, an LRIP model was trained and evaluated on the platform-provided dataset, which was split into training and testing subsets. The best-performing Gradient boosting decision tree (GBDT) model achieved an RMSE of 0.47 kcal/mol, an MAE of 0.38 kcal/mol, a Pearson's correlation coefficient of 0.90, and a predictive index (PI) of 0.88. In contrast, the performance of Glide docking is far inferior to that of the LRIP model with an RMSE of 1.67 kcal/mol, an MAE of 1.25 kcal/mol, a Pearson's correlation coefficient of 0.02, and a predictive index (PI) of 0.03. Next, the predicted binding affinities of those collected compounds were evaluated using the trained LRIP model. As shown in the box plots for ChEMBL

compounds (**Fig. S3a**) and ZINC compounds (**Fig. S3b**), Glide docking scores and LRIP binding affinities are generally more favorable for SARS than for MERS, indicating that our pipeline can design and identify compounds with desirable target selectivity for SARS.

To improve efficiency, we streamlined the FSGLD pipeline by considering only the docking and LRIP filters. Specifically, we selected compounds that ranked in the top 20% of ZINC compounds by docking score and in the top 10% by LRIP binding affinity. By this way, only those very promising molecules will be selected (blue dots in **Fig. S3c**). Chemical structures of these prioritized candidates are shown in **Fig. S3d** and **Table S14**, underscoring the reliability of our approach in identifying SARS-targeting compounds.

MERS-CoV Mpro. The active (448) and inactive (750) compounds in the training set were used as real data to train the model to generate active compounds. The numbers of compound hits from ChEMBL and ZINC databases using different structural similarity criteria are summarized in **Table S15.** Among the compounds retrieved from ChEMBL with at least 70% structural similarity to the generated FPs, 1,774 have molecular weights between 100 and 500. Applying a more stringent structural similarity criterion (TS $\geq$ 0.75) to the DRUG-GAN-generated FP queries yielded 321 molecules from the ZINC database. Compounds from both ChEMBL and ZINC were subjected to Glide docking to estimate their binding affinities to the SARS and MERS receptors, and the results are shown in **Fig. S3e-f**. To enhance the performance of VS, an LRIP model was trained and evaluated on the platform-provided dataset, which was split into training and testing subsets. The best-performing ML model, Gradient boosting decision tree (GBDT), achieved an RMSE of 0.40 kcal/mol, an MAE of 0.31 kcal/mol, a Pearson's correlation coefficient of 0.91, and a predictive index (PI) of 0.86. In contrast, the performance of Glide docking is far inferior to that of the LRIP model with an RMSE of 1.39 kcal/mol, an MAE of 1.11 kcal/mol, a Pearson's correlation coefficient of 0.07, and a predictive index (PI) of 0.12. Next, the predicted binding affinities of those collected compounds were evaluated using the trained LRIP model. As shown in the box plots for ChEMBL compounds (**Fig. S3e**) and ZINC compounds (**Fig. S3f**), Glide docking scores and LRIP binding affinities are generally more favorable for MERS than for SARS, indicating that our pipeline can design and identify compounds with desirable target selectivity for MERS.

To improve efficiency, we streamlined the FSGLD pipeline by considering only the docking and LRIP filters. Specifically, we selected compounds that ranked in the top 20% of ZINC compounds by docking score and in the top 10% by LRIP binding affinity. By this way, only those very promising molecules will be selected (blue dots in **Fig. S2g**). The chemical structures of these prioritized candidates are shown in **Fig. S2h** and **Table S16**, underscoring the reliability of our approach in identifying SARS-

targeting compounds.

Scenario III- generation of target-biased compound series

Human epidermal growth factor receptor 4 (HER4/ErbB4) is a receptor tyrosine kinase in the EGFR/ErbB family that binds neuregulins and other EGF-like ligands. Upon ligand engagement, HER4 dimerizes and activates downstream MAPK/ERK and PI3K/AKT signaling pathways. Beyond signaling, HER4 plays essential roles in development, including cardiac morphogenesis and neuronal maturation, primarily through its interactions with neuregulins(*95*). In oncology, HER4 exhibits context-dependent functions: in breast cancer, certain isoforms act as tumor suppressors by promoting differentiation, whereas in other settings, HER4 overexpression or activating mutations contribute to oncogenesis(*96*). This duality underscores both the biological complexity of HER4 and its potential as a therapeutic target and biomarker. We collected the crystal structure of the ErbB4 Kinase (PDBID: 3BCE) from the Protein Data Bank. As the PDB structure has some gaps, we applied homology modeling to model the structures of the missing residues. We collected 899 inhibitors of HER4 with experimental measurements (K_i, K_d and IC_{50}) from ChEMBL database.

Next, we examined the collected HER4 inhibitors and identified three common structural motifs, MCS i, MCS ii, MCS iii appeared in the active ligands (**Table S17** and **Fig. S4**). We then trained LRIP-SF model for HER4 following an established procedure described elsewhere(*44*), using 899 known inhibitors randomly split into training, validation, and test sets in an 8:1:1 ratio. Again, the GBDT model achieved the best performance (RMSE = 1.14 kcal/mol, MAE=0.67 kcal/mol, R = 0.55, PI = 0.63), significantly outperforming Glide docking (RMSE = 2.46 kcal/mol, MAE=1.80 kcal/mol, R = 0.12, PI = -0.15).

Sequentially, we generated molecules containing a specific MCS after training the cdcGAN model. For MCS i, 2740 ChEMBL compounds were confirmed to contain this substructure and have structural similarity above 80% against the generated fingerprint samples. Encouragingly, nine of these compounds exhibited high binding affinity for HER4 (**Fig. S5a**). Particularly, an approved drug, Dacomitinib, a selective EGFR/HER inhibitor, was among the retrieved molecules, further demonstrating the ability of our pipeline to recover pharmaceutically relevant chemotypes. As to ZINC database, 522 compounds with TS ≥ 0.85 were identified containing MCS i substructure. To further evaluate our pipeline, those ZINC compounds were subsequently subjected to hierarchical screenings using Glide docking and the trained LRIP model. Following the same criteria applied for the MERS-CoV and SARS-CoV-2 case studies, the top 20% of ZINC compounds based on docking scores and the top 10% based on LRIP predictions were identified, and their overlap was selected. This stringent selection

strategy led to one promising ZINC compound being identified (**Fig. S5b-c**).

For MCS ii, 1314 ChEMBL compounds were identified applying the structural similarity criterion of TS ≥ 0.75 against to generate fingerprint samples. Three of these compounds were HER4 inhibitors with decent binding affinity (**Fig. S5d**). As for the ZINC database, 4166 compounds with TS ≥ 0.75 were confirmed to possess the MCS ii substructure. Using the same two-stage filtering strategy, one ZINC compound was identified as worthy of further evaluation (**Fig. S5e-f**).

For MCS iii, 236 ChEMBL compounds were identified containing this substructure and had structural similarity higher than 70% against the generated fingerprint samples. Unlike MCS i and MCS ii, there were no compounds with reported binding affinity for HER4. Three compounds which have the exact MCS iii were shown in **Fig. S5g**. Against the ZINC database, 880 compounds with TS ≥ 0.70 were identified containing the MCS ii substructure. After the two-stage filtering strategy, one ZINC compound was identified as a final hit (**Fig. S5h-i and Table S18**).

Supplementary Methods

Similarity Search. Generated MACCS FPs were used as query vectors to retrieve structurally similar compounds from ChEMBL and ZINC databases. Prior to retrieval, molecules in the searchable databases were converted into MACCS FPs utilizing Open Babel and stored as precomputed FP files. For each generated FP query, Tanimoto similarity was calculated against all reference FPs in the database using a custom C-based program, *tanimoto_ss*. The Tanimoto coefficient between a query FP A and a reference FP B was calculated as

$$T(A,B) = \frac{N_{A,B}}{N_A + N_B - N_{A,B}}$$

where N_A and N_B are the numbers of on-bits in each FP and $N_{A,B}$ is the number of shared on-bits. Molecules with Tanimoto similarity above the predefined cutoff were retained for downstream evaluation. If no molecule satisfied the cutoff, the nearest database molecule with the highest Tanimoto similarity was retained.

For compound-series retrieval in Scenario III, we developed a second C program, *tanimoto_ss_mcs*, which identifies molecules that are both structurally similar to the query and preserve the desired maximum common substructure (MCS). In addition to the Tanimoto similarity score, this program reports the number of missing MCS-associated bits and the fraction of preserved MCS-associated bits. Since MACCS fingerprints contain only 168 predefined structural keys and cannot fully encode detailed substructural information, all retrieved candidates subsequently undergo a

second-round structural verification. Specifically, 1024-bit FP2 FPs and explicit MCS calculations are performed, and molecules that do not contain the desired MCS are removed using our *tanimoto_mcs* program.

The searchable databases contain 2,084,724 valid molecules in ChEMBL and 19,212,639 valid molecules in ZINC. As a representative benchmark, we measured the performance of *tanimoto_ss_mcs* using the CB2 compound series containing MCS *i*, which consists of 225 unique query fingerprints. On a workstation equipped with dual Intel Xeon Gold 6338 CPUs and 256 GB RAM, serial execution required 34.40 ± 0.66 s to search the ZINC database and 3.80 ± 0.11 s to search the ChEMBL database. The physical memory usage was only 1.2 MB per process. Because each query is processed independently, the computational cost scales approximately linearly with both the number of query fingerprints and the database size, while parallel execution provides nearly linear speedup. For example, processing 10,000 query fingerprints using 100 parallel processes would require approximately 1.2 GB of memory and approximately one hour of wall-clock time when searching the ZINC database. These results demonstrate that the similarity retrieval step is computationally lightweight and does not dominate the overall FSGLD workflow. Unlike conventional virtual screening, which requires docking or scoring every molecule in the database, the FSGLD retrieval step performs only binary fingerprint comparisons using 168-bit MACCS fingerprints. Consequently, the computational cost is several orders of magnitude lower and does not represent the rate-limiting step of the overall pipeline.

Glide Docking. Selected compounds were docked to CB1/CB2 targets utilizing the Glide docking program built in the Schrodinger software (Maestro 11.2). Prior to docking, the protein target was prepared using the Protein Structure Preparation Wizard module following the standard procedures, including removing water and co-crystal solvent, filling in missing hydrogen atoms, etc. During geometry optimization, only the newly added hydrogen atoms were allowed to move, with the receptor structure being described by the OPLS force field. A grid was generated for each target, using the co-crystallized ligand to define the center of the binding pocket. No rotatable bonds or constraint groups were applied. Flexible docking was performed using Glide's SP scoring function with the main parameters listed below: ligand partial charge cutoff of 0.15, van der Waals scaling factor of 0.80, and applying reward for intramolecular hydrogen bond formation. The best-scoring docking poses were selected and ranked based on docking scores.

Molecular Dynamics Simulations. Molecular dynamics (MD) simulations were employed to study the dynamics of CB2 receptor-ligand complexes, followed by molecular mechanics-Poisson Boltzmann surface area-WSAS (MM-PBSA-WSAS) binding free energy calculations(*97, 98*). To build the MD system with the protein embedded in a membrane, CHARMM-GUI(*99*) was used to add 240 double-layer 1-

palmitoyl-2-oleoyl-sn-glycero-3-phosphocholine (POPC) lipids, approximate 17,000 TIP3P water molecules(*103*), and 0.15 M Na+/Cl- ions into a rectangular box with dimensions of approximately 95 × 95 × 95 Å.

For energy minimizations and MD simulations, we applied different force fields for different types of molecules, specifically, FF14SB for proteins(*101*), GAFF2 for ligands(*102, 103*), and Lipid14 for lipids(*104*). The atomic partial charges were assigned using the RESP method(*105*), by fitting HF/6-31G* electrostatic potentials generated with Gaussian 16(*106*). Ligand topologies were created using the Antechamber module(107). MD simulations were performed using PMEMD.mpi and PMEMD.cuda from the AMBER 22 suite(*108-110*).

Initially, five rounds of 10,000-step energy minimizations were performed to remove steric clashes, applying positional restraints to the main chain atoms, starting with a force constant of 20 kcal/mol/Å^2 and gradually reducing it to 0. Following the minimizations, the MD simulations were carried out at 298 K and 1 atm with a time step of 2 femtoseconds. To constrain all hydrogen atoms, the SHAKE algorithm(*111*) was applied. For computational efficiency, each protein-ligand system was subjected to a 10 ns MD simulation, and 60 snapshots were evenly collected in the sampling phase (last 6 ns) for further analysis. All systems reached equilibrium in the first 4 ns.

MM-PBSA Binding Free Energy Calculations. Molecular mechanics-Poisson Boltzmann surface area (MM-PBSA) is a widely adopted method for endpoint free energy calculations. To incorporate dynamic effects, MD simulations are commonly used to generate representative conformations. To improve computational efficiency in calculating conformational entropy, which is traditionally computed via normal mode analysis, we developed a solvent-accessible surface area-based method, WSAS, to quickly estimate entropy changes in protein-ligand binding(*98*). Moreover, MM-PBSA binding free energy can be decomposed into distinct interaction components, each of which is calculated from a series of conformational snapshots collected during MD simulations(*112*).

The binding free energy ($\Delta G_{MM-PBSA}$) between a ligand and receptor complex is calculated using the following equation:

$$\Delta G_{MM-PBSA} = \Delta H - T\Delta S = \Delta E_{MM} + \Delta G_{p}^{sol} + \Delta G_{np}^{sol} - T\Delta S$$

Where the ΔE_{MM}term represents the changes in molecular mechanics energy in the gas phase. $\Delta G_{p}^{sol}$is the polar solvation free energy, while ΔG_{np}^{sol} is the nonpolar solvation free energy, the $T\Delta S$ is the entropy change of the ligand-target system during the binding process. For each MD snapshot, the binding free energy of each ligand was

computed, with detailed energy decompositions performed on all 60 snapshots collected during the sampling phase.

LRIP Free Energy Prediction. We employed the previously developed machine learning (ML)-based prediction models using ligand-residue interaction profiles (LRIP) to predict binding free energies(*44*) for ligand targeting the CB1 and CB2 receptors. The optimal computational protocol explored in our earlier work was adopted for LRIP construction. This protocol, referred to as MIN + GB, combines energy minimization (MIN) and the Generalized Born (GB) model to account for solvent effects. It was chosen due to its low computational cost and superior performance compared to alternative simulation protocols and conventional docking algorithms. Full details of the system setup can be found in the original work. In this study, we adhered strictly to the previously established protocol except for one modification: the ABCG2 charges(116) was used instead of the AM1-BCC(*114, 115*) charges for ligand computations. Ligands with experimentally determined K_i or IC_{50} values for CB1 and CB2 receptors were obtained from the GPCRdb database (https://gpcrdb.org/). After processing, 580 ligands for CB1 and 1,357 ligands for CB2 were included in the training sets to construct the ML models.

Next, we generated ligand-receptor interaction profile (LRIP) for each ligand. We performed an energy decomposition analysis using the molecular mechanics-Generalized Born surface area (MM-GBSA) method. The optimized ligand-receptor complexes were used as input for MM-GBSA calculations, and binding free energies were decomposed into ligand-residue interactions for residues surrounding the ligand. An internal script was developed to extract ligand-residue interaction energies from the output of the Sander module in the AMBER 22 software package. Residues exhibiting interaction energies below -0.1 kcal/mol were identified as key interacting residues, contributing to the overall binding profile.

Then we trained prediction models using different ML algorithms, including ordinary least squares (LS) regression, Bayesian regression, support vector machine (SVR), random forest (RF), adaptive boosting (Adaboost), gradient boosting decision tree (GBDT) and multilayer perceptron (MLP). The performance of different ML algorithms evaluated using multiple metrics was summarized in **Tables S10-S11**.

Thermodynamics Integration Free Energy Calculations. Thermodynamic integration (TI) was employed to compute relative binding free energy differences between ligands, leveraging principles from statistical mechanics.

$$\Delta G_{A\rightarrow B} = \Delta G_{\lambda=1} - \Delta G_{\lambda=0} = \int_0^1 \langle \partial V/\partial \rangle_\lambda \, d\lambda$$

In TI, the free energy difference between two molecular states, "A" and "B," within a defined environment (e.g., a protein binding site or in solution), is evaluated by introducing a coupling parameter (λ). This parameter smoothly transitions the potential energy function between states. $\langle \partial V/\partial \lambda \rangle_{\lambda}$ denotes the ensemble average of the derivative of the potential energy with respect to λ, computed at each intermediate λ value. The free energy difference is computed by averaging $\partial V/\partial \lambda$ across multiple λ points, and the overall integral is approximated using trapezoidal numerical integration. The details of the methodology have been described extensively elsewhere(*51, 52*).

System preparation for all TI simulations was carried out using the FESetup tool (version 1.2.1)(*116*), which was modified to assign RESP charges to ligand molecules, by fitting electrostatic potentials calculated at the HF/6-31G* level using the Gaussian 16 package. Proteins, ligands, and water molecules were described using the ff14SB, GAFF2, and TIP3P force fields, respectively. Atom types and parameters for all ligand molecules were assigned using Antechamber and Parmchk2 from the AmberTools module. Each protein-ligand complex and free ligand system was solvated in a rectangular water box with a minimum buffer distance of 12 Å between the box edges and the nearest atoms of the ligands or complexes.

For compounds containing MCS ii features, the identified CHEMBL1209708 served as the reference state (state A) molecule, and then the other 8 selected ChEMBL and top 9 ZINC compounds served as state B molecules, following the thermodynamic cycle approach(*51, 52, 117*). The unique atoms in each ligand pair were treated with softcore potentials for both van der Waals and electrostatic interactions to ensure smooth transitions between states. Periodic boundary conditions and the NPT ensemble were employed throughout all simulations. The temperature was maintained at 298 K using Langevin dynamics with a collision frequency of 2.0 ps^{-1}, and the pressure was controlled at 1.01325 bar using the Monte Carlo barostat with a relaxation time of 2 ps. The SHAKE algorithm was disabled for all TI mutations, necessitating a time step of 1 fs in MD simulations. Energy data were recorded every 0.5 ps for subsequent analysis.

Two schedules of λ windows for TI simulations have been compared in this study, the simplest numerical integration (single-window - sw) and the Gaussian quadrature integration (full-window - fw), with the detailed λ values and the associated weights being presented in **Table S12**. For the sw-TI method, the free energy was evaluated by the integrand at the midpoint (λ = 0.5):

$$\Delta G_{A\to B} \cong \langle \partial V/\partial \lambda \rangle_{1/2}$$

While for the fw-TI, the formula for free energy calculation is as follows:

$$\Delta G_{A\to B} = \sum_i w_i \langle \partial V/\partial\lambda \rangle_i$$

Where $\partial V/\partial\lambda$ is the free energy change at each λ window and w_i is the associated weight. Initial equilibration for each system was performed using CPU-TI at $\lambda = 0.01592$ (or $\lambda = 0.5$ for sw-TI) for 100 ps, before switching to GPU-TI for production runs, as the CPU-TI algorithm allows larger fluctuation of box size under NPT ensemble simulations. For fw-TI, five evenly spaced snapshots were extracted from the final 100 ps of the CPU-TI equilibration and used as the starting configurations for five independent GPU-TI simulations at λ =0.01592. Each TI simulation ran for 5 ns, and the snapshot at the end of simulations was used as the starting points for subsequent λ windows, progressing toward the endpoints. Five independent replicas were run for each mutation pair, and relative binding free energies ($\Delta\Delta G_{sw}/\Delta\Delta G_{fw}$) were computed separately for each replica at all λ windows. The final binding free energy (($\Delta G_{sw}/\Delta G_{fw}$) for each ligand was obtained by averaging the results from the five replicas.

To calculate the relative binding free energy using TI, we need to identify a template for the target. For a given target molecule, either from ChEMBL or ZINC database, the template is a ChEMBL compound which shares at least 70% structure similarity and with K_i less than 10 µM. As to which exact template was selected for a given target, we used the rules of thumb in literature, such as the template is typically structurally smaller and highly potent(*53*).

Radioligand Binding Assays.

Radioligand binding assays were conducted for CB2 receptor to evaluate the binding affinity of the test compounds. For the promising compound, the same binding assay was also performed for CB1 receptor to evaluate their selectivity. The assays followed established protocols adapted from the scientific literature to ensure reliability and reproducibility (CB1(*118, 119*), CB2(*59*)). Reference standards were included in each assay to validate the results.

For the CB2 receptor assays, human recombinant cannabinoid CB2 receptor expressed in CHO-K1 cells (Eurofins Panlabs Discovery Services Taiwan) were employed. All test and reference compounds were dissolved in vehicle (1.0% DMSO), and the same vehicle concentration was used across all assay wells to control for solvent effects. The assays were conducted in modified HEPES buffer at pH 7.0 (20 mM HEPES, 0.5% BSA). Membrane aliquots (11.2 µg) were incubated with 2.4 nM [3H]WIN-55,212-2 (American Radiolabeled Chemicals) for 90 minutes at 37°C. The radioligand had a K_d of 4.90 nM, which was used together with IC_{50} values to calculate K_i via the Cheng–Prusoff equation(*120*). After incubation, membranes were filtered

and washed four times using 0.3% PEI-pretreated GF/C filters, and bound [3H]WIN-55,212-2 was measured. Non-specific binding was estimated in the presence of 10 μM R(+)-WIN-55,212-2. Test compounds were screened across various concentrations, and data were analyzed using non-linear regression with MathIQ™ (ID Business Solutions Ltd., UK).

As to the CB1 receptor assays, human recombinant cannabinoid CB1 receptors expressed in rat hematopoietic Chem-1 cells (Eurofins Discovery Services) were used. 1.0% DMSO was used as vehicle for all compounds, applied consistently across the concentration range. The assays were conducted in modified Tris-HCl buffer at pH 7.4 (50 mM HEPES, 5 mM MgCl2, 1 mM CaCl2, 0.2% BSA). Membrane aliquots (8 μg) were incubated with 2 nM [3H]SR141716A (Revvity/PerkinElmer) for 60 minutes at 37°C. The radioligand had a K_d of 18.0 nM, which was also used for K_i determination with the Cheng–Prusoff equation. After incubation, the membranes were filtered and washed four times using 0.3% PEI-pretreated GF/C filters, and the bound [3H]SR141716A was quantified. Non-specific binding was determined in the presence of 10 μM CP 55,940. Test compounds were screened across a range of concentrations, and the resulting data were analyzed using non-linear regression via MathIQ™ (ID Business Solutions Ltd., UK).

HTRF-based HER4 Inhibition Assay.

HTRF-based kinase inhibition assays were performed to evaluate the inhibitory activity of selected compounds against human HER4 kinase. The assays were conducted using recombinant HER4 protein (Invitrogen, PR6308B) in a homogeneous time-resolved fluorescence (HTRF) format, following the manufacturer's recommended protocol to ensure reliability. Afatinib, a clinically approved irreversible HER family inhibitor, was included as a reference compound to validate assay performance and reproducibility.

HER4 kinase (0.5 nM) was incubated with a biotinylated tyrosine kinase (TK) peptide substrate (1 μM) in assay buffer containing 50 mM HEPES, 10 mM $MgCl_2$, 1 mM EDTA, 0.01% Brij-35, and 1 mM DTT. Test compounds were prepared in DMSO and added at the indicated concentrations, with constant DMSO content maintained across all assay wells. After compound addition, plates were incubated for 15 minutes at 23 °C. The kinase reaction was initiated by the addition of ATP at its apparent K_m concentration (10 μM) and incubated for 90 minutes at 23 °C. Subsequently, a 2× detection mixture was added to each well, followed by incubation for 60 minutes at 23 °C. Plates were then read on a PerkinElmer EnVision plate reader.

Fluorescence emissions at 665 nm and 615 nm were recorded, and the TR-FRET ratio (665/615) was calculated automatically by the instrument software. The TR-FRET ratios were normalized to internal controls to calculate percentage inhibition, with wells containing peptide only serving as 100% inhibition controls and wells containing enzyme and peptide without inhibitor defining 0% inhibition. Dose–response curves were generated by plotting percentage inhibition against the logarithm of compound concentration and fitted using a four-parameter logistic model. IC_{50} values were determined using XLfit software.

Supplemental Figures

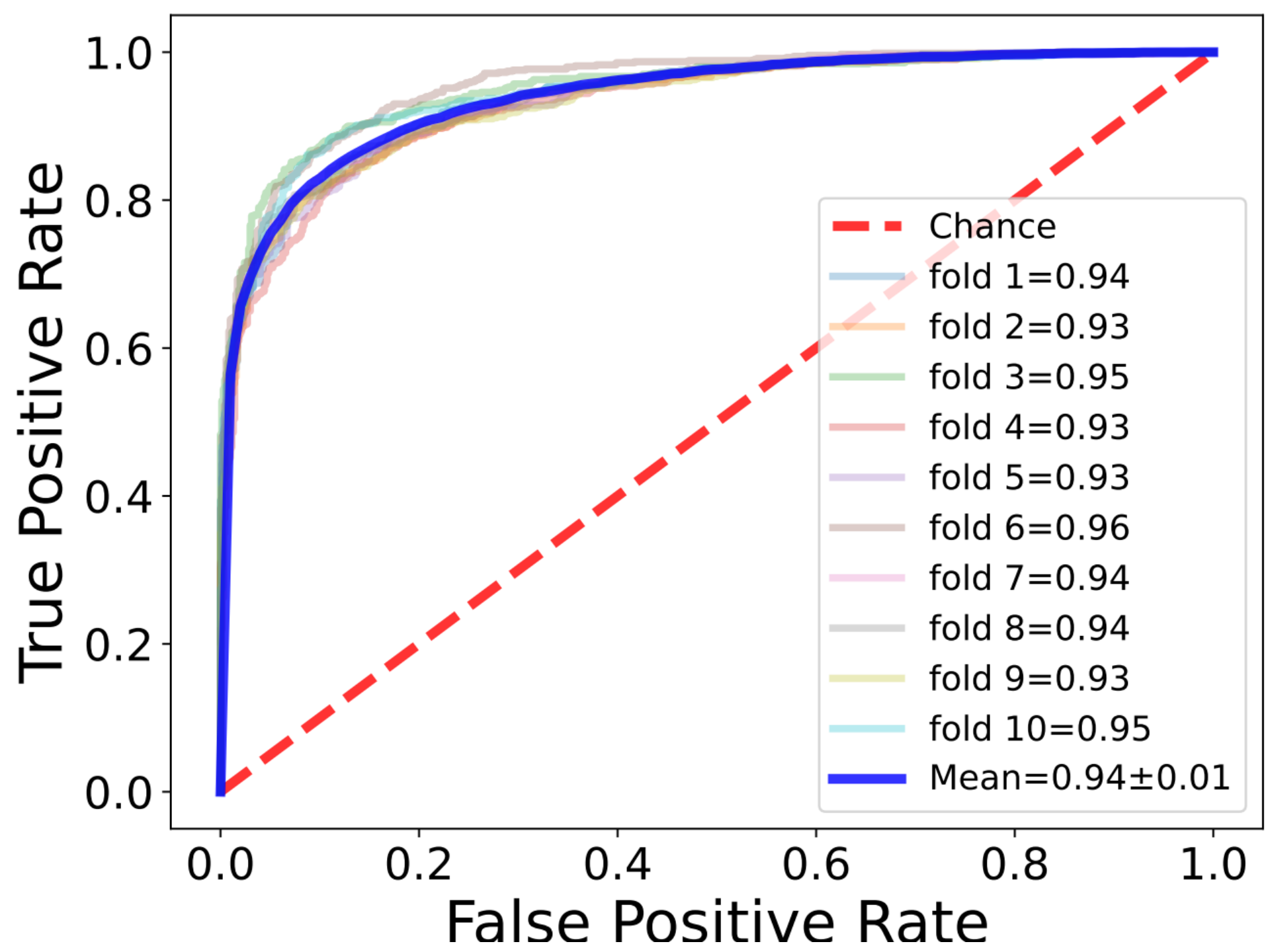

Fig. S1 ROC curves the discriminator model with MLP architecture.

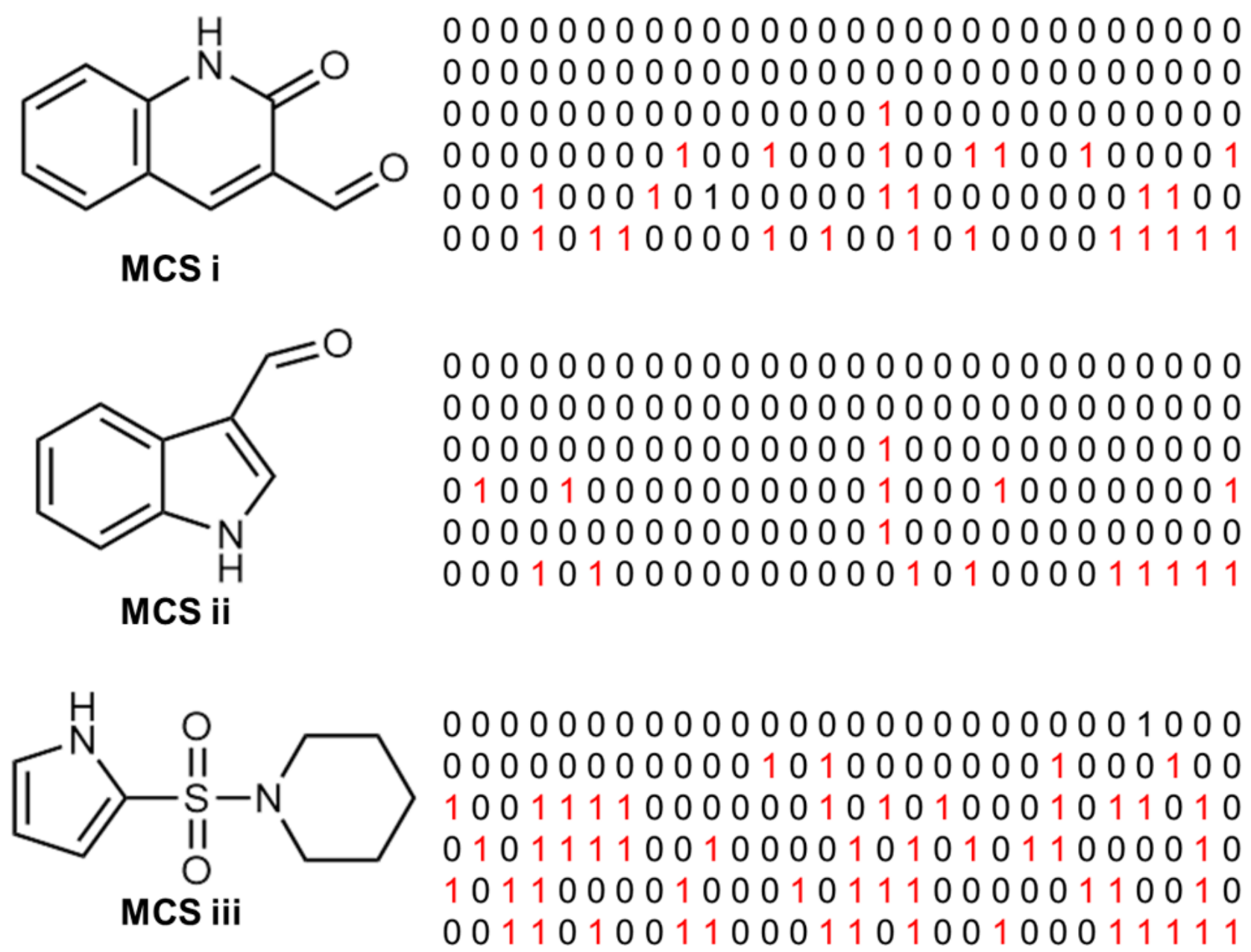

Fig. S2 Top three MCSs and MACCS for CB2 compounds.

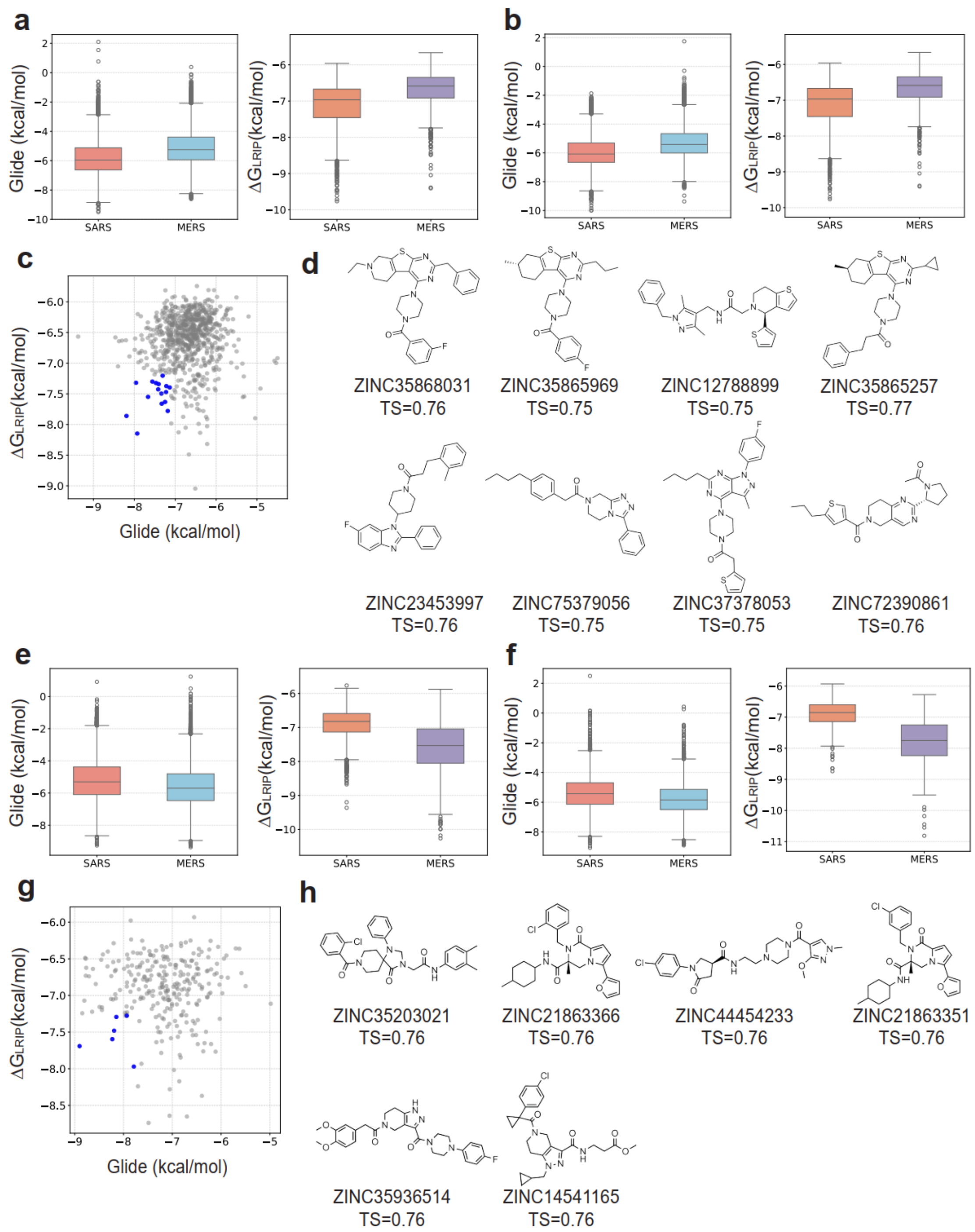

Fig. S3. Application of FSGLD pipeline in Scenario II for SARS and MERS targets. a-h. Extension to SARS-CoV-2 Mpro (SARS). e-h. Extension to MERS-CoV Mpro (MERS). a/e. Selectivity prediction of Glide docking and LRIP energy prediction for ChEMBL compounds targeting SARS and MERS. b/f. Selectivity prediction of Glide docking and LRIP energy prediction for ZINC compounds targeting SARS and MERS. c/g. The scatter plot of ΔG_{LRIP} versus Glide docking score for filtering ZINC candidates (blue dots). d/h. Top ZINC candidates selected based on Glide docking score and LRIP binding free energy prediction.

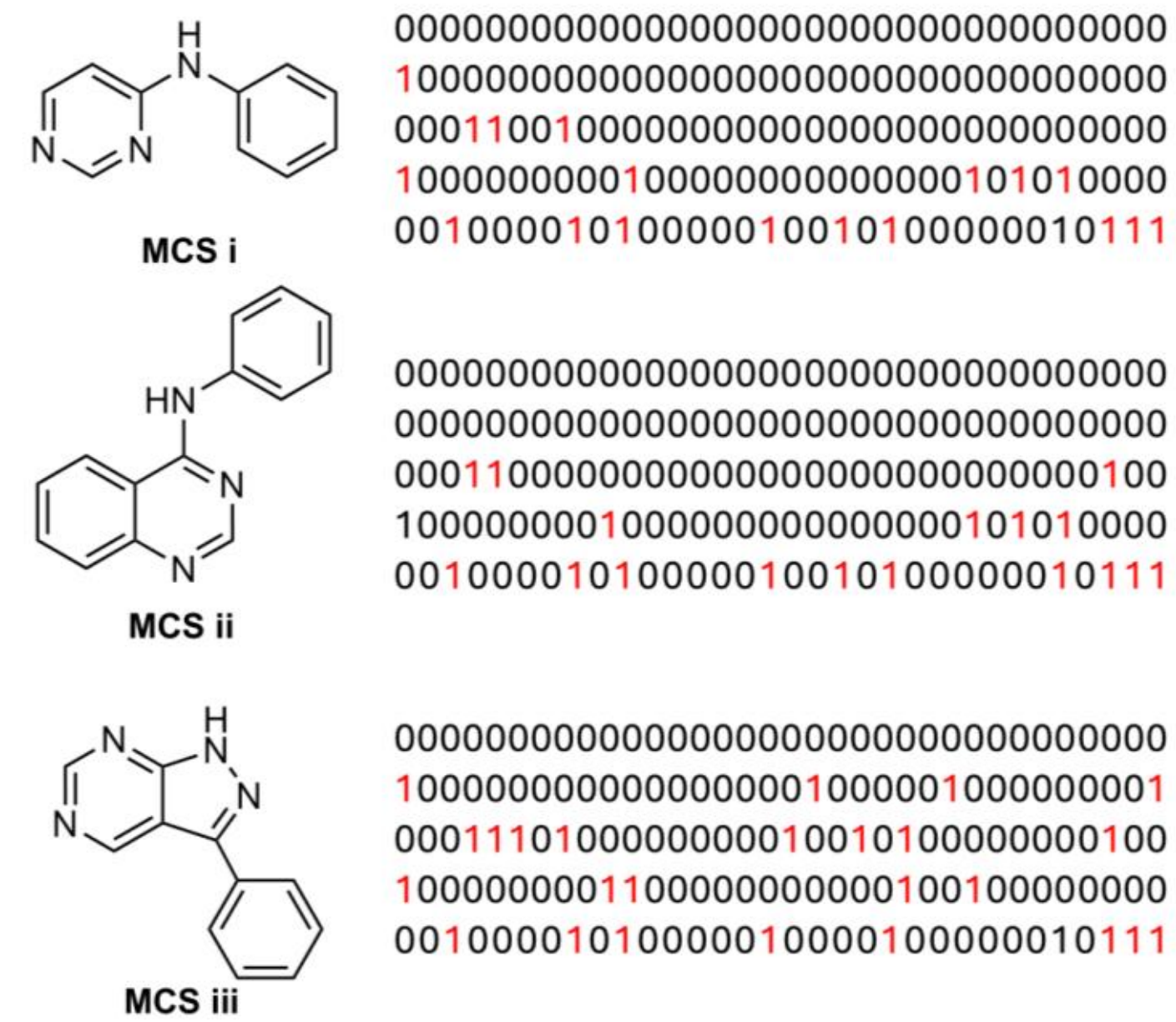

Fig. S4 Top three MCSs and MACCS for HER4 compounds.

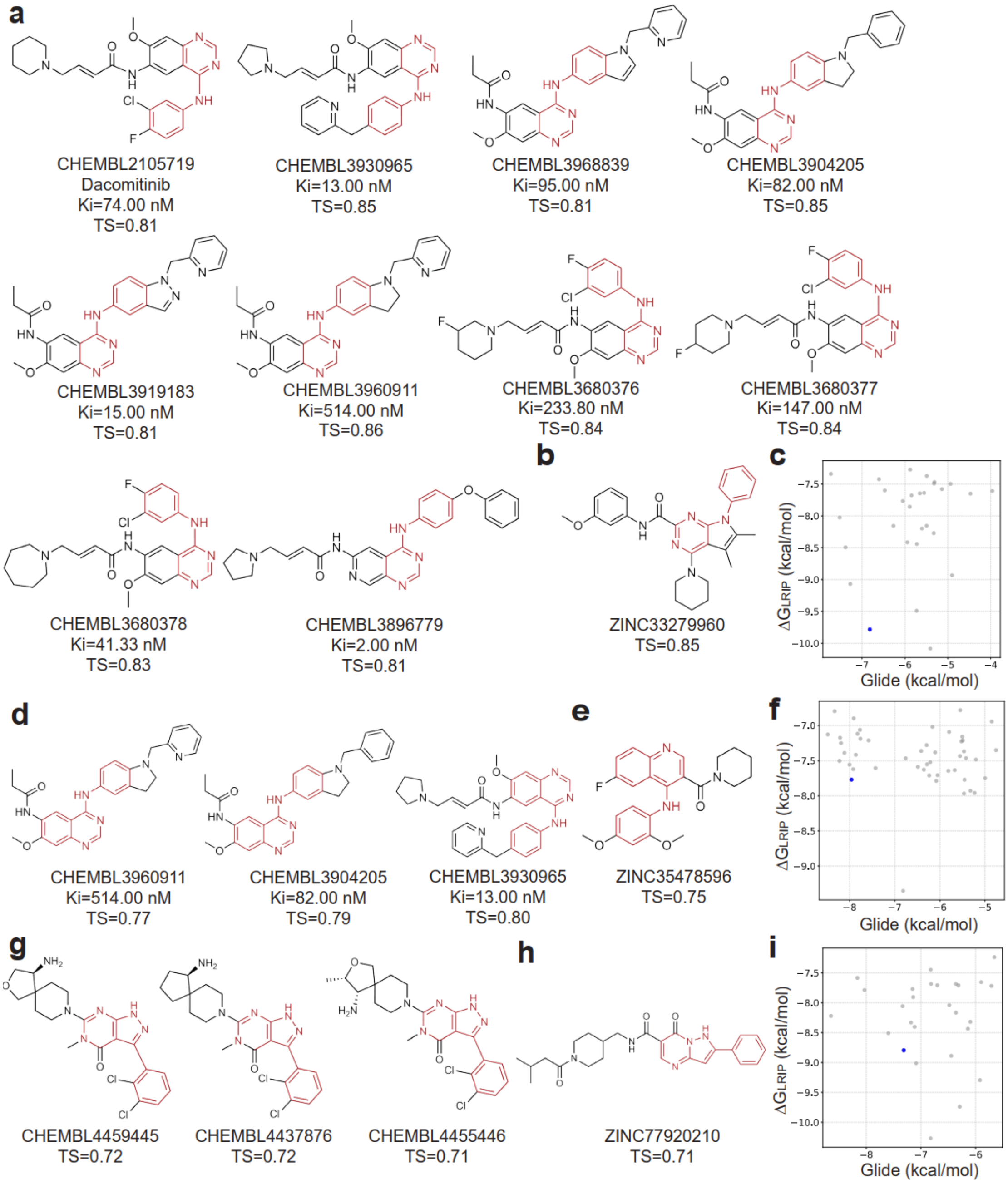

Fig. S5 Application of FSGLD pipeline in Scenario III for HER4 target. a-c. for MCS i. d-f. for MCS ii. g-i. for MCS iii. a/d/g. The top Compounds containing the specific MCS substructure by searching the ChEMBL database. b/e/h. The final ZINC compound hits obtained after applying a stringent two-stage filtering screening strategy. c/f/i. The scatter plot of $\Delta \boldsymbol{G}_{\boldsymbol{LRIP}}$ versus Glide docking score for filtering ZINC candidates (blue dots).

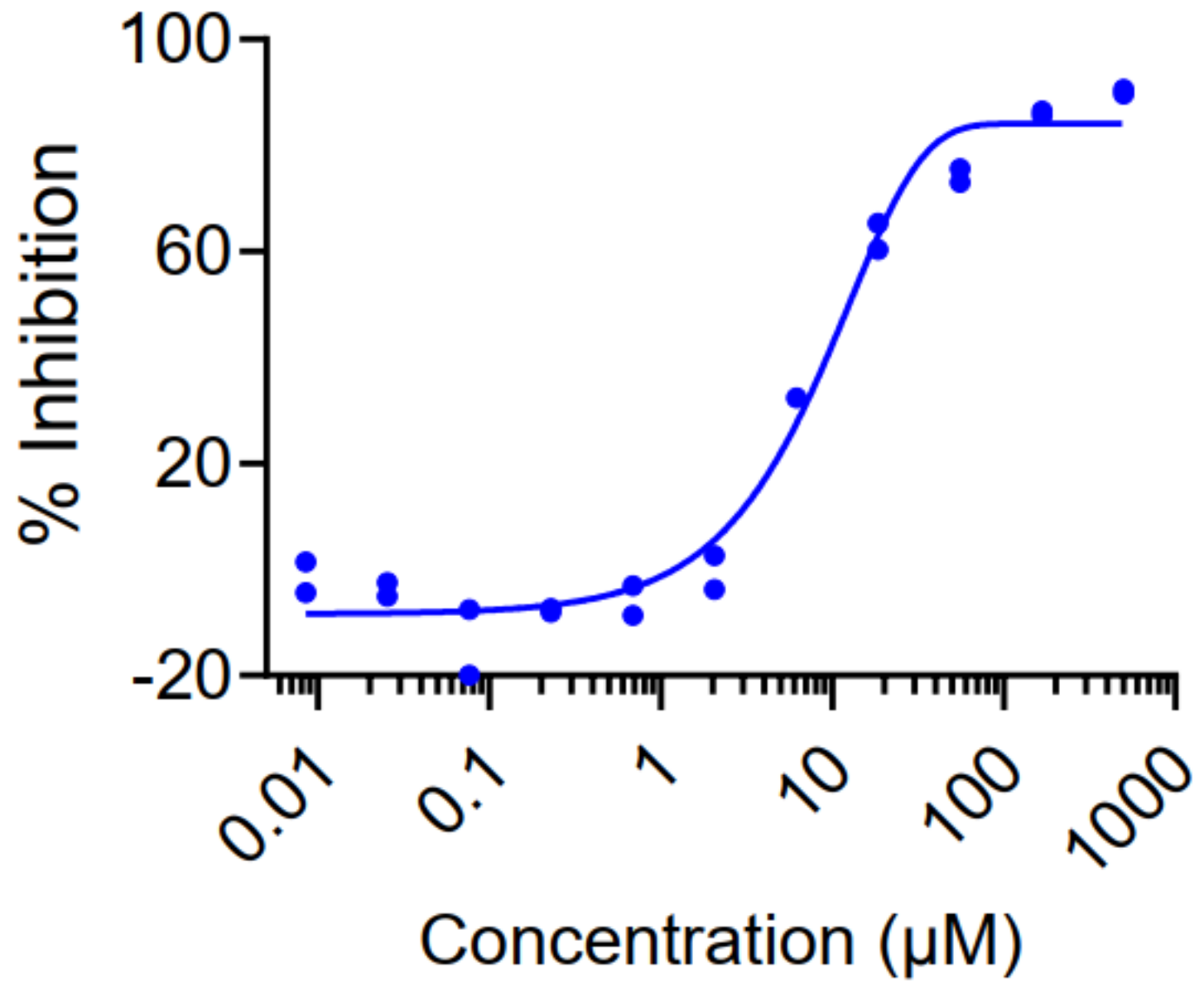

Fig. S6 Concentration-response curves of ZINC33279960 in HTRF kinase inhibition assays of HER4.

Supplemental Tables

Table S2. The performance of the discriminator in dcGAN trained using different architectures. For a given performance metrix, the value is the average of 10-fold cross validation.

Neural Network	AUC	Accuracy	F1-score	Precision	Recall
LeNet 5	0.88	0.81	0.81	0.82	0.79
AlexNet	0.94	0.87	0.87	0.88	0.85
ZFNet	0.86	0.78	0.78	0.80	0.75
VGGNet11	0.65	0.53	0.66	0.52	0.91
VGGNet13	0.68	0.62	0.65	0.62	0.71
VGGNet16	0.65	0.58	0.64	0.57	0.75
MLP	0.94	0.86	0.86	0.90	0.81

Table S3. Selected top ZINC compounds according to the QED druglike metrics.

Molecule	MW	logP	TPSA	QED	TS
ZINC63855506	303.431	2.828	48.990	0.948	0.708
ZINC71836518	302.374	2.818	54.560	0.948	0.702
ZINC19852625	301.415	2.757	45.230	0.948	0.700
ZINC19117964	301.365	2.597	48.990	0.948	0.705
ZINC89661821	307.806	3.037	45.230	0.948	0.708
ZINC47073394	313.401	2.838	58.220	0.948	0.702
ZINC89814317	310.401	3.072	50.160	0.948	0.710
ZINC92589586	304.781	3.116	50.160	0.948	0.700
ZINC77303792	313.376	2.898	59.220	0.948	0.704
ZINC71970254	298.390	2.971	50.160	0.947	0.702
ZINC12544729	298.390	2.606	59.810	0.947	0.702
ZINC55928065	297.402	3.078	48.990	0.947	0.710

Table S4. Selected top ZINC compounds according to the structural similarity score.

Molecule	MW	logP	TPSA	QED	TS
ZINC01081597	295.299	-1.748	105.380	0.696	0.848
ZINC37449194	363.805	0.554	96.150	0.690	0.839
ZINC02437843	363.805	0.919	104.940	0.565	0.833
ZINC92191757	401.467	1.004	103.310	0.584	0.823
ZINC10385107	407.858	0.938	103.310	0.565	0.822

ZINC28869273	385.448	-1.508	100.590	0.507	0.822
ZINC12324327	405.458	3.313	104.290	0.510	0.822
ZINC87360737	387.440	0.695	103.310	0.590	0.821
ZINC90100884	387.440	0.695	103.310	0.590	0.821
ZINC88510547	389.459	3.613	95.060	0.544	0.821
ZINC20509006	363.805	0.919	104.940	0.565	0.821
ZINC09126041	401.467	1.895	103.170	0.568	0.819

Table S5. The docking and similarity scores of selected top CB2 active candidates from ZINC database. Docking scores are in kcal/mol**.**

Molecule	CB2 Docking Score	CB1 Docking Score	Tanimoto Score
ZINC38533996	-12.97	-9.54	0.85
ZINC38534068	-12.90	-9.37	0.86
ZINC12163344	-12.62	-8.13	0.88
ZINC14882156	-12.22	-9.37	0.85
ZINC77321744	-12.00	-10.60	0.85
ZINC72402087	-11.98	-10.32	0.86
ZINC38534026	-11.90	-9.43	0.85
ZINC11968663	-11.70	-10.10	0.85
ZINC09125498	-11.60	-10.31	0.87
ZINC12242275	-11.59	-7.51	0.87
ZINC06756714	-11.54	-10.04	0.86
ZINC29419331	-11.47	-10.80	0.85

Table S6. The docking and similarity scores of selected top CB2 active candidates with MCS i feature from ZINC database. Docking scores are in kcal/mol**.**

Molecule	CB2 Docking score	CB1 Docking score	TS
ZINC72469974	-10.08	-8.00	0.82
ZINC91152990	-7.82	-6.75	0.77
ZINC69537317	-8.93	-9.30	0.77
ZINC71836805	-9.39	-9.19	0.77
ZINC71836806	-9.32	-9.45	0.77
ZINC77505253	-10.56	-9.86	0.77
ZINC77505252	-10.24	-9.44	0.77
ZINC71747425	-9.36	-9.369	0.77
ZINC92076350	-10.56	-9.55	0.77

ZINC69489680	-10.11	-9.07	0.77
ZINC71752134	-9.35	-9.82	0.77
ZINC71752133	-10.00	-10.03	0.77

Table S7 The Glide docking scores and similarity scores of selected top CB2 active candidates containing MCS ii substructure from the ZINC database. Docking scores are in kcal/mol.

Molecule	CB2 Docking score	CB1 Docking score	TS
ZINC24299219	-11.03	-10.04	0.82
ZINC34847913	-9.72	-9.49	0.82
ZINC49413728	-8.86	-4.78	0.82
ZINC04788921	-9.83	-9.87	0.81
ZINC74199659	-9.14	-9.83	0.81
ZINC72403380	-10.18	-7.12	0.81
ZINC72403381	-10.55	-9.91	0.81
ZINC77179032	-10.34	-10.34	0.81
ZINC04788914	-10.24	NA	0.81

Table S8. The experimental and predicted binding free energies of a set of ChEMBL compounds targeting to the CB2 receptor. The docking scores and free energies are in kcal/mol.

Compound	ΔG_{exp}	Glide	ΔG_{LRIP}	$\Delta G_{MM-PBSA}$	$\Delta\Delta G_{sw}$	$\Delta\Delta G_{fw}$	ΔG_{sw}	ΔG_{fw}
CHEMBL1209708	-12.38	-10.38	-10.81	-21.61	Ref	Ref	Ref	Ref
CHEMBL310431	-10.41	-10.72	-10.09	-18.83	1.92	-0.04	-10.46	-12.42
CHEMBL80439	-10.23	-10.61	-9.76	-21.06	1.24	1.42	-11.14	-10.96
CHEMBL431363	-10.25	-10.69	-10.43	-20.75	0.41	4.63	-11.96	-7.74
CHEMBL432750	-10.05	-10.94	-10.21	-19.95	3.92	-2.12	-8.46	-14.5
CHEMBL2112291	-9.82	-9.83	-9.4	-15.43	2.61	3.28	-9.77	-9.1
CHEMBL418916	-10.09	-9.95	-9.59	-19.07	-1.21	1.13	-13.59	-11.24
CHEMBL312093	-8.98	-9.5	-9.99	-10.97	3.44	3.9	-8.94	-8.48
CHEMBL310766	-8.95	-11.36	-9.66	-17.17	6.37	6.77	-6.01	-5.6

Table S9. The predicted binding energies of a set of ZINC compounds targeting to CB2 using different computational methods. All docking scores and free energies are in kcal/mol.

**Compound**	**Glide**	ΔG_{LRIP}	$\Delta G_{MM-PBSA}$	ΔG_{sw}
ZINC04788914	-10.24	-11.27	-11.19	-2.92
ZINC04788921	-9.83	-9.67	-10.23	-6.46

ZINC24299219	-11.03	-10.51	-10.21	-7.69
ZINC34847913	-9.72	-11.03	-5.25	-5.22
ZINC49413728	-8.86	-9.1	-11.56	-6.12
ZINC72403380	-10.18	-9.94	-9.52	2.17
ZINC72403381	-10.55	-10.5	-11.37	5.43
ZINC74199659	-9.14	-10.37	-1.22	-4.48
ZINC77179032	-10.34	-9.46	-11.95	-8.23

Table S10. The performance metrics of ML-based SFs for CB1

	RMSE	MAE	CORR	CORR2	PI
LS	1.38	1.07	0.46	0.21	0.51
Bayesian	1.23	0.98	0.52	0.27	0.54
SVR	1.24	0.99	0.50	0.25	0.50
RF	1.09	0.87	0.67	0.44	0.66
Adaboost	1.07	0.86	0.66	0.44	0.68
GBDT	1.06	0.84	0.67	0.45	0.67
MLP	1.36	1.10	0.51	0.26	0.50

Table S11. The performance metrics of ML-based SFs for CB2

	RMSE	MAE	CORR	CORR2	PI
LS	1.07	0.88	0.41	0.17	0.40
Bayesian	1.10	0.93	0.32	0.11	0.33
SVR	1.07	0.90	0.39	0.15	0.39
RF	1.03	0.88	0.50	0.25	0.50
Adaboost	1.11	0.93	0.37	0.14	0.39
GBDT	0.97	0.81	0.56	0.31	0.55
MLP	1.19	0.94	0.35	0.12	0.36

Table S12. Schedules of λ windows for TI in this study

No. of λ	λ_i	w_i	Integration method
1	0.5	-	Single-Window
9	0.01592, 0.08198, 0.19331, 0.33787, 0.5, 0.66213, 0.80669, 0.91802, 0.98408	0.04064, 0.09032, 0.13031, 0.15617, 0.16512, 0.15617, 0.13031, 0.09032, 0.04064	Full-Window (Gaussian quadrature)

Table S13. The numbers of identified SARS compounds from ChEMBL and ZINC databases using different structural similarity criteria.

Database	TS ≥ 0.70	TS ≥ 0.75	TS ≥ 0.80	TS ≥ 0.85
ChEMBL	1444	81	0	0
ChEMBL (100 ≤ MW ≤ 500)	1316	71	0	0
ZINC	19273	1050	1	0

Table S14. The predicted binding affinity of a set of ZINC compounds targeting SARS using Glide docking and LRIP. All docking scores and free energies are in kcal/mol.

**Compound**	**Glide**	ΔG_{LRIP}
ZINC35868031	-8.19	-7.86
ZINC35865969	-7.96	-7.32
ZINC12788899	-7.93	-8.15
ZINC35865257	-7.66	-7.55
ZINC23453997	-7.56	-7.30
ZINC75379056	-7.47	-7.32
ZINC37378053	-7.42	-7.43
ZINC72390861	-7.41	-7.34

Table S15. The numbers of identified MERS compounds from ChEMBL and ZINC databases using different structural similarity criteria.

Database	TS ≥ 0.70	TS ≥ 0.75	TS ≥ 0.80	TS ≥0.85
ChEMBL	6381	220	0	0
ChEMBL (100 ≤ MW ≤ 500)	1774	24	0	0
ZINC	11824	321	0	0

Table S16. The predicted binding affinity of a set of ZINC compounds targeting MERS using Glide docking and LRIP. All docking scores and free energies are in kcal/mol.

**Compound**	**Glide**	ΔG_{LRIP}

ZINC35203021	-8.90	-7.69
ZINC21863366	-8.23	-7.60
ZINC44454233	-8.19	-7.48
ZINC21863351	-8.15	-7.29
ZINC35936514	-7.93	-7.28
ZINC14541165	-7.79	-7.97

Table S17. Summary of substructure and similarity search for HER4 compounds using MACCS fingerprints in Scenario III.

MCS Feature	Database and Subset	TS≥0.70	TS≥0.75	TS≥0.80	TS≥0.85
MCS i	ChEMBL	68592	26109	4981	192
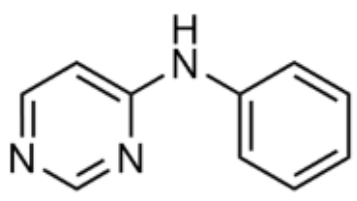	ChEMBL (MW: 100-500)	44989	15685	2740	110
MCS i	ZINC	269817	94683	16325	522
MCS ii	ChEMBL	24759	3745	154	0
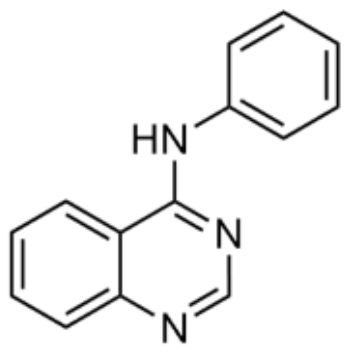	ChEMBL (MW: 100-500)	11833	1314	60	0
MCS ii	ZINC	54044	4166	26	0
MCS iii	ChEMBL	397	30	0	0
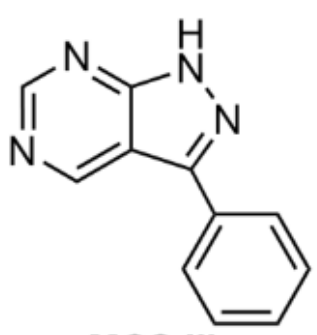	ChEMBL (MW: 100-500)	236	16	0	0
MCS iii	ZINC	880	19	0	0

Table S18. Binding energies of final ZINC compound hits targeting HER4 predicted using Glide docking and the LRIP model. All docking scores and LRIP binding free energies are in kcal/mol.

MCS	Compound	Glide	ΔG_{LRIP}
MCS i	ZINC33279960	-6.82	-9.78
MCS ii	ZINC35478596	-7.96	-7.77
MCS iii	ZINC77920210	-7.31	-8.79

REFERENCES

1. J. A. DiMasi, H. G. Grabowski, R. W. Hansen, Innovation in the pharmaceutical industry: New estimates of R&D costs. *J. Health Econ.* **47**, 20–33 (2016).

2. O. J. Wouters, M. McKee, J. Luyten, Estimated research and development investment needed to bring a new medicine to market, 2009-2018. *JAMA* **323**, 844–853 (2020).

3. P. Polishchuk, T. I. Madzhidov, A. Varnek, Estimation of the size of drug-like chemical space based on GDB-17 data. *J. Comput. Aided Mol. Des.* **27**, 675–679 (2013).

4. J. Hert, J. J. Irwin, C. Laggner, M. J. Keiser, B. K. Shoichet, Quantifying biogenic bias in screening libraries. *Nat. Chem. Biol.* **5**, 479–483 (2009).

5. L. G. Ferreira, R. N. Dos Santos, G. Oliva, A. D. Andricopulo, Molecular docking and structure-based drug design strategies. *Molecules* **20**, 13384–13421 (2015).

6. M. H. S. Segler, T. Kogej, C. Tyrchan, M. P. Waller, Generating focused molecule libraries for drug discovery with recurrent neural networks. *ACS Cent. Sci.* **4**, 120–131 (2018).

7. A. Gupta, A. T. Müller, B. J. H. Huisman, J. A. Fuchs, P. Schneider, G. Schneider, Erratum: Generative recurrent networks for de novo drug design. *Mol. Inform.* **37**, 1880141 (2018).

8. H. Dai, Y. Tian, B. Dai, S. Skiena, L. Song, Syntax-directed variational autoencoder for structured data. arXiv:1802.08786 [cs.LG] (2018).

9. R. Gómez-Bombarelli, J. N. Wei, D. Duvenaud, J. M. Hernández-Lobato, B. Sánchez-Lengeling, D. Sheberla, J. Aguilera-Iparraguirre, T. D. Hirzel, R. P. Adams, A. Aspuru-Guzik, Automatic chemical design using a data-driven continuous representation of molecules. *ACS Cent. Sci.* **4**, 268–276 (2018).

10. G. L. Guimaraes, B. Sanchez-Lengeling, C. Outeiral, P. L. C. Farias, A. Aspuru-Guzik, Objective-Reinforced Generative Adversarial Networks (ORGAN) for sequence generation models. arXiv:1705.10843 [stat.ML] (2018).

11. M. J. Kusner, B. Paige, J. M. Hernández-Lobato, Grammar variational autoencoder. arXiv:1703.01925 [stat.ML] (2017).

12. B. Samanta, A. De, G. Jana, P. K. Chattaraj, N. Ganguly, M. Gomez-Rodriguez, NeVAE: A deep generative model for molecular graphs. arXiv:1802.05283 [cs.LG] (2019).

13. W. Jin, R. Barzilay, T. Jaakkola, Junction tree variational autoencoder for molecular graph generation. arXiv:1802.04364 [cs.LG] (2019).

14. N. De Cao, T. Kipf, MolGAN: An implicit generative model for small molecular graphs. arXiv:1805.11973 [stat.ML] (2018).

15. M. Simonovsky, N. Komodakis, GraphVAE: Towards generation of small graphs using variational autoencoders. arXiv: 1802.03480 [cs.LG] (2018).

16. J. You, B. Liu, R. Ying, V. Pande, J. Leskovec, Graph convolutional policy network for goal-directed molecular graph generation. arXiv:1806.02473 [cs.LG] (2019).

17. H. Zhang, Y. Liu, X. Liu, C. Wang, M. Guo, Equivariant score-based generative diffusion framework for 3D molecules. *BMC Bioinformatics* **25**, 203 (2024).

18. A. Kadurin, A. Aliper, A. Kazennov, P. Mamoshina, Q. Vanhaelen, K. Khrabrov, A. Zhavoronkov, The cornucopia of meaningful leads: Applying deep adversarial autoencoders for new molecule development in oncology. *Oncotarget* **8**, 10883–10890 (2017).

19. Y. Bian, J. Wang, J. J. Jun, X.-Q. Xie, Deep convolutional generative adversarial network (dcGAN) models for screening and design of small molecules targeting cannabinoid receptors. *Mol. Pharm.* **16**, 4451–4460 (2019).

20. V. G. Satorras, E. Hoogeboom, F. Fuchs, I. Posner, M. Welling, E (n) equivariant normalizing flows. *Adv. Neural Inf. Process. Syst.* **34**, 4181–4192 (2021).

21. E. Hoogeboom, V. G. Satorras, C. Vignac, M. Welling, "Equivariant diffusion for molecule generation in 3D," in *International conference on machine learning* (PMLR, 2022), pp. 8867–8887.

22. V. G. Satorras, E. Hoogeboom, M. Welling, "E(n) equivariant graph neural networks," in *International conference on machine learning* (PMLR, 2021), pp. 9323–9332.

23. I. J. Goodfellow, J. Pouget-Abadie, M. Mirza, B. Xu, D. Warde-Farley, S. Ozair, A. Courville, Y. Bengio, "Generative Adversarial Nets," in *Advances in Neural Information Processing Systems*, K. Q. Weinberger, Ed. (Curran Associates Inc., 2014), vol. 27.

24. E. Denton, S. Chintala, A. Szlam, R. Fergus, "Deep generative image models using a Laplacian pyramid of adversarial networks," in *Proceedings of the 28th International Conference on Neural Information Processing Systems* (Curran Associates lnc., 2015), vol 1.

25. A. Radford, L. Metz, S. Chintala, Unsupervised representation learning with deep convolutional generative adversarial networks. arXiv:1511.06434 [cs.LG] (2016).

26. O. Prykhodko, S. V. Johansson, P.-C. Kotsias, J. Arús-Pous, E. J. Bjerrum, O. Engkvist, H. Chen, A de novo molecular generation method using latent vector based generative adversarial network. *J. Cheminform.* **11**, 74 (2019).

27.. Maziarka, A. Pocha, J. Kaczmarczyk, K. Rataj, T. Danel, M. Warchoł, Mol-CycleGAN: A generative model for molecular optimization. *J. Cheminform.* **12**, 2 (2020).

28. O. Méndez-Lucio, B. Baillif, D.-A. Clevert, D. Rouquié, J. Wichard, De novo generation of hit-like molecules from gene expression signatures using artificial intelligence. *Nat. Commun.* **11**, 10 (2020).

29. M. Abbasi, B. P. Santos, T. C. Pereira, R. Sofia, N. R. C. Monteiro, C. J. V. Simões, R. M. M. Brito, B. Ribeiro, J. L. Oliveira, J. P. Arrais, Designing optimized drug candidates with Generative Adversarial Network. *J. Cheminform.* **14**, 40 (2022).

30. J. C. Ashton, D. Friberg, C. L. Darlington, P. F. Smith, Expression of the cannabinoid CB2 receptor in the rat cerebellum: An immunohistochemical study. *Neurosci. Lett.* **396**, 113–116 (2006).

31. J.-P. Gong, E. S. Onaivi, H. Ishiguro, Q.-R. Liu, P. A. Tagliaferro, A. Brusco, G. R. Uhl, Cannabinoid CB2 receptors: Immunohistochemical localization in rat brain. *Brain Res.* **1071**, 10–23 (2006).

32. T. Tuccinardi, P. L. Ferrarini, C. Manera, G. Ortore, G. Saccomanni, A. Martinelli, Cannabinoid CB2/CB1 selectivity. Receptor modeling and automated docking analysis. *J. Med. Chem.* **49**, 984–994 (2006).

33. P. Pacher, R. Mechoulam, Is lipid signaling through cannabinoid 2 receptors part of a protective system? *Prog. Lipid Res.* **50**, 193–211 (2011).

34. Y. LeCun, Y. Bengio, G. Hinton, Deep learning. *Nature* **521**, 436–444 (2015).

35. A. Krizhevsky, I. Sutskever, G. E. Hinton, "ImageNet classification with deep convolutional neural networks," in *Proceedings of the 25th International Conference on Neural Information Processing Systems* (Curran Associates lnc., 2012), vol 1.

36. M. D. Zeiler, R. Fergus, "Visualizing and understanding convolutional networks," in *Computer Vision – ECCV 2014*, D. Fleet, T. Pajdla, B. Schiele, T. Tuytelaars, Eds. (Springer International Publishing, 2014), pp. 818–833.

37. K. Simonyan, A. Zisserman, Very deep convolutional networks for large-scale image recognition. arXiv:1409.1556 [cs.CV] (2015).

38. B. Zdrazil, E. Felix, F. Hunter, E. J. Manners, J. Blackshaw, S. Corbett, M. de Veij, H. Ioannidis, D. M. Lopez, J. F. Mosquera, M. P. Magarinos, N. Bosc, R. Arcila, T. Kizilören, A. Gaulton, A. P. Bento, M. F. Adasme, P. Monecke, G. A. Landrum, A. R. Leach, The ChEMBL Database in 2023: A drug discovery platform spanning multiple bioactivity data types and time periods. *Nucleic Acids Res.* **52**, D1180–D1192 (2024).

39. M. Mirza, S. Osindero, Conditional generative adversarial nets. arXiv:1411.1784 [cs.LG] (2014).

40. I. B. Mustapha, S. Hasan, H. Nabus, S. M. Shamsuddin, Conditional deep convolutional generative adversarial networks for isolated handwritten arabic character generation. *Arab. J. Sci. Eng.* **47**, 1309–1320 (2022).

41. S. So, J. Rho, Designing nanophotonic structures using conditional deep convolutional generative adversarial networks. *Nanophotonics* **8**, 1255–1261 (2018).

42. Z. Zhong, A. Y. Sun, H. Jeong, Predicting CO_2 plume migration in heterogeneous formations using conditional deep convolutional generative adversarial network. *Water Resour. Res.* **55**, 5830–5851 (2019).

43. J. Luo, J. Huang, H. Li, A case study of conditional deep convolutional generative adversarial networks in machine fault diagnosis. *J. Intell. Manuf.* **32**, 407–425 (2021).

44. B. Ji, X. He, J. Zhai, Y. Zhang, V. H. Man, J. Wang, Machine learning on ligand-residue interaction profiles to significantly improve binding affinity prediction. *Brief. Bioinform.* **22**, bbab054 (2021).

45. T. Hou, J. Wang, Y. Li, W. Wang, Assessing the performance of the MM/PBSA and MM/GBSA methods. 1. The accuracy of binding free energy calculations based on molecular dynamics simulations. *J. Chem. Inf. Model.* **51**, 69–82 (2011).

46. W. Rocchia, E. Alexov, B. Honig, Extending the applicability of the nonlinear Poisson−Boltzmann equation: Multiple dielectric constants and multivalent ions. *J. Phys. Chem. B* **105**, 6507–6514 (2001).

47. B. Kuhn, P. Gerber, T. Schulz-Gasch, M. Stahl, Validation and use of the MM-PBSA approach for drug discovery. *J. Med. Chem.* **48**, 4040–4048 (2005).

48. J. M. J. Swanson, R. H. Henchman, J. A. McCammon, Revisiting free energy calculations: A theoretical connection to MM/PBSA and direct calculation of the association free energy. *Biophys. J.* **86**, 67–74 (2004).

49. E. Wang, H. Sun, J. Wang, Z. Wang, H. Liu, J. Z. H. Zhang, T. Hou, End- free energy calculation with MM/PBSA and MM/GBSA: Strategies and applications in drug design. *Chem. Rev.* **119**, 9478–9508 (2019).

50. W. Junmei, H. Tingjun, X. Xiaojie, Recent advances in free energy calculations with a combination of molecular mechanics and continuum models. *Curr. Comput. Aided Drug Des.* **2**, 287–306 (2006).

51. D. A. Pearlman, P. S. Charifson, Are free energy calculations useful in Practice? A Comparison with rapid scoring functions for the p38 MAP kinase protein system. *J. Med. Chem.* **44**, 3417–3423 (2001).

52. J. Michel, M. L. Verdonk, J. W. Essex, Protein-ligand binding affinity predictions by implicit solvent simulations: A tool for lead optimization? *J. Med. Chem.* **49**, 7427–7439 (2006).

53. X. He, S. Liu, T.-S. Lee, B. Ji, V. H. Man, D. M. York, J. Wang, Fast, Accurate, and reliable protocols for routine calculations of protein-ligand binding affinities in drug design projects using AMBER GPU-TI with ff14SB/GAFF. *ACS Omega* **5**, 4611–4619 (2020).

54. M. Korablyov, C.-H. Liu, M. Jain, A. M. van der Sloot, E. Jolicoeur, E. Ruediger, A. C. Nica, E. Bengio, K. Lapchevskyi, D. St-Cyr, D. A. Schuetz, V. I. Butoi, J. Rector-Brooks, S. Blackburn, L. Feng, H. Nekoei, S. Gottipati, P. Vijayan, P. Gupta, L. Rampášek, S. Avancha, P.-L. Bacon, W. L. Hamilton, B. Paige, S. Misra, S. K. Jastrzebski, B. Kaul, D. Precup, J. M. Hernández-Lobato, M. Segler, M. Bronstein, A. Marinier, M. Tyers, Y. Bengio, Generative active learning for the search of small-molecule protein binders. arXiv:2405.01616 [q-bio.BM] (2024).

55. V. Chenthamarakshan, S. C. Hoffman, C. D. Owen, P. Lukacik, C. Strain-Damerell, D. Fearon, T. R. Malla, A. Tumber, C. J. Schofield, H. M. Duyvesteyn, W. Dejnirattisai, L. Carrique, T. S. Walter, G. R. Screaton, T. Matviiuk, A. Mojsilovic, J. Crain, M. A. Walsh, D. I. Stuart, P. Das, Accelerating drug target inhibitor discovery with a deep generative foundation model. *Sci. Adv.* **9**, eadg7865 (2023).

56. V. Law, C. Knox, Y. Djoumbou, T. Jewison, A. C. Guo, Y. Liu, A. Maciejewski, D. Arndt, M. Wilson, V. Neveu, A. Tang, G. Gabriel, C. Ly, S. Adamjee, Z. T. Dame, B. Han, Y. Zhou, D. S. Wishart, DrugBank 4.0: Shedding new light on drug metabolism. *Nucleic Acids Res.* **42**, D1091–D1097 (2014).

57. J. J. Irwin, T. Sterling, M. M. Mysinger, E. S. Bolstad, R. G. Coleman, ZINC: A free tool to discover chemistry for biology. *J. Chem. Inf. Model.* **52**, 1757–1768 (2012).

58. S. Pasquini, M. De Rosa, V. Pedani, C. Mugnaini, F. Guida, L. Luongo, M. De Chiaro, S. Maione, S. Dragoni, M. Frosini, A. Ligresti, V. Di Marzo, F. Corelli, Investigations on the 4-quinolone-3-carboxylic acid motif. 4. Identification of NEW POTENT AND SELECTIVE LIGANDS FOR THE CANNABINOID TYPE 2 RECEPTOR WITH DIVERSE SUBSTITUTION PATTERNS AND ANTIHYPERALGESIC EFFECTS IN Mice. *J. Med. Chem.* **54**, 5444–5453 (2011).

59. S. Munro, K. L. Thomas, M. Abu-Shaar, Molecular characterization of a peripheral receptor for cannabinoids. *Nature* **365**, 61–65 (1993).

60. N. Gaborit, C. Larbouret, J. Vallaghe, F. Peyrusson, C. Bascoul-Mollevi, E. Crapez, D. Azria, T. Chardes, M.-A. Poul, G. Mathis, H. Bazin, A. Pèlegrin, Time-resolved fluorescence resonance energy transfer (TR-FRET) to analyze the disruption of EGFR/HER2 dimers: A new method to evaluate the efficiency of targeted therapy using monoclonal antibodies. *J. Biol. Chem.* **286**, 11337–11345 (2011).

61. S. Wind, D. Schnell, T. Ebner, M. Freiwald, P. Stopfer, Clinical pharmacokinetics and pharmacodynamics of afatinib. *Clin. Pharmacokinet.* **56**, 235–250 (2017).

62. H. Zhu, Y. Zhang, W. Li, N. Huang, A comprehensive survey of prospective structure-based virtual screening for early drug discovery in the past fifteen years. *Int. J. Mol. Sci.* **23**, 15961 (2022).

63. T. N. Doman, S. L. McGovern, B. J. Witherbee, T. P. Kasten, R. Kurumbail, W. C. Stallings, D. T. Connolly, B. K. Shoichet, Molecular docking and high-throughput screening for novel inhibitors of protein tyrosine phosphatase-1B. *J. Med. Chem.* **45**, 2213–2221 (2002).

64. I. Igashov, H. Stärk, C. Vignac, A. Schneuing, V. G. Satorras, P. Frossard, M. Welling, M. Bronstein, B. Correia, Equivariant 3D-conditional diffusion model for molecular linker design. *Nat. Mach. Intell.* **6**, 417–427 (2024).

65. Y. Jiang, G. Zhang, J. You, H. Zhang, R. Yao, H. Xie, L. Zhang, Z. Xia, M. Dai, Y. Wu, L. Li, S. Yang, PocketFlow is a data-and-knowledge-driven structure-based molecular generative model. *Nat. Mach. Intell.* **6**, 326–337 (2024).

66. W. Feng, L. Wang, Z. Lin, Y. Zhu, H. Wang, J. Dong, R. Bai, H. Wang, J. Zhou, W. Peng, B. Huang, W. Zhou, Generation of 3D molecules in pockets via a language model. *Nat. Mach. Intell.* **6**, 62–73 (2024).

67. O. Zhang, J. Zhang, J. Jin, X. Zhang, R. Hu, C. Shen, H. Cao, H. Du, Y. Kang, Y. Deng, F. Liu, G. Chen, C.-Y. Hsieh, T. Hou, ResGen is a pocket-aware 3D molecular generation model based on parallel multiscale modelling. *Nat. Mach. Intell.* **5**, 1020–1030 (2023).

68. L. Huang, T. Xu, Y. Yu, P. Zhao, X. Chen, J. Han, Z. Xie, H. Li, W. Zhong, K.-C. Wong, H. Zhang, A dual diffusion model enables 3D molecule generation and lead optimization based on target pockets. *Nat. Commun.* **15**, 2657 (2024).

69. J. Abramson, J. Adler, J. Dunger, R. Evans, T. Green, A. Pritzel, O. Ronneberger, L. Willmore, A. J. Ballard, J. Bambrick, S. W. Bodenstein, D. A. Evans, C.-C. Hung, M. O'Neill, D. Reiman, K. Tunyasuvunakool, Z. Wu, A. Žemgulytė, E. Arvaniti, C. Beattie, O. Bertolli, A. Bridgland, A. Cherepanov, M. Congreve, A. I. Cowen-Rivers, A. Cowie, M. Figurnov, F. B. Fuchs, H. Gladman, R. Jain, Y. A. Khan, C. M. R. Low, K. Perlin, A. Potapenko, P. Savy, S. Singh, A. Stecula, A. Thillaisundaram, C. Tong, S. Yakneen, E. D. Zhong, M. Zielinski, A. Žídek, V. Bapst, P. Kohli, M. Jaderberg, D. Hassabis, J. M. Jumper, Accurate structure prediction of biomolecular interactions with AlphaFold 3. *Nature* **630**, 493–500 (2024).

70. S. Passaro, G. Corso, J. Wohlwend, M. Reveiz, S. Thaler, V. R. Somnath, N. Getz, T. Portnoi, J. Roy, H. Stark, D. Kwabi-Addo, D. Beaini, T. Jaakkola, R. Barzilay, Boltz-2: Towards accurate and efficient binding affinity prediction. bioRxiv 659707 [Preprint] (2025); https://doi.org/10.1101/2025.06.14.659707.

71. H. Tang, J. Wang, Accurate site-specific folding via conditional diffusion based on AlphaFold3. *Proc. Natl. Acad. Sci. U.S.A.* **122**, e2521048122 (2025).

72. Y. Liu, H. Tang, T. Niu, J. Wang, A comparative study of deep learning and classical modeling approaches for protein–ligand binding pose and affinity prediction in coronavirus main proteases. *J. Chem. Inf. Model.* **66**, 731–743 (2025).

73. C. A. Lipinski, Lead- and drug-like compounds: The rule-of-five revolution. *Drug Discov. Today Technol.* **1**, 337–341 (2004).

74. N. M. O'Boyle, M. Banck, C. A. James, C. Morley, T. Vandermeersch, G. R. Hutchison, Open Babel: An open chemical toolbox. *J. Cheminform.* **3**, 33 (2011).

75. P. Refaeilzadeh, L. Tang, H. Liu, "Cross-validation," in *Encyclopedia of Database Systems*, L. Liu, M. T. ÖZsu, Eds. (Springer, 2009), pp. 532–538.

76. B. Ji, S. Liu, X. He, V. H. Man, X.-Q. Xie, J. Wang, Prediction of the binding affinities and selectivity for CB1 and CB2 ligands using homology modeling, molecular docking, molecular dynamics simulations, and MM-PBSA binding free energy calculations. *ACS Chem. Nerosci.* **11**, 1139–1158 (2020).

77. Y. Lecun, L. Jackel, L. Bottou, A. Brunot, C. Cortes, J. Denker, H. Drucker, I. Guyon, U. Muller, E. Sackinger, P. Simard, V. Vapnik, *Comparison Of Learning Algorithms For Handwritten Digit Recognition* (World Scientific, 1999).

78. F. Pedregosa, G. Varoquaux, A. Gramfort, V. Michel, B. Thirion, O. Grisel, M. Blondel, P. Prettenhofer, R. Weiss, V. Dubourg, J. Vanderplas, A. Passos, D. Cournapeau, M. Brucher, M. Perrot, É. Duchesnay, Scikit-learn: Machine Learning in Python. *J. Mach. Learn. Res.* **12**, 2825–2830 (2011).

79. M. F. Sanner, Python: A programming language for software integration and development. *J. Mol. Graph. Model.* **17**, 57–61 (1999).

80. F. Chollet, *Keras*. (Github, 2015).

81. M. Abadi, P. Barham, J. Chen, Z. Chen, A. Davis, J. Dean, M. Devin, S. Ghemawat, G. Irving, M. Isard, M. Kudlur, J. Levenberg, R. Monga, S. Moore, D. G. Murray, B. Steiner, P. Tucker, V. Vasudevan, P. Warden, M. Wicke, Y. Yu, X. Zheng, "TensorFlow: A system for

large-scale machine learning," in *Proceedings of the 12th USENIX conference on Operating Systems Design and Implementation*, (USENIX, 2016), pp. 265–283.

82. P. Willett, J. M. Barnard, G. M. Downs, Chemical Similarity Searching. *J. Chem. Inf. Comput. Sci.* **38**, 983–996 (1998).

83. M. Arjovsky, S. Chintala, L. Bottou, "Wasserstein generative adversarial networks," in *International Conference on Machine Learning* (PMLR, 2017), pp. 214–223.

84. G. R. Bickerton, G. V. Paolini, J. Besnard, S. Muresan, A. L. Hopkins, Quantifying the chemical beauty of drugs. *Nat. Chem.* **4**, 90–98 (2012).

85. H. Hadipour, Y. Y. Li, Y. Sun, C. Deng, L. Lac, R. Davis, S. T. Cardona, P. Hu, GraphBAN: An inductive graph-based approach for enhanced prediction of compound-protein interactions. *Nat. Commun.* **16**, 2541 (2025).

86. P. M. Shah, H. Zhu, Z. Lu, K. Wang, J. Tang, M. Li, DeepDTAGen: A multitask deep learning framework for drug-target affinity prediction and target-aware drugs generation. *Nat. Commun.* **16**, 5021 (2025).

87. H. Zhu, R. Zhou, D. Cao, J. Tang, M. Li, A pharmacophore-guided deep learning approach for bioactive molecular generation. *Nat. Commun.* **14**, 6234 (2023).

88. Q. Hu, C. Sun, H. He, J. Xu, D. Liu, W. Zhang, S. Shi, K. Zhang, H. Li, Target-aware 3D molecular generation based on guided equivariant diffusion. *Nat. Commun.* **16**, 7928 (2025).

89. K. Wu, Y. Xia, P. Deng, R. Liu, Y. Zhang, H. Guo, Y. Cui, Q. Pei, L. Wu, S. Xie, S. Chen, X. Lu, S. Hu, J. Wu, C.-K. Chan, S. Chen, L. Zhou, N. Yu, E. Chen, H. Liu, J. Guo, T. Qin, T.-Y. Liu, TamGen: Drug design with target-aware molecule generation through a chemical language model. *Nat. Commun.* **15**, 9360 (2024).

90. A. Ünlü, E. Çevrim, M. G. Yiğit, A. Sarıgün, H. Çelikbilek, O. Bayram, D. C. Kahraman, A. Olğaç, A. S. Rifaioglu, E. Banoğlu, T. Doğan, Target-specific de novo design of drug candidate molecules with graph-transformer-based generative adversarial networks. *Nat. Mach. Intell.* **7**, 1524–1540 (2025).

91. B. Malone, N. Urakova, E. J. Snijder, E. A. Campbell, Structures and functions of coronavirus replication-transcription complexes and their relevance for SARS-CoV-2 drug design. *Nat. Rev. Mol. Cell Biol.* **23**, 21–39 (2022).

92. Q. Hu, Y. Xiong, G.-H. Zhu, Y.-N. Zhang, Y.-W. Zhang, P. Huang, G.-B. Ge, The SARS-CoV-2 main protease (M^{pro}): Structure, function, and emerging therapies for COVID-19. *MedComm (2020)* **3**, e151 (2022).

93. Z. Jin, X. Du, Y. Xu, Y. Deng, M. Liu, Y. Zhao, B. Zhang, X. Li, L. Zhang, C. Peng, Y. Duan, J. Yu, L. Wang, K. Yang, F. Liu, R. Jiang, X. Yang, T. You, X. Liu, X. Yang, F. Bai, H. Liu, X. Liu, L. W. Guddat, W. Xu, G. Xiao, C. Qin, Z. Shi, H. Jiang, Z. Rao, H. Yang, Structure of M^{pro} from SARS-CoV-2 and discovery of its inhibitors. *Nature* **582**, 289–293 (2020).

94. Antiviral Drug Discovery, Polaris Hub (2025); https://polarishub.io/competitions/asap-discovery/antiviral-drug-discovery-2025.

95. M. Gassmann, F. Casagranda, D. Orioli, H. Simon, C. Lai, R. Klein, G. Lemke, Aberrant neural and cardiac development in mice lacking the ErbB4 neuregulin receptor. *Nature* **378**, 390–394 (1995).

96. R. S. Muraoka-Cook, M. Sandahl, C. Husted, D. Hunter, L. Miraglia, S.-M. Feng, K. Elenius, H. S. Earp III, The intracellular domain of ErbB4 induces differentiation of mammary epithelial cells. *Mol. Biol. Cell* **17**, 4118–4129 (2006).

97. J. Wang, P. Morin, W. Wang, P. A. Kollman, Use of MM-PBSA in reproducing the binding free energies to HIV-1 RT of TIBO derivatives and predicting the binding mode to HIV-1 RT of efavirenz by docking and MM-PBSA. *J. Am. Chem. Soc.* **123**, 5221–5230 (2001).

98. J. Wang, T. Hou, Develop and test a solvent accessible surface area-based model in conformational entropy calculations. *J. Chem. Inf. Model.* **52**, 1199–1212 (2012).

99. S. Jo, T. Kim, V. G. Iyer, W. Im, CHARMM-GUI: A web-based graphical user interface for CHARMM. *J. Comput. Chem.* **29**, 1859–1865 (2008).

100. W. L. Jorgensen, J. Chandrasekhar, J. D. Madura, R. W. Impey, M. L. Klein, Comparison of simple potential functions for simulating liquid water. *J. Chem. Phys.* **79**, 926–935 (1983).

101. J. A. Maier, C. Martinez, K. Kasavajhala, L. Wickstrom, K. E. Hauser, C. Simmerling, ff14SB: improving the accuracy of protein side chain and backbone parameters from ff99SB. *J. Chem. Theory Comput.* **11**, 3696–3713 (2015).

102. J. Wang, R. M. Wolf, J. W. Caldwell, P. A. Kollman, D. A. Case, Development and testing of a general amber force field. *J. Comput. Chem.* **25**, 1157–1174 (2004).

103. X. He, V. H. Man, W. Yang, T.-S. Lee, J. Wang, A fast and high-quality charge model for the next generation general AMBER force field. *J. Chem. Phys.* **153**, 114502 (2020).

104. C. J. Dickson, B. D. Madej, A. A. Skjevik, R. M. Betz, K. Teigen, I. R. Gould, R. C. Walker, Lipid14: The Amber Lipid Force Field. *J. Chem. Theory Comput.* **10**, 865–879 (2014).

105. C. I. Bayly, P. Cieplak, W. Cornell, P. A. Kollman, A well-behaved electrostatic potential based method using charge restraints for deriving atomic charges: The RESP model. *J. Phys. Chem.* **97**, 10269–10280 (1993).

106. M. J. Frisch, G. W. Trucks, H. B. Schlegel, G. E. Scuseria, M. A. Robb, J. R. Cheeseman, G. Scalmani, V. Barone, G. A. Petersson, H. Nakatsuji, X. Li, M. Caricato, A. V. Marenich, J. Bloino, B. G. Janesko, R. Gomperts, B. Mennucci, H. P. Hratchian, J. V. Ortiz, A. F. Izmaylov, J. L. Sonnenberg, Williams, F. Ding, F. Lipparini, F. Egidi, J. Goings, B. Peng, A. Petrone, T. Henderson, D. Ranasinghe, V. G. Zakrzewski, J. Gao, N. Rega, G. Zheng, W. Liang, M. Hada, M. Ehara, K. Toyota, R. Fukuda, J. Hasegawa, M. Ishida, T. Nakajima, Y. Honda, O. Kitao, H. Nakai, T. Vreven, K. Throssell, J. A. Montgomery Jr., J. E. Peralta, F. Ogliaro, M. J. Bearpark, J. J. Heyd, E. N. Brothers, K. N. Kudin, V. N. Staroverov, T. A. Keith, R. Kobayashi, J. Normand, K. Raghavachari, A. P. Rendell, J. C. Burant, S. S. Iyengar, J. Tomasi, M. Cossi, J. M. Millam, M. Klene, C. Adamo, R. Cammi, J. W. Ochterski, R. L. Martin, K. Morokuma, O. Farkas, J. B. Foresman, D. J. Fox. Gaussian 16 (Wallingford, CT, 2016).

107. J. Wang, W. Wang, P. A. Kollman, D. A. Case, Automatic atom type and bond type perception in molecular mechanical calculations. *J. Mol. Graph. Model.* **25**, 247–260 (2006).

108. I. Y. B.-S. D. A. Case, S. R. Brozell, D. S. Cerutti, T. E. Cheatham, III, V. W. D. Cruzeiro, T. A. Darden, R. E. Duke, D. Ghoreishi, M. K. Gilson, H. Gohlke, A.W. Goetz, D. Greene, R Harris, N. Homeyer, S. Izadi, A. Kovalenko, T. Kurtzman, T. S. Lee, S. LeGrand, P. Li, C. Lin, J. Liu, T. Luchko, R. Luo, D. J. Mermelstein, K. M. Merz, Y. Miao, G. Monard, C. Nguyen, H. Nguyen, I. Omelyan, A. Onufriev, F. Pan, R. Qi, D. R. Roe, A. Roitberg, C. Sagui, S. Schott-Verdugo, J. Shen, C. L. Simmerling, J. Smith, R. Salomon-Ferrer, J. Swails, R. C. Walker, J. Wang, H. Wei, R. M. Wolf, X. Wu, L. Xiao, D. M. York, P. A. Kollman, AMBER 16 (University of California, San Francisco, 2016).

109. A. W. Götz, M. J. Williamson, D. Xu, D. Poole, S. Le Grand, R. C. Walker, Routine Microsecond Molecular Dynamics Simulations with AMBER on GPUs. 1. Generalized Born. *J. Chem. Theory Comput.* **8**, 1542–1555 (2012).

110. R. Salomon-Ferrer, A. W. Götz, D. Poole, S. Le Grand, R. C. Walker, Routine Microsecond Molecular Dynamics Simulations with AMBER on GPUs. 2. Explicit Solvent Particle Mesh Ewald. *J. Chem. Theory Comput.* **9**, 3878–3888 (2013).

111. J.-P. Ryckaert, G. Ciccotti, H. J. C. Berendsen, Numerical integration of the Cartesian Equations of Motion of a System with constraints: molecular dynamics of N-Alkanes. *J. Comput. Phys.* **23**, 327–341 (1977).

112. L. Xu, H. Sun, Y. Li, J. Wang, T. Hou, Assessing the performance of MM/PBSA and MM/GBSA methods. 3. The impact of force fields and ligand charge models. *J. Phys. Chem. B* **117**, 8408–8421 (2013).

113. Y. Sun, X. He, T. Hou, L. Cai, V. H. Man, J. Wang, Development and test of highly accurate endpoint free energy methods. 1: Evaluation of ABCG2 charge model on solvation free energy prediction and optimization of atom radii suitable for more accurate solvation free energy prediction by the PBSA method. *J. Comput. Chem.* **44**, 1334–1346 (2023).

114. A. Jakalian, B. L. Bush, D. B. Jack, C. I. Bayly, Fast, efficient generation of high-quality atomic charges. AM1-BCC model: I. Method. *J. Comput. Chem.* **21**, 132–146 (2000).

115. A. Jakalian, D. B. Jack, C. I. Bayly, Fast, efficient generation of high-quality atomic charges. AM1-BCC model: II. Parameterization and validation. *J. Comput. Chem.* **23**, 1623–1641 (2002).

116. H. H. Loeffler, J. Michel, C. Woods, FESetup: Automating setup for alchemical free energy simulations. *J. Chem. Inf. Model.* **55**, 2485–2490 (2015).

117. W. L. Jorgensen, The many roles of computation in drug discovery. *Science* **303**, 1813–1818 (2004).

118. D. Melck, L. De Petrocellis, P. Orlando, T. Bisogno, C. Laezza, M. Bifulco, V. Di Marzo, Suppression of nerve growth factor Trk receptors and prolactin receptors by endocannabinoids leads to inhibition of human breast and prostate cancer cell proliferation. *Endocrinology* **141**, 118–126 (2000).

119. M. Jung, R. Calassi, M. Rinaldi-Carmona, P. Chardenot, G. Le Fur, P. Soubrié, F. Oury-Donat, Characterization of CB1 receptors on rat neuronal cell cultures: Binding and functional studies using the selective receptor antagonist SR 141716A. *J. Neurochem.* **68**, 402–409 (1997).

120. Y. Cheng, W. H. Prusoff, Relationship between the inhibition constant (K1) and the concentration of inhibitor which causes 50 per cent inhibition (I50) of an enzymatic reaction. *Biochem. Pharmacol.* **22**, 3099–3108 (1973).
